# Therapeutic Intervention in Multiple Sclerosis with Alpha B-Crystallin: A Randomized Controlled Phase IIa Trial

**DOI:** 10.1371/journal.pone.0143366

**Published:** 2015-11-23

**Authors:** Johannes M. van Noort, Malika Bsibsi, Peter J. Nacken, Richard Verbeek, Edna H.G. Venneker

**Affiliations:** Delta Crystallon, Leiden, The Netherlands; University of Münster, GERMANY

## Abstract

As a molecular chaperone and activator of Toll-like receptor 2-mediated protective responses by microglia and macrophages, the small heat shock protein alpha B-crystallin (HspB5) exerts therapeutic effects in different animal models for neuroinflammation, including the model for multiple sclerosis (MS). Yet, HspB5 can also stimulate human antigen-specific memory T cells to release IFN-γ, a cytokine with well-documented detrimental effects during MS. In this study, we explored in a Phase IIa randomized clinical trial the therapeutic application of HspB5 in relapsing-remitting MS (RR-MS), using intravenous doses sufficient to support its protective effects, but too low to trigger pathogenic memory T-cell responses. These sub-immunogenic doses were selected based on *in vitro* analysis of the dose-response profile of human T cells and macrophages to HspB5, and on the immunological effects of HspB5 in healthy humans as established in a preparatory Phase I study. In a 48-week randomized, placebo-controlled, double-blind Phase IIa trial, three bimonthly intravenous injections of 7.5, 12.5 or 17.5 mg HspB5 were found to be safe and well tolerated in RR-MS patients. While predefined clinical endpoints did not differ significantly between the relatively small groups of MS patients treated with either HspB5 or placebo, repeated administration especially of the lower doses of HspB5 led to a progressive decline in MS lesion activity as monitored by magnetic resonance imaging (MRI), which was not seen in the placebo group. Exploratory linear regression analysis revealed this decline to be significant in the combined group receiving either of the two lower doses, and to result in a 76% reduction in both number and total volumes of active MRI lesions at 9 months into the study. These data provide the first indication for clinical benefit resulting from intervention in RR-MS with HspB5.

***Trial Registration***: ClinicalTrials.gov Phase I: NCT02442557; Phase IIa: NCT02442570

## Introduction

MS is a chronic demyelinating disease of the central nervous system (CNS), affecting an estimated 2.3 million people worldwide. While the cause of MS remains obscure, the recurrent development of inflammatory lesions in the CNS leading to demyelination and axonal damage is a key feature of the condition. Equally consistent are findings of both focal and diffuse biochemical and histological abnormalities in the CNS, in association with signs of oxidative stress [1]. One of these abnormalities is the selective accumulation of the stress-inducible small heat-shock protein HspB5 in myelin-forming oligodendrocytes during MS, which is part of an anti-apoptotic response by these cells to oxidative stress [2]. Several studies have clarified that HspB5 levels regularly increase up to 20-fold during MS [3–8], also in areas of the CNS that are not yet visibly affected by inflammation and myelin damage [9]. It has now gradually become clear that this remarkable accumulation of HspB5 serves broader functions that just protection from oxidative stress.

Over the last decade, evidence has accumulated for the broad neuroprotective and anti-inflammatory activity of HspB5, both as an endogenous protective factor in the CNS and as an exogenous protein with therapeutic qualities [10–19]. HspB5 counteracts neuronal and glial cell apoptosis, mitigates inflammation, reduces tissue damage, and promotes recovery in a variety of animal models for neuroinflammatory disorders such as autoimmune demyelination, stroke, spinal cord injury and optic nerve damage. HspB5 exerts these beneficial effects as the result of its ability to act as a molecular chaperone [17] as well as an activator of Toll-like receptor (TLR) 2-mediated anti-inflammatory, neuroprotective and tolerogenic responses in CD14-expressing cells such as microglia and macrophages [20, 21].

As previously documented by us using genome-wide microarray transcript profiling, HspB5 induces in human microglia and macrophages the production of large amounts of IL-10, some IL-6 and TNF-α, but no IL-1β or IL-12, consistent with an M2-like protective response profile [20, 21]. In addition, HspB5 induces a cocktail of tolerogenic human macrophage factors including CD274 (also known as PD-1 ligand), the TIGIT ligand CD155, the adenosine A2A receptor, the TGF-β-releasing integrin αVβ8, the tryptophan-degrading enzymes indoleamine-2,3-dioxygenase-1 and kynureninase, tumor necrosis factor-α-stimulated gene 6, Epstein-Barr virus-induced 3 and the combination of IL-15 and the IL-15 receptor.

Apart from exerting beneficial effects, however, production and release of large amounts of HspB5 by oligodendrocytes during MS also poses a risk. The protein not only ends up in surrounding microglia but also in perivascular macrophages and B cells that act as functional antigen-presenting cells [22, 23]. Since a normal adult human immune repertoire contains HspB5-reactive memory T cells that engage in routine immune surveillance of the CNS as much as any other memory T cell [24], HspB5 produced by oligodendrocytes can ultimately activate these T cells when it is produced at sufficiently high concentrations. As shown before [22], and as confirmed in the present study, when HspB5-reactive human T cells do become activated, they will start to release IFN-γ which is a cytokine with well-known detrimental effects in MS patients [25]. IFN-γ profoundly changes the otherwise protective TLR2-mediated response to HspB5 of microglia and macrophages, and turns it into a destructive classical response [23]. HspB5-induced IL-10 production is inhibited by IFN-γ, and production of TNF-α, IL-1β, IL-12 and reactive oxygen species is strongly enhanced. By subverting the ongoing signaling function of HspB5 in this way, release of IFN-γ by HspB5-reactive T cells within the CNS can spark the development of inflammatory demyelinating lesions during MS.

This dual function of HspB5 during autoimmune demyelination suggests that its combined anti-inflammatory and neuroprotective actions may be exploited for therapeutic purposes in humans provided that the dose at which it is administered remains below the concentration threshold required for T-cell activation. At such sub-immunogenic doses, intravenous HspB5 is likely to promote tolerance to itself as well, eventually eliminating the potentially pathogenic effects of T-cell activation altogether. To select doses of intravenous HspB5 that would be suitable for such an approach in humans, we first performed a Phase I dose-escalation study, as well *in vitro* tests of the dose-response profile of human T cells and macrophages to HspB5. As shown here, the combined data from these studies led to the definition of a sub-immunogenic dose range of HspB5 that was considered suitable for testing in a subsequent randomized, placebo-controlled, double-blind Phase IIa study in RR-MS patients. The primary objective of this study was to evaluate safety and tolerability of periodically repeated intravenous administrations of HspB5 at these dose levels. Secondary objectives included evaluation of the pharmacokinetic profile of HspB5, and its immunological and clinical effects. Results presented here demonstrate the safety and tolerability of HspB5 treatment in humans, and provide the first indication for clinical benefit in RR-MS patients.

## Materials and Methods

Both Phase I and II studies were performed according to International Conference on Harmonisation of Good Clinical Practice (ICH-GCP) requirements, and in accordance with the Declaration of Helsinki. For both Phase I and Phase IIa studies, all subjects were informed of the nature and aims of the study, and had given their written consent to participate in this study. Written approval for the Phase I and IIa studies was obtained from the Ethics Committees listed below prior to commencement of, and/or during the study. Phase I study: until 8 July 2010: STEG/METC Almere, Louis Armstrongweg 78, 1311 RL Almere, The Netherlands. Between 8 July 2010 –August 2010: Centrale Commissie Mensgebonden Onderzoek (CCMO), Parnassusplein 5, Den Haag, The Netherlands. From August 2010 onwards: Stichting Beoordeling Ethiek Biomedisch Onderzoek (Stichting BEBO), PO Box 1004, 9400 BA Assen, The Netherlands. Phase IIa study: Republic of Bulgaria, Ministry of Health, Ethics Committee for Multicenter Trials, Sofia, 5 Sveta Nedelya Square. The protocols (S1 and S2 Protocols), the Investigator’s Brochure, the patient information sheet and consent form were submitted by the principal investigator(s) to the Independent Ethics Committee (IEC) or Institutional Review Board (IRB). A copy of the written approval was provided to the sponsor. Any amendments to the protocol or patient information and consent form were submitted to the IEC/IRB of the investigational center, and written approval was obtained for significant amendments prior to implementation.

### Study design Phase I

The first-in-man Phase I study was a double-blind, randomized, placebo-controlled, dose-escalation study aimed at testing safety, pharmacokinetic profile and immunological effects of intravenous HspB5 in healthy human subjects. The study was registered under EudraCT 2009-016817-68. After the study was concluded, it was registered at ClinicalTrials.gov under NCT02442557; this was not done previously since the study was performed in Europe. The study consisted of two parts. In the first part, four groups of 10 healthy subjects were randomized 8:2 to receive either a single intravenous dose of HspB5 of 4, 12.5, 25 or 37.5 mg, respectively (n = 8 for each dose level), or an identical volume of PBS as placebo (n = 8 in total for the four groups). A total of 32 subjects therefore received HspB5 in this first part of the Phase I study. In the second part, three groups of 12 subjects were randomized 9:3 to receive three consecutive daily intravenous doses of HspB5 of 10, 12.5 or 37.5 mg (n = 9 for each dose level), or three consecutive daily administrations of placebo (n = 9 in total). The next higher dose was given only once safety data on the previous dose group up to 4 days (part 1) or 8 days (part 2) were reviewed and raised no safety concerns. A total of 27 subjects therefore received HspB5 in this second part of the Phase I study.

#### Inclusion and exclusion criteria, randomization and measurements

Subjects were healthy male or female volunteers aged 18–55 years, body-mass index between 20.0 and 28.0 kg/m^2^, and in general good health. Further details on inclusion and exclusion criteria, randomization and measurements performed can be found in the S1 Protocol. The study was performed between December 2009 and October 2010 in a Phase I study center in Utrecht, The Netherlands.

Given that this was the first study of intravenous HspB5 in humans, sample sizes were based on medical and practical grounds rather than statistical grounds. The safety population comprised of all subjects who received at least one dose of HspB5 or placebo. Vital signs (supine blood pressure, heart rate, body temperature and respiratory rate) as well as 12-lead EEG, hematology, biochemistry, and urinalysis parameters were recorded. AEs were coded using the Medical Dictionary for Regulatory Activities (MedDRA).

### Study design Phase IIa

A randomized, double-blind, placebo-controlled, exploratory, dose-ranging Phase IIa study was subsequently performed in RR-MS patients to evaluate the safety and tolerability, pharmacokinetics, immunological and clinical effects of intravenous HspB5. The study was registered under EudraCT 2011-004475-36. After the study was concluded, it was registered at ClinicalTrials.gov under NCT02442570; this was not done previously since the study was performed in Europe (see S1 CONSORT Checklist). At entry, patients were randomized 1:1:1:1 to receive an intravenous bolus injection of either 7.5 mg, 12.5 mg, 17.5 mg HspB5 or an identical volume of PBS as placebo. A placebo-controlled design rather than the use of a comparator drug was chosen to avoid the double-dummy design that would otherwise have been required to maintain blinding, given that no comparator drug can be given by intravenous bolus injection. The use of a placebo was considered acceptable since for MS patients involved in the study, participation offered a treatment option that was otherwise not available. Administration of HspB5 or placebo was repeated twice with 2-month intervals during the treatment period of 24 weeks, after which the patients entered a follow-up period of an additional 24 weeks. During the treatment period, patients returned to the clinic for examinations at weeks 1, 2 and 4, and monthly thereafter. The follow-up period involved clinical examination after 36 and 48 weeks. The primary analysis was performed on data collected in the treatment period, and was performed after all patients had completed 24 weeks into the study. An additional analysis was performed once all patients had completed the full 48 weeks of the treatment and follow-up periods. Patients, clinical site personnel as well as CRO personnel involved in the conduct of the study remained blinded during the follow-up period. After 12 and 24 patients completed 4 weeks into the study, and after 24 patients had completed 12 weeks of follow-up, a partially blinded safety review was conducted by an independent drug safety monitoring board to verify safety of the intervention in MS patients. At no point did this lead to any recommendation to change methods used in the study, or to terminate it.

#### Inclusion and exclusion criteria, randomization and measurements

**Fig 1:**
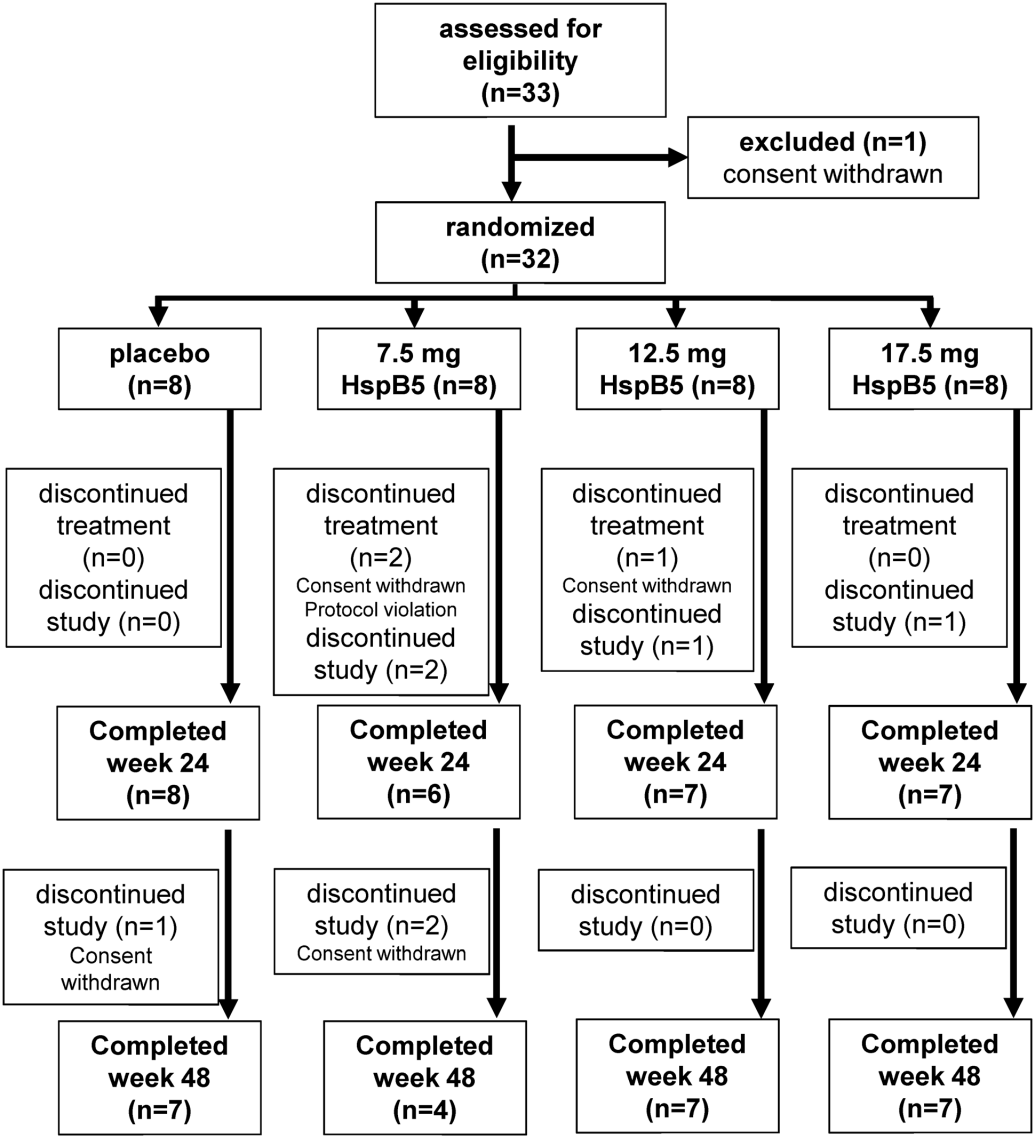
Phase IIa study profile.

At screening, information on gender, date of birth, race, body weight, height and smoking habits were recorded, along with a review of the patient’s complete medical history. In addition, all medications used within 3 months prior to entry into the study, both prescribed and over-the-counter, were documented. Main inclusion criteria were a diagnosis of clinically definite, RR-MS according to the revised McDonald criteria [26], or one or more Gd^+^ T1 MRI lesion(s) and neurologically stable at the time of screening, having had at least one clinical relapse over the previous year or two relapses over the past two years, or one or more gadolinium-enhancing MRI lesion(s) at the time of screening, an EDSS score ≤ 5.5, and an age between 18 and 55 years. Main exclusion criteria were primary progressive multiple sclerosis, systemic corticosteroid treatment for at least 3 consecutive days less than 30 days before screening, plasmapheresis or intravenous gammaglobulins less than 2 months before screening, treatment with natalizumab less than one year before screening, previous immunosuppressive treatment (e.g. cyclophosphamide or mitoxantrone), previous treatment with any leukocyte-targeting monoclonal antibody (such as rituximab, alemtuzumab, daclizumab), or previous treatment with oral immune-modulatory agents (cladribine, fingolimod, laquinimod, fumarate). Previous or concomitant use of interferon-beta or glatiramer acetate was allowed. Further details on inclusion and exclusion criteria, randomization and measurements performed can be found in S2 Protocol. Sample sizes were based on medical and scientific grounds rather than statistical grounds. The study was performed between September 2012 and June 2014 in five clinical centers in Bulgaria. The patient disposition of this Phase IIa trial is illustrated in Fig 1.

### Blinding procedure and study drug administration in the Phase IIa study

Each patient whose eligibility had been confirmed was assigned a patient screening number at the site level, and a sequential randomization number issued by phone by the assisting clinical research organization (CRO), coupled to a treatment code that was independently prepared by the CRO using SAS^®^. To secure parallel design, codes covering the four different treatment groups were grouped in blocks of four, in random order. Sealed treatment codes were stored in the secured investigator’s file at the investigator’s site. Un-blinding of the codes was done only after closing of the database. At regular intervals and after completion of the study, the code envelopes were checked by the monitor for intactness.

Recombinant human HspB5 used for both studies was prepared and labeled according to the guidelines for Good Manufacturing Practice, ICH-GCP, and local regulatory requirements, and stored at -20°C. For study drug administration, syringes containing 10 mL of HspB5 at concentrations of 0.75, 1.25 or 1.75 mg/mL in PBS were prepared, along with syringes containing 10 mL PBS. They were labelled with the patient’s randomization number, the patient’s initials, and the date and time of preparation. In order to maintain blinding, all study drug syringes were covered with non-transparent foil before leaving the pharmacy of the clinical unit, to obscure any difference in the appearance of the solutions. Prior to use, syringes were stored at 2–8°C for a maximum of 3 h. Following intravenous injection of the HspB5 solution or PBS into the arm over a period of 30 seconds, the catheter used for this purpose was flushed with 10 mL PBS. Patients received such an intravenous bolus injection every two months, for a total of three times during the study.

### Safety evaluation

The safety population comprised all subjects who received at least one dose of either HspB5 or placebo. The overall incidence of AEs, the incidence of related as well as the incidence of serious AEs recorded during the study were categorized by body system and preferred term using MedDRA. Local tolerability was evaluated based on the overall incidence of AEs per treatment group per symptom. Vital signs (supine blood pressure and heart rate), 12-lead ECG and body temperature were recorded, along with hematology, biochemistry and urinalysis parameters.

### Pharmacokinetics, T-cell and serum antibody responses

Personnel performing the assays to collect data on serum concentrations of HspB5 and on T-cell and antibody responses were blinded to treatment of the subjects throughout all analytical work during the Phase I as well as Phase IIa study.

Pharmacokinetic parameters including maximum serum concentration of HspB5 and half-life, as well as dose proportionality and the effects of multiple administrations were evaluated using serum samples collected over a period of 24 h. Concentrations of HspB5 were determined following tryptic digestion of serum samples and solid-phase extraction of the digest, by absolute quantitation of a unique tryptic fragment of HspB5 by mass spectrometry. These evaluations were performed by Alphalyse A/S, Odense, Denmark.

Proliferative CD4^+^ and CD45RO^+^ T-cell responses to HspB5 at different concentrations between 10 and 200 μg/mL, and to tetanus toxoid (NVI Bilthoven, The Netherlands) as a control antigen at 0.2 μg/mL, were evaluated using a carboxyfluorescein succinimidyl ester (CFSE) assay. Duplicate 10-mL blood samples were collected in heparin tubes to prevent blood clotting, and kept at room temperature until they were processed. No later than 8 h after sampling, PBMC were isolated from blood samples, labelled with 1 μM CFSE (Invitrogen, Waltham, MA), and stimulated with antigen or supplied with RPMI1640 culture medium alone. After 9 days under standard culturing conditions in 96-well plates, cells were stained with fluorescently labelled antibodies to either CD4 or CD45RO (Becton Dickinson, Franklin Lakes, NJ) and analyzed by flowcytometry. The percentage of CFSE^dim^, CD4^+^ or CD45RO^+^ T cells in the samples was used to quantitate proliferation. All data points were collected using triplicate samples as much as possible, and at least in duplicate, with each sample comprising the combined cells of six individual culture wells. Statistical significance of changes in T-cell reactivity were evaluated by a paired, one-sided Student’s t test. Assays in the context of the Phase I study were performed by Delta Crystallon BV, Leiden, the Netherlands, those in the context of the Phase IIa study by the Central Reference Laboratory for Immunology, at the National Center for Infectious and Parasitic Diseases, Sofia, Bulgaria.

Quantitation of total serum IgG antibodies against HspB5 was performed by standard ELISA on plates coated with HspB5, using peroxidase-conjugated rabbit anti-human IgG (Sigma Aldrich, St. Louis, MO) as a secondary antibody. Pooled human male AB serum (Sigma Aldrich) was used as a reference standard to normalize IgG titers as evaluated on different plates, or on different days. For both studies, antibody assessments were performed by Delta Crystallon BV, Leiden, the Netherlands. A Wilcoxon signed rank test was performed to evaluate changes from baseline within groups.

### MRI evaluation

Gd^+^ T1-weighted and T2-weighted brain MRI scans were acquired at each visit, except on day 1 and week 1 after the first administration. A detailed MRI scanning protocol including, but not limited to field strength, slice thickness and gap, pixel size, brain coverage, core brain sequences, gadolinium dose, and infusion speed and time between gadolinium dosing and post-dose imaging was prepared prior to start of the study. All MRIs were performed at a central facility in Bulgaria. All MRI readings were performed by the Imaging Analysis Center at the VU University Medical Center, Amsterdam, The Netherlands, under the supervision of Dr. F. Barkhof. The pre-defined effect endpoint was the cumulative number of newly-emerging Gd^+^ MRI lesions on T1-weighted scans between weeks 4 and 24, and between weeks 4 and 48 after treatment. The full analysis set of patients was used for the evaluation of MRI parameters. Comparisons between dose groups and placebo were performed using Wilcoxon rank sum test. Post-hoc exploratory evaluation of trends of change in MRI parameters for each treatment group were performed using linear regression analysis using Prism software version 5 (GraphPad Inc. CA).

### Clinical evaluation

A confirmed clinical relapse was defined as the occurrence of new symptoms, or worsening of previously stable or improving symptoms, not associated with fever, lasting for more than 24 h, and accompanied by an increase of at least half a point in the EDSS score, or 1 point in the score for at least one of the functional systems, excluding the bowel and bladder and mental systems. A clinical relapse as defined above was considered to be a new relapse when the onset of symptoms occurred 30 days or more after the onset of a previous relapse. Neurologic deterioration that was classified by the treating physician as a relapse but did not fulfil these criteria was documented as an unconfirmed clinical relapse. Clinical relapses were treated with systemic corticosteroids if this was deemed by the treating physician to be in the best interest of the patient. Dose and duration of treatment was at the discretion of the investigator. The predefined effect endpoint was the number of clinical relapses between week 0 and week 24, and between week 0 and week 48.

The EDSS was used to quantify the level of disability, and the impact of MS on the quality of life was evaluated using the MSIS-29 version 2 questionnaire. Predefined effect endpoints were changes from baseline in the EDSS and MSIS-29 score between week 0 and week 24, and between week 0 and week 48. Changes from baseline in EDSS and MSIS-29 scores were assessed using the Wilcoxon signed rank test. Comparisons between dose groups and placebo were performed using Wilcoxon rank sum test.

All analysis were performed on the full analysis set (FAS) consisting of all subjects who received at least one dose of HspB5 or placebo, and for whom data of at least one assessment beyond baseline was available.

## Results

### Preclinical and clinical dose-finding studies

**Fig 2:**
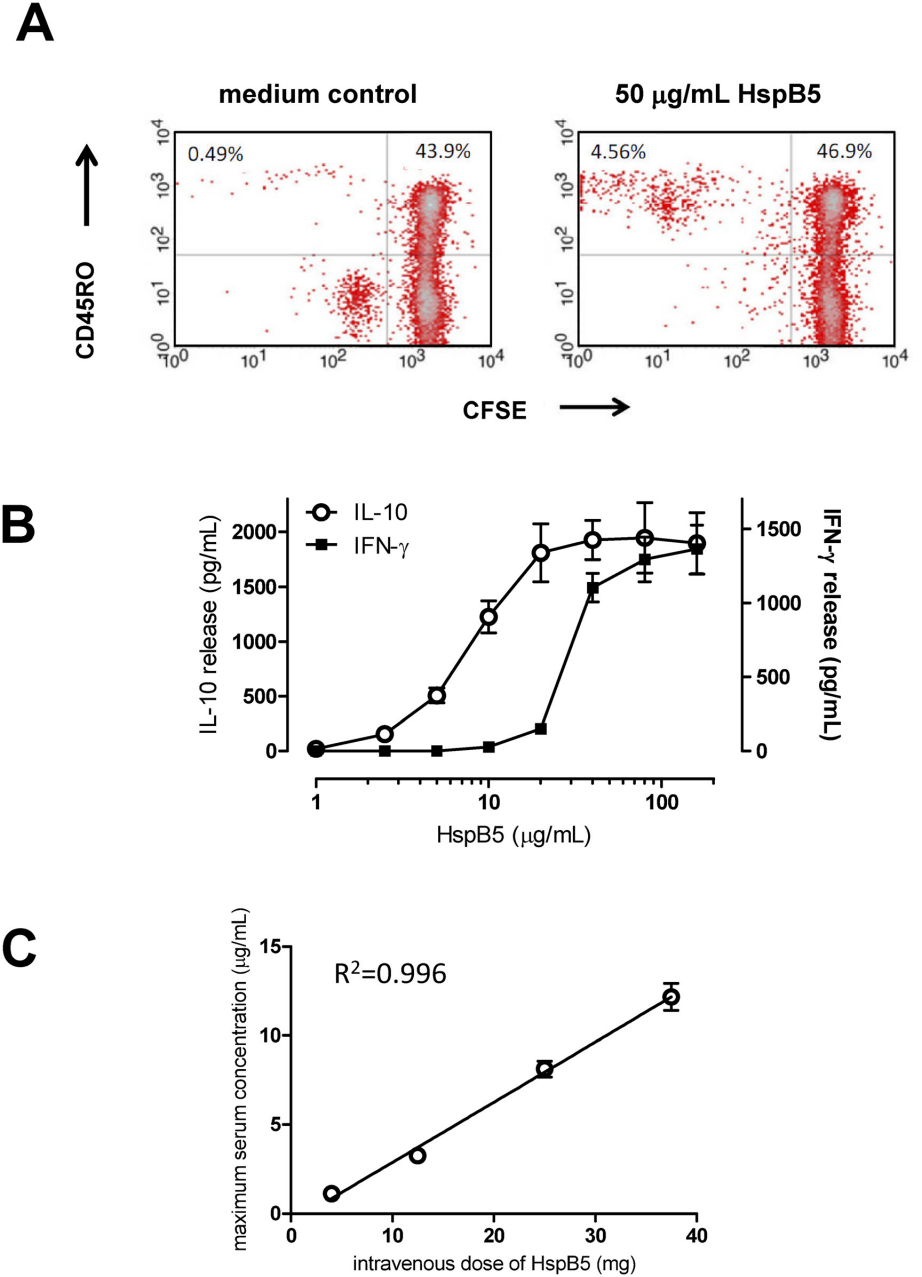
Selection of therapeutic intravenous doses of HspB5 in humans. Therapeutic application of HspB5 in humans relies on activation of M2-like protective microglia and macrophage responses, while avoiding pathogenic IFN-γresponses by memory T cells. In Fig 2A, the existence of a substantial HspB5-reactive memory T-cell repertoire in humans is illustrated by a representative fresh PBMC sample from a healthy subject. Proliferative responses to HspB5 of CD45RO^+^ memory T cells in this sample are reflected by proliferation-induced dilution of the fluorescent tracer CFSE over 9 days in culture. With CFSE^dim^ CD45RO^+^ T cells representing proliferated memory T cells, Fig 1A illustrates that a substantial part of all human memory T cells, an estimated one in about every 4,000 cells, do indeed respond to HspB5. In Fig 2B, it is illustrated, again with a representative example, that human T cells are triggered by HspB5 to release IFN-γ, but only when the HspB5 concentration exceeds a threshold of about 20 μg/mL. The protective macrophage response, on the other hand, is already activated by markedly lower μg/mL-concentrations of HspB5, as exemplified by IL-10 secretion as marker for protective responses. At these low sub-immunogenic concentrations, HspB5 can therefore be therapeutically exploited in humans. In Fig 3C, it is shown that single intravenous doses of up to 37.5 mg HspB5 leads to such sub-immunogenic peak serum concentrations in humans, remaining well below the 20-μg/mL threshold. Fig 3C shows the mean ± standard deviation peak serum concentrations of HspB5 found in groups of 8 healthy subjects 10–20 min after receiving varying amounts of intravenous HspB5 during the Phase I study.

As explained in the introduction, key to successful therapeutic application of HspB5 in humans is the definition of intravenous doses at which its anti-inflammatory and neuroprotective actions are not thwarted by an antigen-specific response of IFN-γ-secreting HspB5-reactive memory T cells that are part of a normal human immune repertoire. As recently shown by us, IFN-γ changes the otherwise IL-10-dominated protective TLR2-mediated of microglia and macrophages to HspB5 into a destructive classical response that is dominated by the production of large amounts of TNF-α, IL-1β, IL-12 and reactive oxygen species [23]. In Fig 2A, the presence of a substantial HspB5-reactive memory T-cell population in the human circulation is illustrated by proliferation-induced dilution of the fluorescent tracer CFSE in CD45RO^+^ memory T cells from a healthy subject, as a representative example. Upon screening 76 healthy subjects prior to treatment in our Phase I study, a mean ± standard deviation (SD) of 6.4 ± 7.6% of all memory T cells were found to accumulate as CFSE^dim^ cells after 9 days in culture with HspB5, including both CD4^+^ and CD8^+^ memory T cells in variable ratios. On average, the mean fluorescence signal in this population was reduced about 250-fold relative to the non-proliferated CFSE^bright^ population, which is indicative for about 8 cycles of division. This clarifies that in humans, on average about one in every 4,000 peripheral memory T cells responds to HspB5.

As illustrated in Fig 2B, and in line with our previous findings [22], this natural repertoire of HspB5-reactive human memory T-cells consistently release significant amounts of IFN-γ in response to their antigen. At least *in vitro*, however, this occurs only when the concentration of HspB5 exceeds a threshold of approximately 20 μg/mL. The dose-response profile of human macrophages to HspB5, which in the absence of IFN-γ leads to release of protective factors such as IL-10, indicated a markedly greater sensitivity of the TLR2-mediated response to HspB5 by these cells. Already at HspB5 concentrations well below 20 μg/mL, macrophages start to release protective factors such as IL-10 (Fig 2B). No marked differences were observed in these dose-response relationships for cells obtained from different healthy donors. This comparison indicates that at low μg/mL concentrations, HspB5 selectively activates protective macrophage responses without already triggering release of IFN-γ by memory T cells too.

In a dose-finding randomized, placebo-controlled, double-blind Phase I study, we next tested the effects of both single and three consecutive daily intravenous administrations of HspB5 in escalating doses starting at 4 mg, and going up to 37.5 mg. As summarized in Fig 2C, pharmacokinetic analysis demonstrated that such doses lead to maximum serum concentrations of HspB5 between means ± SD of 1.1± 0.27 and 12.1± 2.13 μg/mL, respectively, with a half-life of approximately 60–75 min at all doses tested. These peak serum concentrations are therefore well below the 20-μg/mL threshold for T-cell activation, but do reach the low μg/mL-levels that are adequate for triggering protective responses by macrophages. Of note is further that the systemic dose of HspB5 that has been repeatedly been found to exert therapeutic effects in animal models is 10 μg [10–17], which in normal mice will result in maximum serum concentrations between approximately 4 and 7 μg/mL, again within the same sub-immunogenic range.

**Fig 3:**
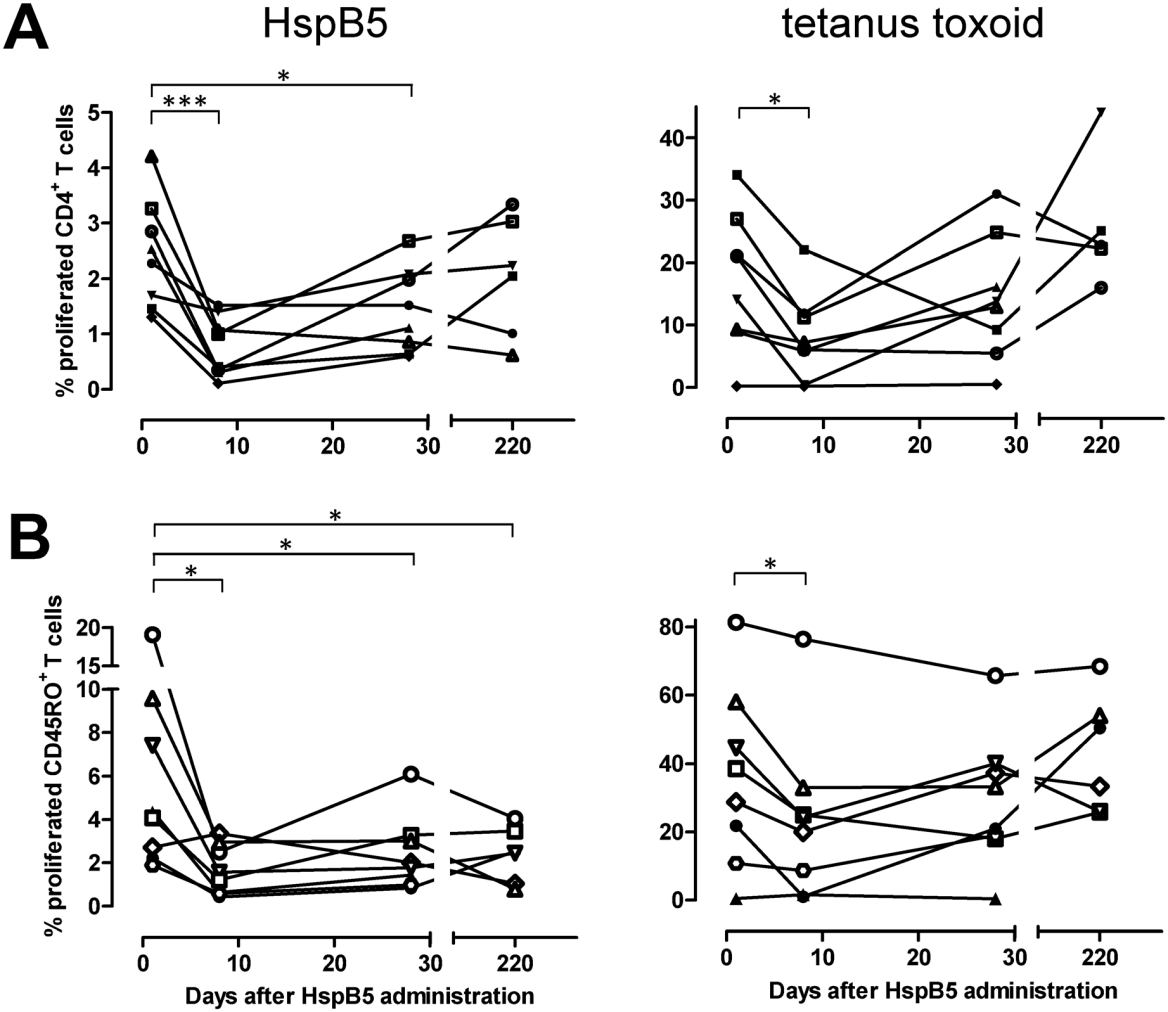
Effects of intravenous HspB5 on peripheral T-cell reactivity to HspB5 itself, and tetanus toxoid. Fig 3 illustrates the proliferative response *in vitro* of CD4^+^ (A) as well as CD45RO^+^ memory T-cells (B) to either 50 μg/mL HspB5 or 0.2 μg/mL tetanus toxoid as a control antigen, at various time points after a single intravenous dose of 12.5 mg HspB5 in 8 healthy subjects, as evaluated using a CFSE assay during Phase I. Percentages of proliferated CFSE^dim^ cells are expressed relative to all other lymphocytes detected by flowcytometry in the sample after 9 days in culture, including CD4/CD45RO-negative cells. The results not only confirm that following an intravenous dose of 12.5 mg HspB5, its levels remain at sub-immunogenic levels, but even illustrate significant suppressive effect on *in vitro* T-cell reactivity that is markedly more lasting for HspB5-reactive T cells than for tetanus toxoid-reactive T cells. Background proliferation found in cultures without any antigen was well below 1% in all cases. *: p< 0.05; ***: p<0.005.

To confirm that HspB5 at intravenous doses between 4 and 37.5 mg remains at sub-immunogenic levels in humans, we tested proliferation of both CD4^+^ and CD45RO^+^ memory T cells against HspB5 and the control antigen tetanus toxoid in all subjects at various points in time after administration of either HspB5 at different dose levels, or placebo. This evaluation confirmed that none of the single doses of HspB5 led to any significant increase in T-cell reactivity for a period of at least 28 days, similar to the lack of any T-cell activation in response to placebo. Intriguingly, data obtained after administration of a 12.5-mg dose even indicated a significant and sustained reduction in CD4^+^ (p = 0.0004) as well as CD45RO^+^ (p = 0.021) T-cell reactivity to HspB5, as illustrated in Fig 3. Especially for CD45RO^+^ memory T cells, HspB5’s impact on anti-tetanus responses was markedly less pronounced. In contrast, three consecutive daily administrations of HspB5 at these dose levels did lead to increased T-cell responses on day 28 (not shown). Over the observation period of 28 days, neither single nor multiple doses of HspB5 led to any significant change in the serum IgG antibody titers that are equally part of a normal adult human immune repertoire [9, 27].

Safety evaluation of either single or three consecutive daily intravenous administrations of HspB5 in escalating doses up to 37.5 mg showed that in all cases, HspB5 was safe and well tolerated in healthy subjects. Table 1 summarizes the treatment-emergent adverse events (AEs) that occurred in at least two subjects after a single dose of either HspB5 or placebo. More than 85% of AEs were of mild intensity, and no serious treatment-emergent AEs or deaths occurred in any of the dose groups. Upon three consecutive daily administrations, the most commonly reported drug-related AEs were injection site reactions and headache. No subjects were withdrawn due to AEs, and no clinically relevant changes were observed in vital signs, safety laboratory or 12-lead electrocardiogram (ECG) parameters in any group.

**Table 1:** Treatment-emergent adverse events occurring during the Phase I study in at least two different subjects following a single dose of HspB5.

		placebo	4 mg	12.5 mg	25 mg	37.5 mg
System organ class	Preferred term	N = 8	N = 8	N = 8	N = 8	N = 8
Gastrointestinal disorders	Abdominal pain				1 (12.5%)	1 (12.5%)
	Nausea	1 (12.5%)			1 (12.5%)	1 (12.5%)
Infections and Infestations	Nasopharyngitis		1 (12.5%)		1 (12.5%)	
	Rhinitis	1 (12.5%)	1 (12.5%)			1 (12.5%)
Nervous system disorders	(Postural) dizziness				2 (25.0%)	2 (25.0%)
	Headache	1 (12.5%)	2 (25.0%)			
Respiratory, thoracic and mediastinal disorders	Oropharyngolaryngeal pain		2 (25.0%)		1 (12.5%)	

Together, these data confirm that single intravenous doses of HspB5 between 4 and 37.5 mg are safe, well tolerated, and lead to systemic concentrations of HspB5 that remain at a sub-immunogenic level, as evidence by the lack of any increase in either T-cell or serum antibody reactivity to HspB5 following HspB5 administration.

### Phase IIa study: baseline characteristics and patient disposition

Based on the Phase I findings, a Phase IIa study was designed to examine the effects of sub-immunogenic intravenous doses of HspB5 in RR-MS patients. In this study, dose levels of 7.5, 12.5 and 17.5 mg were selected, being well within the safe, well-tolerated and sub-immunogenic dose range as defined in the Phase I study (cf. Fig 2B and 2C). In the Phase IIa study, these doses were administered to RR-MS patients three times, with 2-month intervals. The study design included repeated administration to promote tolerance for HspB5. Already for decades, intravenous administration of sub-immunogenic doses of a protein antigen has been known to be an effective strategy for antigen-specific tolerance induction [28–31]. Recent data have shown that effective tolerance induction in memory T cells *in vivo* relies not only on using sub-immunogenic intravenous doses of the target protein, but also on repeating such administration at least twice [32].

Table 2 summarizes baseline characteristics of the four treatment groups. As the result of the small group size of 8 subjects, some imbalances emerged in randomization. Patients in the 17.5-mg dose group were more recently diagnosed than in the other groups, and showed markedly more disease activity at baseline in terms of gadolinium-enhancing (Gd^+^) T1 MRI lesions than the other groups. As illustrated in the diagram of Fig 1, a total of 32 patients received intravenous placebo or HspB5 at three dose levels. All patients whose treatment was discontinued during the treatment period withdrew informed consent for different personal reasons, except for one patient in the 7.5-mg group, whose treatment was discontinued because of a major protocol violation, *i*.*e*. the use of an excluded MS drug. One patient in the placebo group and two in the 7.5-mg group withdrew consent during the follow-up period.

**Table 2:** Phase IIa study baseline characteristics.

	placebo	7.5 mg	12.5 mg	17.5 mg
Number	8	8	8	8
Female/male	3/5	6/2	5/3	5/3
Age (years; mean ± SD)	41.9 ± 8.8	39.4 ± 9.2	34.4 ± 7.5	38.1 ± 12.2
Age range (years)	31–54	27–54	23–45	22–56
Weight (kg; mean ± SD)	69.9 ± 13.6	67.8 ± 17.9	58.5 ± 15.0	68.4 ± 16.6
BMI (kg/m^2^ mean ± SD;)	23.1 ± 3.1	22.9 ± 4.3	20.5 ± 3.5	25.6 ± 5.5
Time since diagnosis (yrs; mean ± SD)	9.7 ± 6.2	6.7 ± 5.8	6.11 ± 6.4	2.1 ± 2.4
Time since last relapse (mo; mean ± SD)	3.9 ± 3.6	3.3 ± 2.2	4.2 ± 2.5	3.7 ± 3.4
Gd^+^ T1 MRI lesions (n; mean ± SD)	1.1 ± 2.4	1.1 ± 2.8	1.5 ± 2.0	2.4 ± 3.0
EDSS (mean ± SD)	2.9 ± 1.6	3.1 ± 1.6	3.8 ± 1.6	3.4 ± 1.6
EDSS ≤ 3.5 (n)	6	5	3	5
EDSS 4–5.5 (n)	2	3	5	3
MSIS-29 (mean ± SD)	53.0 ± 14.9	58.1 ± 19.5	62.5 ± 23.0	63.3 ± 18.6

### Safety evaluation

As summarized in Table 3, 24 AEs occurred during the treatment phase in patients who received any of the three dose levels of HspB5, while 6 AEs occurred in the placebo group. One of the AEs, a brief circulatory collapse in a female 17 h after receiving 7.5 mg HspB5 was considered by the investigator to be related to treatment. During the follow-up period, five AEs were reported in HspB5-treated patients, and one in the placebo group, none of which were considered to be related to HspB5 treatment. Five HspB5-treated patients reported serious AEs as compared to one in the placebo group. None of the serious AEs were considered to be related to HspB5 administration. One patient in the 12.5-mg group and one in the 17.5-mg group reported a serious AE during the follow-up period. The most commonly reported AEs included the occurrence of an MS relapse, asthenia, influenza-like illness and viral infection. None of the patients withdrew because of an AE. In addition, no clinically relevant changes were recorded in hematological parameters, vital signs or ECG parameters. In conclusion, three bimonthly intravenous administration of HspB5 up to 17.5 mg was found to be safe and well tolerated in MS patients.

**Table 3:** Treatment-emergent adverse events occurring during the Phase IIa study in RR-MS patients.

		placebo	7.5 mg	12.5 mg	17.5 mg
System organ class	Preferred term	N = 8	N = 8	N = 8	N = 8
**TREATMENT PERIOD**					
Ear and labyrinth disorders	Vertigo		1 (12.5%) / 1^1^		
	Vertigo positional	1 (12.5%) / 1			1 (12.5%) / 1
Gastrointestinal disorders	Tooth loss				1 (12.5%) / 1
General disorders and administration site reactions	Asthenia		1 (12.5%) / 2	2 (25.0%) / 2	
	Influenza-like illness	1 (12.5%) / 1	1 (12.5%) / 1		1 (12.5%) / 1
Infections and infestations	Tooth abcess				1 (12.5%) / 1
	Viral infection		1 (12.5%) / 1		1 (12.5%) / 1
Injury, poisoning and procedural complications	Contrast media reaction	1 (12.5%) / 2			
	Joint dislocation				1 (12.5%) / 1
Investigations	ECG QRS complex prolonged				1 (12.5%) / 1
Nervous system disorders	MS relapse	1 (12.5%) / 1	1 (12.5%) / 1	1 (12.5%) / 1	2 (12.5%) / 3
Psychiatric disorders	Depression	1 (12.5%) / 1		1 (12.5%) / 1	
Renal and urinary disorders	Tubulointerstitial nephritis				1 (12.5%) / 1
Respiratory, thoracic and mediastinal disorders	Oropharyngeal pain		1 (12.5%) / 1		
Vascular disorders	Circulatory collapse		1 (12.5%) / 1		
	Hypertension				1 (12.5%) / 1
	Thromboangiitis obliterans		1 (12.5%) / 1		
**FOLLOW-UP PERIOD**					
Ear and labyrinth disorders	Vertigo			1 (12.5%) / 1	
	Vertigo positional	1 (12.5%) / 1			
General disorders and administration site reactions	Influenza-like illness		1 (12.5%) / 1		
Nervous system disorders	MS relapse		1 (12.5%) / 1		
Respiratory, thoracic and mediastinal disorders	Oropharyngeal pain				1 (12.5%) / 1
Musculoskeletal and connective tissue disorders	Arthritis				1 (12.5%) / 1
Reproductive system and breast disorders	Metrorrhagia				1 (12.5%) / 1

^1^ Table 3 lists the number of patients with a particular AE, (the group percentage this represents) / the number of such AEs recorded.

### Pharmacokinetic evaluation

The pharmacokinetic profile of HspB5 following intravenous administration in RR-MS patients was very similar to that previously found in healthy subjects. Maximum serum concentrations of HspB5 were reached between 10 and 20 min after administration, and they were dose proportional at a mean ± SD of 2.49 ± 1.22 μg/mL, 4.26 ± 0.96 μg/mL, and 5.88 ± 0.93 μg/mL, respectively. Again, these concentrations are all well within the sub-immunogenic range as illustrated in Fig 2B and 2C. Serum concentration half-lives in RR-MS patients were between 56 and 128 minutes.

### Immunological parameters

In none of the groups did a significant change occur in the levels of HspB5-reactive serum IgG antibodies over the full treatment and follow-up period of 48 weeks, in line with Phase I findings and confirming the doses being sub-immunogenic. Evaluation of antigen-specific proliferative responses by either CD4^+^ or CD45RO^+^ memory T cells was unfortunately hampered by excessive background responses in all groups. During development as well as during implementation of the CFSE proliferation assay in the Phase I study, the fraction of spontaneously emerging CFSE^dim^ T cells after 9 days of culture generally remained well below a level of 1% for both CD4^+^ and CD45RO^+^ memory T cells. In the Phase IIa study, however, such background fractions of proliferated CFSE^dim^ T cells recorded in the local laboratory selected to perform the analysis were consistently between 5 to 10%, while the presence of either tetanus toxoid or HspB5 as a stimulatory antigen led to only marginal, non-significant increases in these fractions. The cause of this excessive background response which prevented reliable quantitation of antigen-specific T-cell responses could not be identified or eliminated.

### MRI parameters

**Fig 4:**
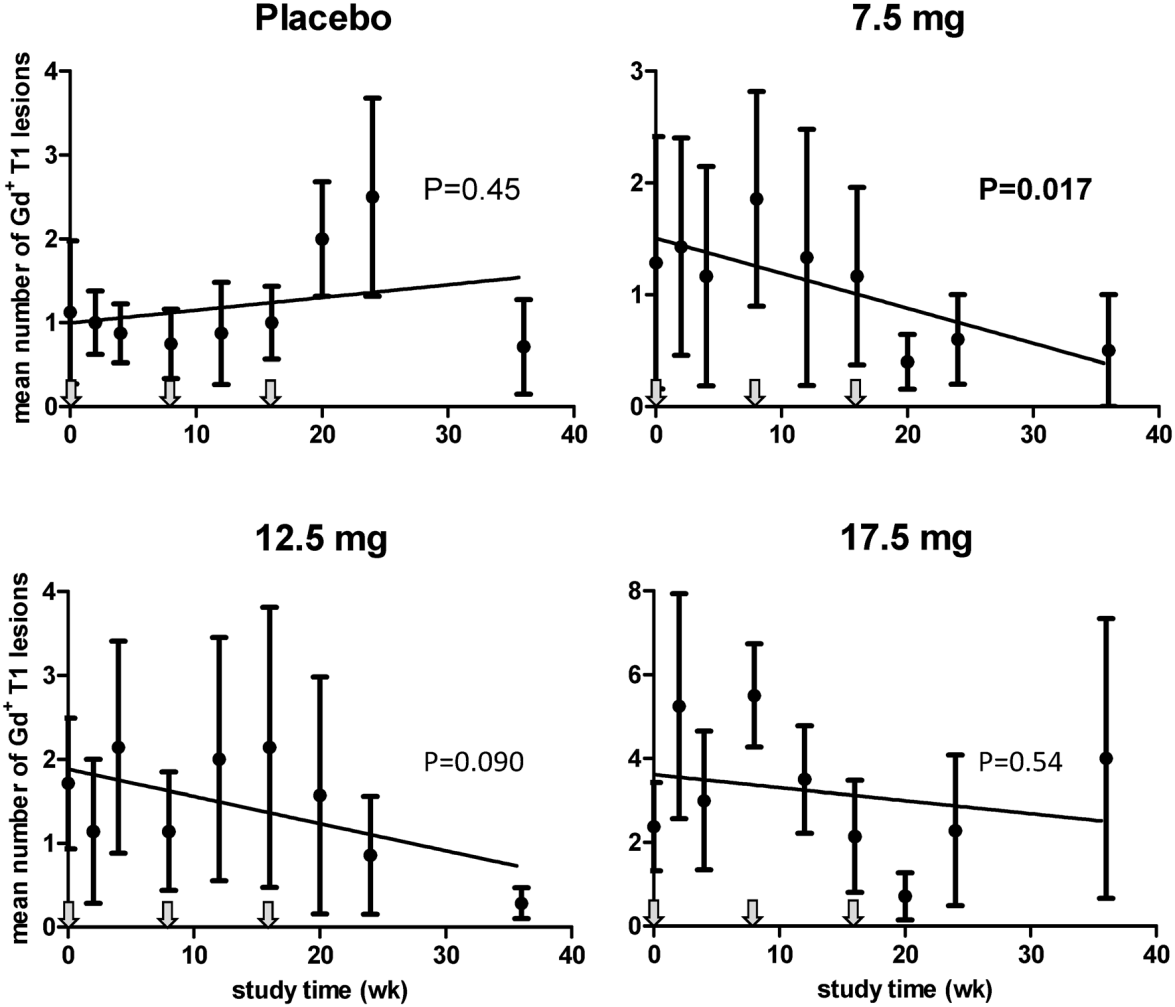
The impact of intravenous HspB5 on active MRI lesions in RR-MS patients. RR-MS patients received three bimonthly intravenous administrations of either PBS as placebo, or HspB5 at the indicated doses, and mean numbers of Gd^+^ T1 lesions for each group were evaluated by MRI over 36 weeks. Fig 4 shows mean values ± standard deviation, as calculated for the full analysis set of patients, including readings for 7 to 8 subjects at different time points for each group except for the 7.5-mg group, which included readings for 4 to 8 subjects. Linear regression analysis was used to evaluate the trends of change in each group over 36 weeks; p values reflect the probability that the deviation of such trends from a horizontal line is a chance event. Arrows indicate times of administration of placebo or HspB5.

Table 4 lists the cumulative number of newly-emerging active Gd^+^ T1 MRI lesions over the treatment and follow-up period for each treatment group. This predefined MRI end point did not significantly differ between groups. However, cumulative numbers of lesions found as of week 4 is obviously a less suitable measure to quantify clinical efficacy when disease activity changes only gradually, which appeared to be the case in the present study. Especially in subjects receiving the two lower doses of HspB5, mean numbers and total volumes of all active Gd^+^ T1 lesions, as well as of newly-emerging lesions, tended to decline relative to baseline only after repeated HspB5 administration. To evaluate these progressive changes in MRI lesion load, we performed a *post-hoc* exploratory evaluation of MRI data for all study groups using linear regression analysis as a simplified approximation of such changes. This analysis revealed a progressive decline in active MRI lesions particularly in subjects treated with either of the two lower doses of HspB5, which was not observed in the placebo group. The reduction in MRI lesions load was maximal at week 36. Fig 4 illustrates the trends of change in the mean numbers of Gd^+^ T1 lesions over 36 weeks for all treatment groups (placebo group: p = 0.45; 7.5-mg group: p = 0.017; 12.5-mg group p = 0.090). In the 17.5-mg dose group, the decline in lesion load is much less clear (p = 0.54), but it should be noted that mean data for this group are strongly influenced by the data for a single individual with very high disease activity, whose MRI scans at times revealed up to 24 Gd^+^ T1 lesions. Data for the other 7 patients in the 17.5-mg group were much more in line with the trends observed in the low-dose groups than the mean data in Fig 4 suggest.

**Table 4:** Cumulative numbers of newly-emerging Gd^+^ T1 MRI lesions during treatment and follow-up periods.

	placebo	7.5 mg	12.5 mg	17.5 mg
Over week 4 to 24 (mean ± SD)	5.6 ± 6.6	4.1 ± 6.9	6.0 ± 12.0	10.8 ± 12.5
Over week 4 to 48 (mean ± SD)	8.5 ± 8.9	4.9 ± 7.0	7.6 ± 15.6	14.9 ± 21.3

**Fig 5:**
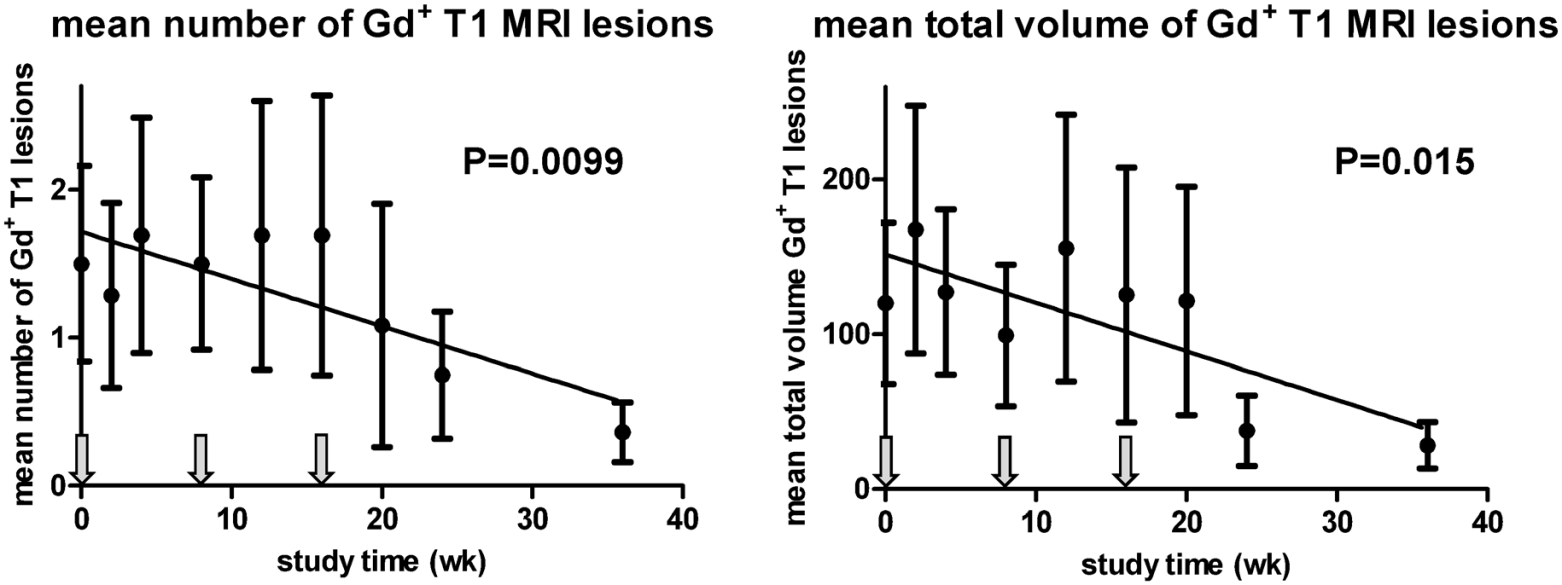
Decline in MRI lesions RR-MS patients treated with the two lower doses of HspB5. RR-MS patients received three bimonthly intravenous administrations of either 7.5 or 12.5 mg HspB5, and mean numbers as well as total volumes of Gd^+^ T1 lesions for the combined groups were evaluated by MRI over the course of 36 weeks. Data show mean values ± standard deviation, as calculated for the full analysis set of patients, including readings for 11 to 14 subjects at different time points. Linear regression analysis was used to evaluate the trends of change over 36 weeks; p values reflect the probability that the deviation of such trends from a horizontal line is a chance event. Arrows indicate times of administration of HspB5.

To more clearly illustrate the progressive suppressive effect on MRI lesion load by HspB5 treatment, Fig 5 shows combined data for the two low-dose groups over 36 weeks, illustrating a statistically significant HspB5-induced decline both in the mean numbers of Gd^+^ T1 lesions (p = 0.0099) as well as their total volumes (p = 0.015) over 36 weeks. At week 36, both mean number and total volumes of Gd^+^ T1 lesions were reduced as compared to baseline by 76%, a change that approached statistical significance (p = 0.060 in both cases, as assessed by paired Student’s t-test). Mean numbers and volumes of Gd^+^ T1 lesions recorded after 48 weeks were increased again relative to week 36, approaching the levels seen at baseline (not shown).

### Clinical parameters

No significant changes from baseline were found in any of the treatment groups with regard to the Kurtzke Expanded Disability Status Scale (EDSS) or MS Impact Scale (MSIS)-29 quality of life scores. A total of 8 clinical relapses occurred in 6 patients during the study, seven of which led to hospitalization. One relapse of mild intensity occurred in the 7.5-mg and one in 12.5-mg groups. In the 17.5-mg group, three relapses occurred; one mild, one moderate and one severe. All of these relapses resolved without sequelae, as opposed to the relapse occurring in the placebo group which was of moderate intensity and resolved with sequelae. Although the small number of relapses found in HspB5-treated patients do not allow for any firm conclusions to be drawn on relapse rates, it is of interest to note that the frequency of relapses in the HspB5-treated groups decreased over time, in line with the decline of disease activity as indicated by the MRI data. In the four consecutive 3-month intervals of the study, the numbers of relapses in all HspB5-treated patients were 4, 2, 1, and 0, respectively.

## Discussion

While progress has been made in reducing relapse rates and slowing down disease progression in MS, several important challenges remain. Of particular concern is the long-term safety of current MS drugs that obstruct adequate functioning of large subpopulations of human immune cells by hampering their normal trafficking, or even by completely eliminating them. Evidence has accumulated that a normal functioning adaptive immune system is critical for a normal functioning CNS, as well as for restoration of homeostasis following CNS injury [33]. Such considerations underpin the notion that more selective therapy in MS, for example by selectively targeting only pathogenic T cells while not affecting the vast majority of immune cells, may offer an attractive alternative. As previously explained by us in detail [34], ample evidence suggests that the small heat shock protein HspB5 plays a pivotal role in lesion development during MS. Given its high levels of expression within an MS-affected CNS and its ability to trigger IFN-γ release by the natural repertoire of HspB5-reactive human memory T cells, HspB5 is likely to be an important driver of pathogenic T-cell responses within the CNS during MS. It is of interest to note that like MS itself, the existence of a substantial pool of HspB5-reactive memory T cells is a trait unique to humans, and associated with Epstein-Barr virus infection [35].

In several animal models for neuroinflammation, intraperitoneal or intravenous administration of HspB5 has been found to exert powerful therapeutic effects due to its beneficial chaperone functions as well as its ability to trigger protective TLR2-mediated responses in microglia, monocytes and macrophages [10–17]. At the same time, intravenous administration of protein antigens is a time-honored method to induce tolerance at the level of conventional peripheral T-cell responses. As well as in other autoimmune models, its application in the animal model for MS has been well documented [28, 29]. A large number of studies on the subject have clarified that as a rule, low sub-immunogenic doses of intravenous antigen lead to tolerance by activating peripheral antigen-specific regulatory T cells [30, 31]. As explained in the introduction, the TLR2-mediated human macrophage response to HspB5, inducing factors such as PD-1 ligand, the TIGIT ligand CD155, IL-10, indoleamine-2,3-dioxygenase-1, tumor necrosis factor-α-stimulated gene 6 and TGF-β, actively supports such tolerance induction [21]. More recent data have highlighted that in order to induce functional tolerance in memory T cells, at least two intravenous doses of antigen are required [32]. Once induced, peripheral human regulatory T cells are likely to be limited in their actions by a finite life span of several months [36].

When considering the outcome of the present Phase IIa study, apart from the intravenous route of HspB5 administration, the use of sub-immunogenic dose levels, and the active induction of tolerogenic macrophage factors by HspB5, also the temporal profile of clinical responses to treatment in MS patients is therefore consistent with tolerance induction via activation of peripheral regulatory T cells. The reduction in MRI lesion load in MS patients required several HspB5 administrations to become apparent, and subsided again about 4–5 months after the last administration of HspB5. Based on these considerations and our Phase I data as illustrated in Fig 3, and despite the inconclusive outcome of CFSE assays performed during the Phase IIa study, we favor the idea that tolerance induction via antigen-specific regulatory T cells is an important contributing factor to the clinical effects of intravenous HspB5 in RR-MS patients.

While the small sample size and limited duration of the present study in RR-MS patients preclude definitive conclusions with regard to clinical efficacy of HspB5 treatment, the current MRI data demonstrate progressively suppressive effects on lesion development in patients. These effects become apparent after several intravenous administrations of the lower doses of HspB5, and they are not seen in the placebo group. Along with the favorably safety profile, these data encourage further exploration of the therapeutic use of HspB5 in MS. As explained in the introduction, HspB5 also exerts direct and broad neuroprotective and regenerative effects in several animal models of neuroinflammation. By combining selective suppression of pathogenic T cells with such complementary neuroprotective effects, HspB5 may well offer benefit in the management not only of relapsing, but also progressive forms of MS.

## Supporting Information

S1 CONSORT Checklist CONSORT 2010 checklist of information to include when reporting a randomised trial. (DOCX)

S1 Protocol Clinical study protocol. A Phase I, randomized, double-blind, placebo-controlled study to evaluate the safety, tolerability, pharmacokinetics and T-cell tolerizing effect of DC-TAB in healthy volunteers.
(DOCX)

S2 Protocol Clinical study protocol. A Phase IIa, randomized, double-blind, placebo-controlled, exploratory, dose-ranging study to evaluate the safety, effectiveness and pharmacokinetics of three courses of DC-TAB treatment in patients with multiple sclerosis.
(DOCX)
