# Supplementary material for: Therapeutic Intervention in Multiple Sclerosis with Alpha B-Crystallin: A Randomized Controlled Phase IIa Trial
Source: PLoS One. 2015 Nov 23;10(11):e0143366. doi: 10.1371/journal.pone.0143366 (PMC4657879; doi:10.1371/journal.pone.0143366)
Supplement: S1 Protocol — A Phase I, randomized, double-blind, placebo-controlled study to evaluate the safety, tolerability, pharmacokinetics and T-cell tolerizing effect of DC-TAB in healthy volunteers. (DOCX) [file pone.0143366.s002.docx]

*A Phase I,* ***randomized, double-blind, placebo-controlled*** *study to evaluate the safety, tolerability, pharmacokinetics and T-cell tolerizing effect of DC-TAB in healthy volunteers*

| **Study no.** | DC-001 |
| --- | --- |
| **EudraCT no.** | 2009-016817-68 |
| **Investigational product** | DC-TAB |
| **Indication** | Multiple sclerosis |
| **Development phase** | I |
| **Date of protocol** | 4 November 2009 |
| **Version of protocol** | Version 2, final |
| **Principal investigator** | Dr. Floris Höppener, M.D.  Kendle International  Bolognalaan 40  3584 CJ Utrecht  The Netherlands |
| **Sponsor** | Delta Crystallon BV  p/a Zernikedreef 9  2333 CK Leiden  The Netherlands  Tel +31 71 518 1541  Fax +31 71 518 1901 |
| This study will be performed in compliance with Good Clinical Practices (ICH-GCP), including the archiving of essential documents. | |
| **This document is property of Delta Crystallon BV and contains confidential information. It may not be forwarded to third parties without explicit written prior consent from Delta Crystallon BV. Reproductions, either in part or in whole, may not be published or copied in any manner, without the explicit written consent of Delta Crystallon BV.** | |

# Study synopsis

| **Title of Study**  A Phase I, randomized, double-blind, placebo-controlled study to evaluate the safety, tolerability, pharmacokinetics and T-cell tolerizing effect of DC-TAB in healthy volunteers. | |
| --- | --- |
| **Investigator(s)**  Dr. Floris Höppener, Kendle International B.V., Bolognalaan 40, 3584 CJ Utrecht, the Netherlands | |
| **Number of sites**  1 | |
| **Study period**  Planned study period: December 2009 – April 2010 | **Phase of development**  I |
| **Objectives**  Primary objective  To evaluate the safety and tolerability of DC-TAB following a single dose and following repeated dosing in healthy volunteers.  Secondary objectives  To assess the pharmacokinetics of DC-TAB following a single dose and following repeated dosing in healthy volunteers.  To evaluate the T-cell tolerizing effect of DC-TAB in healthy volunteers.  To evaluate levels of DC-TAB specific and -neutralizing antibodies. | |
| **Methodology**  This study is a double-blind, randomized, placebo-controlled, dose-escalation study. The study will consist of 2 parts. In Part 1, subjects will receive a single dose of study medication whereas in Part 2, (different) subjects will receive study medication on 3 consecutive days.  In Part 1, four groups of subjects (n=10) will be studied in a single dose, dose-escalation design. Each group of subjects will be randomized to receive either DC-TAB (n=8) or placebo (n=2) once.  In Part 2, three groups of subjects (n=12) will be studied in a multiple dose, dose-escalation design. Each group of subjects will be randomized to receive either DC-TAB (n=9) or placebo (n=3) once daily on 3 consecutive days.  The next higher dose group in each part of the study will start once safety data up to t=4 days for Part 1 and t=8 days of Part 2 of the previous dose group have been reviewed and have raised no safety concerns.  Part 2 will start once all safety data of Part 1 have been reviewed. If necessary, the doses may be adjusted downward. | |
| **Number of subjects planned**  Total of 76 subjects: 40 (8 per dose) in Part 1 and 36 (9 per dose) in Part 2 | |
| **main criteria for inclusion**   - Healthy male or female volunteers aged 18 to 55 years (inclusive). - BMI between 20.0 and 28.0 kg/m^2^. - In general good health. - Subjects who have been informed of the nature and aims of the study and have given their written consent to participate in this study in accordance with local laws and requirements. - Subjects who are willing to comply with the protocol and understand the information given and the text of the consent form. | |
| **Test product, dose and mode of administration**  Code name: DC-TAB  Class: Recombinant protein  Concentration: 12.5 mg/mL (1.0 ml vials)  Dose levels Part 1: Single dosing; Group 1: 4 mg, Group 2: at most 12.5 mg, Group 3: at most 25 mg and Group 4: at most 37.5 mg  Dose levels Part 2: Group 1: max. 10 mg, Group 2: max. 25 mg and Group 3: max. 37.5 mg once daily for 3 consecutive days  Mode of administration: Intravenous bolus injection in the arm | |
| **Duration of treatment**  Part 1. Single dose  Part 2. 3 days | |
| **Reference therapy, dose and mode of administration, batch number**  Placebo (PBS) will be used as reference.  Placebo will be administered in exactly the same way as the active medication using the same volume. | |
| **Criteria for evaluation** | |
| **Safety parameters**   - Number and frequency of adverse events and serious adverse events - Local tolerability - ECG parameters - Vital signs (blood pressure, pulse and respiration rate) - Oral body temperature - Hematology, biochemical and urinalysis parameters - Physical examination   **Effect parameters**  Proliferative response by CRYAB-reactive peripheral blood CD4+ T-cells  Level of CRYAB-reactive serum antibodies  Neutralizing activity of CRYAB-reactive serum antibodies | |
| **Pharmacokinetics**   - AUC_0-24h_ - AUC_0-∞_ - C_max_ - t _1/2_ - t _max_ - Kel (λ_Z_) | |
| **Statistical methods**  **Statistical analysis methods**  Safety population  The safety population will comprise of all subjects who received one dose of the investigational product.  Sample size  Since this is the first study in men with DC-TAB the sample size is based on medical and practical grounds rather than statistical grounds.  Safety parameters  No formal statistics will be employed   - Adverse events (AEs) will be coded and presented using MedDRA. The overall incidence, the incidence of related as well as the incidence of serious adverse events (SAEs) will be summarized per dose and by body system in tables. - Local tolerability will be presented by overall incidence by treatment group per symptom. - Vital signs (supine blood pressure, heart rate, body temperature and respiratory rate): Descriptive statistics (n, mean, standard deviation, median, minimum, and maximum) will be provided for blood pressure, heart rate, body temperature and respiratory rate and for changes from baseline. - Hematology, biochemistry and urinalysis parameters: Descriptive statistics (n, mean, standard deviation, median, minimum, and maximum) will be provided on values at baseline and all post-dose assessments. Descriptive statistics will be calculated on the change from baseline values. Out-of-normal-range values will be indicated for each parameter. The change in out-of-normal-range values after administration and at follow up versus baseline will be summarized per group by means of shift-tables. - ECG: Descriptive statistics (frequency, mean, standard deviation, median, minimum, and maximum) on ECG parameters will be provided at baseline and at all post-baseline assessments. Changes from baseline values will be calculated and descriptive statistics will be computed. Shift tables will also be provided to indicate changes in ECG parameters over time.   PK parameters  Descriptive statistics (n, mean, standard deviation, median, minimum, and maximum, plus Geometric mean and CV for C_max_ and AUC and the derived parameters) will be provided for the PK parameters.  Evaluations of dose-proportionality and effects of multiple dosing will be performed.  Effect parameters  Descriptive statistics (n, mean, standard deviation, median, minimum, and maximum) at each visit and change from baseline (n, mean, standard deviation, median, minimum, and maximum) will be provided for all effect parameters. | |

# Sponsor’s signatures

***A Phase I, randomized, double-blind, placebo-controlled study to evaluate the safety, tolerability, pharmacokinetics and tolerizing effect of DC-TAB in healthy volunteers.***

On behalf of the sponsor Delta Crystallon BV.

p/a Zernikedreef 9

2333 CK Leiden

The Netherlands

By my signature below I agree to the terms of this study protocol

_____________________________ ____________________

E.H.G. Venneker, M.D. Date

Afforce Healthcare BV.

Author & Sponsor Medical Responsible Person

_____________________________ ____________________

J.M. van Noort, Ph.D. Date

Delta Crystallon BV

Chief Scientific Officer

# Principal Investigator signature

***A Phase I, randomized, double-blind, placebo-controlled*** ***study to evaluate the safety, tolerability, pharmacokinetics and T-cell tolerizing effect of DC-TAB in healthy volunteers.***

By my signature below I agree to conduct this clinical trial in accordance with the protocol, ICH-GCP, the Declaration of Helsinki, government regulations and national laws, including those applying to institutional/ethics review and informed consent.

______________________________ ____________________

Dr. Floris Höppener, M.D. Date

Kendle International

This document contains confidential information, which should not be copied, referred to, released or published without prior written approval from Delta Crystallon BV. Investigators are cautioned that the information in this protocol may be subject to change and revision.

# Table of Contents

[Study synopsis 2](#_Toc243714421)

[Sponsor’s signatures 5](#_Toc243714422)

[Principal Investigator signature 6](#_Toc243714423)

[Table of Contents 7](#_Toc243714424)

[List of abbreviations and definition of terms 10](#_Toc243714425)

[1 Introduction 12](#_Toc243714426)

[1.1 Background 12](#_Toc243714427)

[1.2 Investigational product 12](#_Toc243714428)

[1.3 Non-clinical data 13](#_Toc243714429)

[1.4 Clinical data 14](#_Toc243714430)

[1.5 Study rationale and risk/benefits 14](#_Toc243714431)

[2 Investigators and study administrative structure 15](#_Toc243714432)

[3 Study objectives 16](#_Toc243714433)

[3.1 Primary Objective 16](#_Toc243714434)

[3.2 Secondary Objectives 16](#_Toc243714435)

[4 Investigational plan 17](#_Toc243714436)

[4.1 Overall study design and plan – description 17](#_Toc243714437)

[4.2 Study flow chart 17](#_Toc243714438)

[4.3 Discussion of study design, including choice of control groups 18](#_Toc243714439)

[4.4 Study population 18](#_Toc243714440)

[4.4.1 Inclusion criteria 18](#_Toc243714441)

[4.4.2 Exclusion criteria 18](#_Toc243714442)

[4.4.3 Randomization criterion 20](#_Toc243714443)

[4.4.4 Excluded prior and concomitant treatments 20](#_Toc243714444)

[4.4.5 Withdrawal of subjects 20](#_Toc243714445)

[4.4.6 Pausing and premature termination of the study 20](#_Toc243714446)

[5 Treatments 22](#_Toc243714447)

[5.1 Treatments administered 22](#_Toc243714448)

[5.2 Selection of doses in the study 22](#_Toc243714449)

[5.3 Methods of assigning subjects to treatment groups 22](#_Toc243714450)

[5.4 Blinding procedures 22](#_Toc243714451)

[5.5 Treatment compliance 23](#_Toc243714452)

[6 Conduct of the study 24](#_Toc243714453)

[6.1 Screening and selection of subjects 24](#_Toc243714454)

[6.2 Baseline periods 24](#_Toc243714455)

[6.3 Treatment & follow-up period 24](#_Toc243714456)

[6.4 Final study visit 25](#_Toc243714457)

[6.5 Early Termination Visit 25](#_Toc243714458)

[6.6 Schedule of assessments 26](#_Toc243714459)

[6.7 Compliance with the protocol 27](#_Toc243714460)

[7 Methodology of assessments 28](#_Toc243714461)

[7.1 Baseline variables 28](#_Toc243714462)

[7.1.1 Demographics 28](#_Toc243714463)

[7.1.2 Medical history 28](#_Toc243714464)

[7.1.3 Previous Medication 28](#_Toc243714465)

[7.2 Safety variables 28](#_Toc243714466)

[7.2.1 Laboratory Parameters 28](#_Toc243714467)

[7.2.2 Physical Examination 29](#_Toc243714468)

[7.2.3 Vital Signs 29](#_Toc243714469)

[7.2.4 Body temperature 29](#_Toc243714470)

[7.2.5 12-Lead Electrocardiogram 29](#_Toc243714471)

[7.2.6 Continuous cardiac monitoring 29](#_Toc243714472)

[7.2.7 Adverse Events including Local and Systemic Reactions 29](#_Toc243714473)

[7.2.8 Concomitant medication/antidotes 30](#_Toc243714474)

[7.3 Pharmacokinetics 30](#_Toc243714475)

[7.4 Effect parameters 30](#_Toc243714476)

[8 Study medication 31](#_Toc243714477)

[8.1 Identity of the investigational products 31](#_Toc243714478)

[8.2 Drug manufacturing 31](#_Toc243714479)

[8.3 Drug storage and stability 31](#_Toc243714480)

[8.4 Preparation, administration and dosage of treatment 31](#_Toc243714481)

[8.5 Drug accountability 32](#_Toc243714482)

[9 Data handling and record keeping 33](#_Toc243714483)

[9.1 Data collection 33](#_Toc243714484)

[9.2 Data management 34](#_Toc243714485)

[9.3 Record keeping 34](#_Toc243714486)

[10 Statistical analysis and reporting 35](#_Toc243714487)

[10.1 Study parameters 35](#_Toc243714488)

[10.1.1 Safety parameters 35](#_Toc243714489)

[10.1.2 Effect parameters 35](#_Toc243714490)

[10.1.3 Pharmacokinetic parameters 35](#_Toc243714491)

[10.2 Evaluability of data 35](#_Toc243714492)

[10.3 Statistical analysis 35](#_Toc243714493)

[10.3.1 Handling of Missing and Incomplete Data 35](#_Toc243714494)

[10.3.2 Safety data 35](#_Toc243714495)

[10.3.3 Pharmacokinetic data 36](#_Toc243714496)

[10.3.4 Effect data 36](#_Toc243714497)

[10.3.5 Demographic and background variables 36](#_Toc243714498)

[10.3.6 Subject accountability 36](#_Toc243714499)

[10.3.7 Study medication compliance 36](#_Toc243714500)

[10.4 Sample size justification 37](#_Toc243714501)

[10.5 Study report 37](#_Toc243714502)

[11 Adverse events 38](#_Toc243714503)

[11.1 Adverse event definition 38](#_Toc243714504)

[11.2 Reporting adverse events 38](#_Toc243714505)

[11.3 Reporting of Pregnancy 39](#_Toc243714506)

[11.4 Follow-up of adverse events 39](#_Toc243714507)

[12 Serious adverse events 40](#_Toc243714508)

[12.1 Serious adverse event definition 40](#_Toc243714509)

[12.1.1 Unexpected adverse drug reaction 40](#_Toc243714510)

[12.1.2 Life-threatening adverse event 40](#_Toc243714511)

[12.1.3 Hospitalization 40](#_Toc243714512)

[12.1.4 Persistent or significant disability/incapacity 41](#_Toc243714513)

[12.1.5 Medical occurrence requiring intervention to prevent permanent impairment or damage 41](#_Toc243714514)

[12.2 Reporting serious adverse events and/or unexpected adverse drug reactions 41](#_Toc243714515)

[12.3 Suspected Unexpected Serious Adverse Reaction (SUSAR) reporting 41](#_Toc243714516)

[13 Ethics and protection of study participants 42](#_Toc243714517)

[13.1 Basic principles 42](#_Toc243714518)

[13.2 Independent Ethics Committee/Institutional Review Board approval 42](#_Toc243714519)

[13.3 Regulatory requirements 42](#_Toc243714520)

[13.4 Insurance of the subject 42](#_Toc243714521)

[13.5 Informed consent 42](#_Toc243714522)

[14 Study administrative procedures 44](#_Toc243714523)

[14.1 Protocol amendments 44](#_Toc243714524)

[14.2 Study monitoring 44](#_Toc243714525)

[14.3 Subject confidentiality 44](#_Toc243714526)

[14.4 Use of information and publications 45](#_Toc243714527)

[14.5 Quality assurance 45](#_Toc243714528)

[14.6 Regulatory inspections 45](#_Toc243714529)

[15 Study documentation and supplies 46](#_Toc243714530)

[16 Reference list 47](#_Toc243714531)

# List of abbreviations and definition of terms

Abbreviations

| AE | Adverse Event/Adverse Experience |
| --- | --- |
| AUC | Area Under the Curve |
| BLLQ | Below the Lower Limit of Quantification |
| BMI | Body Mass Index |
| BUN | Blood Urea Nitrogen |
| CA | Competent Authorities |
| CI | Confidence Interval |
| CIOMS | Council for International Organizations of Medical Sciences |
| C_max_ | Maximum Concentration |
| CRF | Case Report Form |
| CRO | Contract Research Organization |
| CRYAB | Alpha B-crystallline (used to refer to endogenous occuring alpha B-crystalline) |
| CV | Coefficient of Variation |
| DBP | Diastolic Blood Pressure |
| DC-TAB | Delta Crystallon Therapeutic Alpha B-crystalline (used to refer to the IMP) |
| EOT | End Of Trial |
| FDA | Food and Drug Administration |
| GCP | Good Clinical Practice |
| GMP | Good Manufacturing Practice |
| GLP | Good Laboratory Practice |
| Hb | Haemoglobin |
| HBsAg | Hepatitis B Surface Antigen |
| HCV | Hepatitis C Virus |
| HCT | Haematocrit |
| HED | Human Equivalent Dose |
| HIV | Human Immunodeficiency Virus |
| IB | Investigator’s Brochure |
| ICF | Informed Consent Form |
| ICH | International Conference on Harmonization |
| IEC | Independent or Institutional Ethics Committee |
| Kel | Elimination Rate Constant |
| LLQ | Lower Limit of Quantification |
| MedDRA © | Medical Dictionary for Regulatory Activities |
| MS | Multiple Sclerosis |
| MTD | maximum tolerated dose |
| n | Number (typically refers to subjects) |
| OTC | Over-the-Counter |
| PI | Principal Investigator |
| PK | Pharmacokinetics |
| QA | Quality Assurance |
| QC | Quality Control |
| SAE | Serious Adverse Event/Serious Adverse Experience |
| SBP | Systolic Blood Pressure |
| SOP | Standard Operating Procedure |
| t_1/2_ | Elimination half-life |
| t_max_ | Time to Reach Maximum Plasma Concentration |
| WHO | World Health Organization |
|  |  |

# Introduction

## Background

In the central nervous system (CNS) of Multiple Sclerosis (MS) patients, alpha B-crystallin accumulates in myelin membranes as a reaction to an unknown neurodegenerative process. Alpha B-crystallin accumulation and secretion as a soluble factor in the CNS is neuroprotective in itself [Ousman *et al.*, 2007]. As an intracellular heat shock protein, alpha B-crystallin protects local cells from apoptosis. As a secreted protein, it activates local microglia to secrete factors which activate the blood-brain barrier, and promote non-specific recruitment of monocytes and memory T-cells from peripheral blood for reparative immune surveillance [Bhat *et al*, 1999; van Noort *et al*, in preparation]. In all adult humans, however, the immune system contains autoreactive pro-inflammatory T-cells as well as antibodies against alpha B-crystallin [van Noort *et al*, 1995, 2006, in preparation]. Recruitment especially of these T-cells into a CNS that contains high levels of alpha B-crystallin could therefore create a potentially explosive mixture. When local accumulation of alpha B-crystallin-reactive T-cells and alpha B-crystallin itself reaches a critical threshold, an inflammatory response is likely to develop. By eliminating alpha B-crystallin-reactive T-cells, therefore, the major source of fuel which drives the MS lesion should be eliminated.

Alpha B-crystallin is secreted by CNS glial cells under conditions of damage or insults, and, as a soluble extracellular protein, alpha B-crystallin has so-called chaperokine activity. It activates a certain receptor complex on macrophage-like cells, including microglia in the CNS [Bhat *et al*, 1999; van Noort *et al*, in preparation]. This activation leads to what is generally referred to as “alternative activation” of macrophages, which promotes resolution of inflammation and repair.

As evidenced by data from different animal models of inflammatory disorders [Ousman *et al*, 2007; Masilamoni *et al*, 2005a, 2005b and 22006], systemic administration of a recombinant alpha B-crystallin promotes rapid reversal of inflammatory damage in various organs. These data indicate that DC-TAB may be effective as an anti-inflammatory compound for several other inflammatory disorders, beyond application in MS or CNS disorders only.

## Investigational product

The primary aim of the clinical studies is to test a novel strategy for the treatment of MS, a chronic inflammatory disorder of the CNS [Frohman *et al,* 2006, McFarland and Martin, 2007]. This strategy is based on the notion that the inflammatory process, which causes the destructive lesions in MS, is driven by a local autoreactive T-cell response against the small heat shock protein alpha B-crystallin [van Noort *et al*, 1995]. In the CNS of MS patients, alpha B-crystallin repeatedly accumulates at high levels in myelin [van Noort *et al*, 1995; Bajramovic *et al*, 1997 and 2000; Sinclair *et al*, 2005; Chabas *et al*, 2001; Tajouri *et al*, 2003]. While the cause for this accumulation remains to be established, the locally high levels of alpha B-crystallin thus achieved provoke recruitment of lymphocytes and, consequently, a local T-cell response against alpha B-crystallin [van Noort *et al*, in preparation]. The rationale for the development is to administer DC-TAB, a recombinant alpha B-crystallin prepared using E. coli, intravenously to induce immunological tolerance at the level of T-cells, thus taking away the fuel which drives the local inflammatory reactions in MS.

Based on animal model data [Verbeek *et al*., 2007], intravenous tolerization by three consecutive daily injections of soluble DC-TAB is expected to be rapid and highly selective, eliminating only alpha B-crystallin-reactive T-cells but no other. This intervention is expected to prevent any subsequent specific immune response to alpha B-crystallin, and halt any further progression of MS. It is not expected that the treatment will promote repair of any pre-existing damage. Natural peripheral T-cell reactivity against alpha B-crystallin is expected to gradually return over periods of years, dependent on the age of the person involved. For this reason, it is anticipated that the tolerance-inducing treatment will have to be repeated periodically for life, to secure a permanent and sufficient state of tolerance.

## Non-clinical data

The NOAEL observed in rats following 28 days dosing was set at 20 mg/kg, the highest dose tested in toxicology studies. In all animals, a significant increase was observed in serum protein, serum globulin, and in spleen size and weight, consistent with the development of an antibody response against DC-TAB.

A study was conducted in beagle dogs who received DC-TAB in doses up to 5 mg/kg. After approximately 8-10 consecutive days of intravenous DC-TAB administration, treatment-related clinical symptoms started to develop immediately after administration in all treatment groups except for the male dogs receiving 0.5 mg/kg which all remained symptom-free. Clinical symptoms included pallor of otherwise well-perfused tissues such as the tongue and membranes in eyes and mouth, often preceded by brief reddening. More severe clinical signs included lethargy, spasms, abnormal posture and gait, and abnormal breathing. In a few cases, mild secretory symptoms or pallor already developed after 5-6 days. All the above clinical symptoms reversed within minutes after DC-TAB administration. An only weak correlation was observed between the severity of clinical signs and the DC-TAB dose administered. In line with the findings in the above range-finding and maximum tolerated dose (MTD) studies, females tended to develop slightly more severe signs than males. In a rather abrupt reversal, all of these clinical signs stopped appearing after 12-14 days of treatment in almost all cases.

All pre-clinical data in dogs, the more sensitive species, indicate that there is a crucial difference between DC-TAB administration in naïve animals as compared to animals that had received DC-TAB before. Upon repeated administration, and with a minimum time interval of five days between a first and consecutive dose(s), significant clinical symptoms emerge immediately upon administration of such a consecutive dose, even a low dose. In naïve animals on the other hand, up to five consecutive doses of 12 mg/kg DC-TAB did not trigger any adverse effects.

The data do not allow formal definition of a NOAEL in female dogs following 15 days administration. In male dogs, the NOAEL is 0.5 mg/kg following 15 days administration. In naïve female and male dogs, the NOAEL up to five consecutives doses is 12 mg/kg.

Cardiovascular, respiratory and neurobehavioral safety pharmacology studies did not reveal any toxicologically significant effect of DC-TAB administered up to 20 mg/kg on any of these body systems.

The proposed starting dose in the current study protocol takes these findings into account, as well as relevant immunological differences between dogs and humans, as explained in more detail elsewhere in this document. Based on all these considerations, it is proposed to use a starting dose in humans that is 100 times lower than the human equivalent dose which did not lead to any adverse effects upon 5 consecutive administrations in naïve dogs. This translates into a starting dose of 4 mg for the proposed single-dose study, or 0.066 mg/kg for a 60 kg adult.

Based on animal model data [Verbeek *et al.*, 2007] the effective dose in humans is expected to be in the range of approximately 0.2-0.4 mg/kg, or a fixed dose of approximately 12.5 - 25 mg per injection.

For more details on the pre-clinical data please refer to the IB.

## Clinical data

No clinical data have been obtained with DC-TAB to date.

## Study rationale and risk/benefits

In this Phase I study (DC-001), single dosing by an intravenous bolus injection will be evaluated, with increasing amounts of DC-TAB to reach the doses expected to be required for tolerance induction. Based on animal model data [Verbeek *et al.*, 2007] these doses are expected to be in the range of approximately 0.2-0.4 mg/kg, or a fixed dose of approximately 12.5 – 25 mg per injection.

This study is designed to explore safety and tolerability of the intervention in humans, and the effectiveness of intravenous DC-TAB in actually inducing T-cell tolerance following a single dose and following repeated dosing. Since natural anti-alpha B-crystallin autoreactivity is part of a normal human immune system, and not limited to MS patients only, tolerance induction can already be examined during a Phase I study in healthy subjects. This study will therefore also include repeated blood sampling of subjects (both before and after dosing) to evaluate T-cell reactivity to alpha B-crystallin, allowing evaluation of the effective tolerizing dose of DC-TAB to be used in subsequent clinical studies.

# Investigators and study administrative structure

Principal investigator Floris Höppener, M.D.

Kendle International

Bolognalaan 40

3584 CJ Utrecht

The Netherlands

Sponsor’s contact Dr J.M. van Noort, Ph.D.

Chief Scientific Officer

Delta Crystallon BV

Zernikedreef 9

2333 CK Leiden

The Netherlands

Tel: +31 (0)71 518 1541

Fax: + 31 (0)71 518 1901

Mobile: +31 (0)6 5394 6231

Medical responsible person E.H.G. Venneker, M.D.

Senior Partner

Afforce Healthcare BV

Veraartlaan 8

2288 GM Rijswijk

The Netherlands

Tel: +31 (0)70 710 1431

Fax: +31 (0)70 710 1437

Mobile: +31 (0)6 5155 9672

Datamanagement/biostatistics Kendle International BV

Laboratory for pharmacokinetics ALPHALYSE A/S

Unsbjergvej 4

DK-5220 Odense SØ

Denmark

Tel: +45 6310 6500

Laboratory for effect markers Delta Crystallon BV

Zernikedreef 9

2333 CK Leiden

The Netherlands

Tel : +31 (0)71 518 1541

Fax : +31 (0)71 518 1901

# Study objectives

## Primary Objective

To evaluate the safety and tolerability of DC-TAB following a single dose and following repeated dosing in healthy volunteers.

## Secondary Objectives

To assess the pharmacokinetics of DC-TAB following a single dose and following repeated dosing in healthy volunteers.

To evaluate the T-cell tolerizing effect of DC-TAB in healthy volunteers.

To evaluate levels of DC-TAB-specific and -neutralizing antibodies.

# Investigational plan

## Overall study design and plan – description

This study is a double-blind, randomized, placebo-controlled, dose-escalation study. The study consists of 2 parts. In Part 1, all subjects will receive a single dose of study medication on day 1; in Part 2, different subjects will receive medication once daily during 3 consecutive days.

In Part 1, four groups of subjects (n=10) will be studied in a dose-escalation design. Each group of subjects will be randomized to receive either DC-TAB (n=8) or placebo (n=2) once.

In Part 2, three groups of subjects (n=12) will be studied in a dose-escalation design. Each group of subjects will be randomized to receive either DC-TAB (n=9) or placebo (n=3) once daily on 3 consecutive days.

Administration of study medication for each of the groups will take place in two cohorts. The first cohort will consist of 2 subjects of whom one will receive active medication and the other placebo. The remaining subjects of each group will be dosed (n=6 and n=10, respectively) at least 4 days after last dosing of the first 2 subjects, if the first 2 subjects of a group have not shown significant unexpected events which are considered to pose a potential risk to the volunteers.

The next higher dose group in both parts of the study will start once safety data up to t=4 days for Part 1 and t=8 days for Part 2 of the previous dose group have been reviewed and have raised no safety concerns. In addition, dosing in Part 2 will start once all safety data of Part 1 have been reviewed and approval of the IEC has been obtained. If necessary, the doses may be adjusted downward.

## Study flow chart

| **Part 1**  **Single dose** |  | **Part 2**  **Once daily on 3 consecutive days** |
| --- | --- | --- |

|  |  |  | 37.5 mg (n=8) |  |  |  | 37.5 mg (n=9) |
| --- | --- | --- | --- | --- | --- | --- | --- |
|  |  | 25 mg (n=8) |  |  |  | 25 mg (n=9) |  |
|  | 12.5 mg (n=8) |  |  |  | 10 mg (n=9) |  |  |
| 4 mg (n=8) |  |  |  |  |  |  |  |
| Plac (n=2) | Plac (n=2) | Plac (n=2) | Plac (n=2) |  | Plac (n=3) | Plac (n=3) | Plac (n=3) |
| **Group 1** | **Group 2** | **Group 3** | **Group 4** |  | **Group 5** | **Group 6** | **Group 7** |

## Discussion of study design, including choice of control groups

As this is the first study in man, a single dose will be given in Part 1 to limit the risks for subjects. In order to further limit any potential risk, subjects in Part 1 will be dosed in two cohorts (2 and 8 subjects, respectively). As a result, serious unexpected side effects such as e.g. anaphylactic reactions will likely be detected before any additional subjects are exposed to the IMP.

As repeated dosing in Part 2 will take place after all subjects of Part 1 have received a single dose, safety data following a single dose will be available from doses up to 37.5 mg. The dosing schedule in Part 2 will be similar to Part 1 with subjects dosed in 2 cohorts of 2 and 10 subjects, respectively. Once safety data obtained for at least 4 days following the last dose have revealed no safety concerns, the next higher dose group will receive study medication.

Inclusion of a placebo control group will allow for a minimum of bias and correct for possible placebo effects. As this is a phase I study in healthy volunteers, the use of a placebo control group is considered ethical.

As this study is not designed to determine efficacy, the number of subjects per treatment group for this study is not based on statistical considerations, but based on medical/scientific and practical grounds.

## Study population

76 male and female healthy subjects between 18 and 55 years of age.

### Inclusion criteria

1. Caucasian
2. Signed the written informed consent form before first screening procedure
3. Age ≥ 18 years and ≤ 55 years
4. In general good health in the opinion of the investigator
5. BMI between 20.0 and 28.0 kg/m^2^
6. Use of adequate and stable contraception for 3 months prior to study initiation, during the course of the study and 30 days thereafter. Sexually active males must use a condom. Sexually active females must use double-barrier contraception or hormonal contraceptive (oral, transdermal, vaginal ring, implants), or must have undergone clinically documented total hysterectomy and/or oophorectomy, surgical sterilization or be postmenopausal defined by amenorrhea for at least 12 months and confirmed with a FSH ≥ 40 mIU/ml.
7. If subjects claim abstinence as their method of contraception, they must be willing to agree to use condoms if they become sexually active from 14 days prior to the first dose of the study drug through 90 days beyond the conclusion of the study.

### Exclusion criteria

1. Pregnant women, women planning to become pregnant and breastfeeding women
2. Subjects with a history of MS in first grade family members
3. A history of or currently active clinically significant cardiac (including clinically significant ECG abnormalities in the opinion of the PI), pulmonary, gastrointestinal, hepatic, renal, pancreatic, or neurological disease
4. ALT, AST and/or gamma-GT above 3 times the upper limit of normal
5. Serum creatinine above 1.5 times the upper limit of normal
6. Amylase above 1.5 times the upper limit of normal
7. Hemoglobin < 7.0 mmol/l for females and < 8 mmol/l for males; leucocytes > 20*10^9^/l or < 3.5*10^9^/l; platelets < 125*10^9^/l
8. SBP > 160 mmHg and/or DBP > 100 mmHg
9. Known or suspected hypersensitivity to any component of DC-TAB
10. Known or suspected impairment of the immune system
11. Acute respiratory or other active infections or illnesses
12. Fever (oral temperature > 38.0 °C on day 1)
13. Blood donation or significant blood loss within 90 days of first study medication dosing.
14. Plasma donation within 7 days of first study medication dosing
15. Recipients of blood or blood products in the last 6 months
16. Participation in another clinical study within 90 days of the start of this trial or planning participation in another clinical trial during this study or in the 4 weeks after last visit
17. Taking immunosuppressive agents, corticosteroids, anti-allergic, anti-coagulation or anti-platelet medication
18. History of drug addiction (positive drug screen) or excessive use of alcohol (weekly intake more than 28 units of alcohol), or psychological or other emotional problems that are likely to invalidate informed consent, or limit the ability of the subject to comply with the protocol requirements
19. Positive HIV1, or HIV2 serology
20. Positive results from the hepatitis serology which indicates acute or chronic hepatitis B or hepatitis C
21. Positive alcohol breath test
22. Vaccination with any vaccine within 4 weeks prior to dosing of the study medication
23. Any physical condition that would, in the opinion of the investigator, place the subject at an unacceptable health risk or risk of injury or render the subject unable to meet the requirements of the protocol
24. History of serious adverse reactions or hypersensitivity to any medicinal product
25. Smoking > 5 cigarettes/day or unable to refrain from smoking while confined to the CPU
26. Use of prescription, over-the-counter (OTC), herbal supplements (excluding hormonal contraceptives, one-a-day vitamins, acetaminophen) within 14 days prior to the first dose of study drug).

### Randomization criterion

Subjects should have a detectable antibody response to DC-TAB in their screening sample.

### Excluded prior and concomitant treatments

No medication other than the investigational product, contraceptives and vitamins taken once daily is allowed. However, should any treatment / medication, prescription and/or OTC, other than the investigational products be necessary to treat adverse events, the investigator must note the use of concomitant medication in the source documentation and the CRF. This record should include the drug name (trade name), the total daily dose, route of administration, the start and stop date of administration, and the indication for use (which should match the AE text for which the concomitant medication was administered).

### Withdrawal of subjects

A withdrawal is a subject who stops prematurely (for any reason).

If, during the course of the study, there is deterioration in the subject’s well being or the investigator feels that it is in the subject’s best interest to be withdrawn from the study, the subject can be withdrawn. In case a serious adverse event related to the study medication occurs, the subject must be withdrawn, unless doing so would harm the subject in the opinion of the investigator.

The subject has the right to withdraw from the study at any time and for any reason.

All subjects discontinuing should have a final visit including at least all safety assessments. Reasons for subject withdrawal will be documented in the CRF.

Subjects withdrawn after first administration of study medication will not be replaced. Subjects withdrawn prior to first administration of study medication will be replaced.

### Pausing and premature termination of the study

In case of safety or tolerability concerns, the sponsor may decide to temporarily pause the trial. When the trial is temporarily paused, the Competent Authorities (CA) and Ethics Committee will be notified immediately (but at least within 15 days from when the trial is temporarily paused). The notification will be made as a substantial amendment using the Notification of Amendment form and clearly explaining what has been halted (e.g. stopping recruitment and/or interrupting treatment of subjects already included) and the reasons for the temporary pause.

The study would resume only if review of the adverse events that caused the pause resulted in a recommendation from the review committee to permit continuation of the study. To restart a trial that has been temporarily halted, the sponsor will make the request as a substantial amendment using the Notification of Amendment form and by providing evidence that it is safe to restart the trial.

If the clinical observations in the study suggest that it may be unwise to continue, the study may be prematurely terminated by the sponsor. If the sponsor decides not to recommence a temporarily halted trial, the CA and Ethics Committees will be notified within 15 days of his decision, using the End of Trial Declaration form and including a brief explanation of the reasons for ending the trial. Additionally, the trial may be terminated by the sponsor at any time for administrative reasons.

Delta Crystallon BV. may stop this study for any of the following reasons:

- The centre cannot include an adequate number of subjects
- Serious and/or persistent non-compliance with the protocol
- Careless or premeditated false documentation in the CRF
- Inadequate co-operation with Delta Crystallon BV or its representatives
- Non-compliance with GCP and / or regulatory requirements
- The investigator requests discontinuation

Delta Crystallon BV has the right to close this study at any time, although this should occur only after mutual consultation. Should the study be closed prematurely, all study materials (for example completed, partially completed and blank CRFs and all unused investigational products) must be returned to Delta Crystallon BV.

Reasons that may require termination of the study include, but are not limited to:

- Enrolment of subjects is unsatisfactory
- Quality of data is unsatisfactory
- Data recording is inaccurate and / or incomplete on a chronic basis
- The incidence and / or severity of AEs indicate a potential health hazard caused by treatment with the investigational products
- Completed accrual and follow-up of subjects

# Treatments

## Treatments administered

The pharmacy will prepare all study medication for all subjects according to the treatment randomization list.

In Part 1, subjects will receive a single dose of study medication as intravenous bolus injection administered in the arm at day 1. The required amount of study medication will be administered to the subject via an intravenous catheter.

In Part 2, subjects will receive once daily study medication on 3 consecutive days administered as an intravenous bolus injection.

## Selection of doses in the study

Based on animal model data [Verbeek *et al.*, 2007], the effective dose in humans is expected to be in the range of approximately 0.2-0.4 mg/kg, or a fixed dose of approximately 12.5 - 25 mg per injection.

The NOAEL observed in rats following 28 days treatment was set at 20 mg/kg, which is equivalent to an HED of 3.2 mg/kg.

The NOAEL in female dogs could not be established following 15 days of treatment, whereas the NOAEL established for male dogs was 0.5 mg/kg following 15 consecutive dose administrations. The NOAEL for up to 5 days treatment was set at 12 mg/kg for both female and male dogs.

In order to limit the risk for the healthy subjects, a starting dose of 4 mg has been chosen for Part 1. The second group of subjects will receive 12.5 mg, the third group 25 mg and the fourth group 37.5 mg. If necessary, based on safety data obtained during the study, the doses of the second, third and fourth group may be adjusted downward.

## Methods of assigning subjects to treatment groups

Apart from his subject identification code, each subject whose eligibility has been confirmed will be allocated a sequential randomization number. Subjects will be assigned to a treatment code prepared by the CRO. Of each first group of subjects, one will be treated with placebo and the other with active medication.

## Blinding procedures

Sealed treatment randomization codes should be stored in the Investigator’s File or any other secure place at the investigator’s site.

Unblinding of the codes should only be done after closing the database or in case of emergency. Premature unblinding should always be documented and explained on the code envelope.

After completion of the study the code envelopes will be checked by the monitor.

The syringe will be prepared by the pharmacist who is not involved in the clinical conduct of the study. The labels in the syringes will contain the randomization number, subjects’ initials, and date and time of preparation of the syringe. In order to maintain blinding, syringes and infusion lines will be covered in non-transparant foil to avoid identification of the compound due to a slight difference in colour of the active IMP and placebo.

## Treatment compliance

Administration of study medication and any deviation hereof will be recorded in the CRF.

# Conduct of the study

## Screening and selection of subjects

The subject will receive verbal and written information concerning the study. The subject should be given adequate time to read the information leaflet and an opportunity to ask the investigator/delegate any questions.

Following the provision of subject information, written consent will be obtained from the subject prior to any study-specific procedure. The subject is not to enter the study if he/she has not understood the written and verbal information provided and/or has not personally signed and dated the consent form. A copy of the subject information leaflet and the signed informed consent will be provided to the subject and a copy will be retained by the investigator.

Subjects who fulfill the selection criteria will be enrolled into the study and enter a screening period to fully assess their suitability for treatment (randomization). The subjects will be allocated a subject number (subject identification code) at enrolment. During this period, subjects will be screened according to the inclusion and exclusion criteria and the schedule of assessments (see sections 4.4.1, 4.4.2 and 6.6).

If the assessments at screening do not reveal any condition inconsistent with the inclusion and exclusion criteria, the subject qualifies for treatment. Subsequently the subject will be assigned a medication code in addition to the subject number, which will ensure unambiguous identification throughout the study.

Subject identification codes, in addition to the identification numbers assigned by the centre, will be recorded on a subject identification code list, which will be kept on file at the study-site.

## Baseline periods

Subjects will be confined from day -1 until discharge from the unit on day 2 (Part 1) or day 4 (Part 2).

On the day prior to injection, subjects will be confined to the unit. On Day -1 and prior to study drug administration on day 1 subjects will undergo a series of assessments (see schedule of assessments, section 6.6). The values measured on day -1 and pre-dose on day 1 will be considered baseline measurements against which measurements during the treatment period will be compared.

Subjects are to refrain from exercise other than the normal daily activities until 24 hours after injection of study medication. In addition, subjects will refrain from smoking and alcohol consumption and limit intake of caffeinated drinks to a maximum of three units per day starting from the time of confinement on day ‑1 until discharge from the unit.

## Treatment & follow-up period

Subjects who have a pre-existing immunoresponse to DC-TAB as assessed by an ELISA may be randomized. See section 4.4.3

On day 1 (Part 1) and day 1, 2 and 3 (Part 2) study drug administration will take place in the morning. The following assessments will be performed: vitals signs (up to 4 hours after study drug administration), ECG, continuous cardiac monitoring (from -1 hours up to 4 hours after study drug administration), safety biochemistry and haematology, urinalysis and biomarkers. Twenty-four hours after last study drug administration, an ECG and physical examination will be performed, and AEs, vital signs and body temperature will be assessed. In addition, blood sampling for PK will be performed. Hereafter the subjects will leave the unit.

Subjects will return to the research unit for ambulatory visits on days 4 (Part 1 only), 8 and 15 for assessments according to the schedule of assessments, section 6.6.

## Final study visit

A final study visit will be performed on day 28. An ECG and physical examination will be performed, and AEs, vital signs and body temperature will be assessed. In addition, safety laboratory assessments and a pregnancy test, if applicable, will be performed.

## Early Termination Visit

In case of any early termination from the study, the subject will be asked to continue scheduled evaluations, complete an end-of-study evaluation, and be given appropriate care under medical supervision until the symptoms of any adverse event resolve or the subject’s condition becomes stable. All reasonable efforts should be undertaken to obtain the assessments as specified for the Follow-up visit in the schedule of assessments.

## Schedule of assessments

Table 1 Schedule of assessments – Part 1

|  | **Screening** |  |  |  |  |  | **Follow-up visit** |
| --- | --- | --- | --- | --- | --- | --- | --- |
|  | *Day*  *-28 to -1* | *Day -1* | *Day 1*  *(pre-dose)* | *Day 2* | *Day 4* | *Day 8* | *Day 28±2* |
| Confinement |  | X | X | X |  |  |  |
| Study drug administration |  |  | X |  |  |  |  |
| Written consent | X |  |  |  |  |  |  |
| Inclusion/Exclusion criteria | X |  | X |  |  |  |  |
| Demographic data | X |  |  |  |  |  |  |
| Medical History | X |  |  |  |  |  |  |
| Previous medication | X |  | X |  |  |  |  |
| Physical examination | X | X |  | X | B^2^ | B^2^ | X |
| Vital Signs | X |  | X | X | X | X | X |
| Body temperature | X |  | X | X | X | X | X |
| 12-Lead ECG | X |  | X | X | X |  | X |
| Cardiac monitoring (from -1 h to 4 h after dosing) |  |  | X |  |  |  |  |
| Urine Pregnancy Test | X | X |  |  |  |  | X |
| Safety Laboratory Tests (Haematology, Biochemistry, Urinalysis) | X | X |  | X | X |  | X |
| Serology, drug screen | X |  |  |  |  |  |  |
| Alcohol breath test | X | X |  |  |  |  |  |
| Adverse events |  | X | X | X | X | X | X |
| Concomitant medication |  | X | X | X | X | X | X |
| PK sampling^1^ |  |  | X |  |  |  |  |
| Blood samples for effect parameters | X |  | X |  |  | X | X |

^1^ Samples (relative to study drug administration) at pre-dose, t=10 minutes, t=20 minutes, t=30 minutes, t=1 hour, t=2 hours, t=4 hours, t=8 hours, t=16 hours, t=24 hours;

^2^ Brief physical examination (symptom guided)

Table 2. Schedule of assessments - Part 2

|  | **Screening** |  |  |  |  |  |  | **Follow-up visit** |
| --- | --- | --- | --- | --- | --- | --- | --- | --- |
|  | *Day*  *-28 to -1* | *Day -1* | *Day 1*  *(pre-dose)* | *Day 2* | *Day 3* | *Day 4* | *Day 8* | *Day 28 ± 2 days* |
| Confinement |  | X | X | X | X | X |  |  |
| Study drug administration |  |  | X | X | X |  |  |  |
| Written consent | X |  |  |  |  |  |  |  |
| Inclusion/Exclusion criteria | X |  | X |  |  |  |  |  |
| Demographic data | X |  |  |  |  |  |  |  |
| Medical History | X |  |  |  |  |  |  |  |
| Previous medication | X |  | X |  |  |  |  |  |
| Physical examination | X | X |  | B^2^ | B^2^ | X | B^2^ | X |
| Vital Signs | X |  | X | X | X | X | X | X |
| Body temperature | X |  | X | X | X | X | X | X |
| 12-Lead ECG | X |  | X |  |  | X | X |  |
| Cardiac monitoring (from -1 h to 4 h after dosing) |  |  | X | X | X |  |  |  |
| Urine Pregnancy Test | X | X |  |  |  |  |  | X |
| Safety Laboratory Tests (Haematology, Biochemistry, Urinalysis) | X | X |  |  |  | X | X | X |
| Serology, drug screen | X |  |  |  |  |  |  |  |
| Alcohol breath test | X | X |  |  |  |  |  |  |
| Adverse events |  | X | X | X | X | X | X | X |
| Concomitant medication |  | X | X | X | X | X | X | X |
| PK sampling |  |  | X^1^ |  | X^1^ |  |  |  |
| Blood sample for effect parameters | X |  | X |  |  | X | X | X |

^1^ Samples (relative to study drug administration) at pre-dose, t=10 minutes, t=20 minutes, t=30 minutes, t=1 hour, t=2 hours, t=4 hours, t=8 hours, t=16 hours, t=24 hours;

^2^ Brief physical examination (symptom guided)

## Compliance with the protocol

Any deviation from the protocol must be explained and the reason entered in the CRF. Any subject who is entered into the study but is found not to meet the protocol entry criteria will be considered for withdrawal as a protocol violator after consultation with the sponsor.

# Methodology of assessments

## Baseline variables

### Demographics

At screening, the following demographic data will be collected: gender, date of birth, race, body weight, height and smoking habit.

### Medical history

Complete medical history will be reviewed at screening. This will include evaluation for past and present cardiovascular, respiratory, gastrointestinal, renal, hepatic, neurological, endocrine, lymphatic, haematological, immunological, dermatological, psychiatric, genitourinary, and surgical history and rhinoconjunctivitis history and any other diseases or disorders.

### Previous Medication

The investigator should record the use of all medications used within 3 months prior to entry in the study, both prescribed and over-the-counter, on the CRF. This includes drugs used on a chronic and as-needed basis. The following will be recorded on the CRF: Drug name (trade name), total daily dose, route, start and end date and time, whether ongoing and indication.

All previous medication will be coded using the World Health Organization’s Drug Dictionary (WHO-DD).

## Safety variables

The safety and tolerability of the investigational products will be evaluated by monitoring the subject’s AE profile and local tolerability throughout the study, the assessment of routine safety tests, physical examinations (including vital signs), continuous cardiac monitoring and 12-lead ECG.

### Laboratory Parameters

Blood (approximately 8 ml of blood per visit) and urine specimens for safety laboratory testing will be taken according to the schedule of assessments (see section 6.6). The laboratory tests will be coordinated / performed by the local laboratory.

Haematology will include haemoglobin (Hb), haematocrit (Hct), red blood cell count (RBC), platelet count, white blood cell count (WBC) and absolute differential count (neutrophils, basophils, eosinophils, monocytes, and lymphocytes).

Biochemistry will include alanine aminotransferase (ALT), aspartate aminotransferase (AST), alkaline phosphatase (ALP), total bilirubin, gamma glutamyl transferase (GGT), lactate dehydrogenase (LDH), total protein, albumin, urea, creatinine, sodium, potassium and chloride.

Urinalysis (semi-quantitative analysis per dipstick) will include protein, glucose, nitrite, pH, ketones, and blood. If protein, nitrite or blood is abnormal in the urine sample, microscopy will be done. Urinalysis will be performed by the local laboratory.

Urine pregnancy tests will be performed on all females according to the schedule of assessments (see section 6.6). Pregnancy tests will be performed at the study centre. In the event that the subject is withdrawn, a urine pregnancy test will be performed at the last visit.

### Physical Examination

Physical examinations will be performed at all visits. The examinations will include the following: general appearance, HEENT (head, ears, eyes, nose and throat), skin, cardiovascular system, respiratory system, gastrointestinal system, nervous system and other. On days 4 and 8 of Part 1 and on days 2, 3 and 8 of Part 2, only a brief physical examination will be performed based on symptoms.

### Vital Signs

Blood pressure (systolic (SBP) and diastolic (DBP)), pulse rate and respiration rate after 10 minutes supine rest will be recorded at each visit. On dosing days, evaluations will be done pre-dose and, in addition, blood pressure and pulse rate will be assessed at 1 hour, 2 hours and 4 hours after dosing. Automatic or manual devices may be used. All devices must have been calibrated in the previous 12 months. Calibration certificates must be available on request.

### Body temperature

Oral body temperature will be measured at each visit. On dosing days, body temperature will be measured pre-dose and at 1 hour, 2 hours and 4 hours after dosing.

### 12-Lead Electrocardiogram

Computerised 12-lead ECG recordings will be obtained according to the schedule of assessments (see section 6.6). The following ECG parameters will be recorded: heart rate, RR interval, P wave duration, PR interval, QRS complex, QT and QTc according to Bazett and Fridericia (QTc will be calculated by the data management department). A clinical assessment of “Normal”, “Abnormal, not clinically significant” or “Abnormal, clinically significant” will be made.

### Continuous cardiac monitoring

Continuous cardiac monitoring will be performed from 1 hour pre-dose until 4 hours post-dose.

Any alarm of continuous cardiac monitoring will be visually inspected on the ward and the interpretation (normal/abnormal) will be recorded. Any clinically significant abnormality detected by the device and confirmed by the PI or his/her delegate will be printed out, retained as source data, and recorded as an adverse event as described in Section 11.2 of this protocol.

### Adverse Events including Local and Systemic Reactions

The investigator is responsible for recording all AEs, including any local and systemic reactions, observed during the study. Persisting reactions should be evaluated by the investigator and the investigational product could be discontinued if indicated. See section 11.2 for detailed information on AE collection.

### Concomitant medication/antidotes

The investigator should record the use of all concomitant medications, both prescribed and over-the-counter, on the CRF. This includes drugs used on a chronic and as-needed basis. The following will be recorded on the CRF: Drug name (trade name), total daily dose, route, start and end date and time, whether ongoing and indication.

All concomitant medication will be coded using the World Health Organization’s Drug Dictionary (WHO-DD) criteria.

## Pharmacokinetics

4 mL blood samples will be taken for assessment of serum concentrations levels of DC-TAB at pre-dose, t=10 minutes, t=20 minutes, t=30 minutes, t=1 hour, t=2 hours, t=4 hours, t=8 hours, t=16 hours and t=24 hours relative to dosing on day 1 and day 3 (Part 2 only). Within 2 hours after blood sampling, blood will be centrifuged. Immediately after centrifugation, serum will be stored in 2 labelled polypropylene tubes at ≤ -20°C for serum concentration analysis. All serum concentration analyses will be performed according to GLP after the study has been finalized.

DC-TAB in serum samples, stored frozen at -20°C, has been examined for structural stability by informal non-GLP studies, using detectability by a monoclonal antibody in a sandwich ELISA as parameters for integrity. DC-TAB was found to be fully stable under such conditions for a period of at least 6 months.

## Effect parameters

Four 5 mL blood samples will be taken for assessment of T-cell responses and antibody response. Peripheral blood samples are to be collected in heparin tubes to prevent blood clotting and kept at room temperature. Samples should be shipped to the sponsor by courier, ensuring delivery preferably within 4 h after sampling, but no later than 6 h after sampling.

T-cell responsiveness assays will be initiated on the day of sample collection. Peripheral blood mononuclear cells (PBMC) will be isolated from blood samples within 4-6 h after sampling, and immediately subjected to an assay for antigen-specific proliferation. In this test, PBMC will be labelled with the fluorescent dye carboxyfluorescein succinimidyl ester (CFSE) and placed in culture with varying doses of DC-TAB along with appropriate positive and negative controls. After 9 days, specific proliferation will be quantified by flowcytometric evaluation of the extent of CFSE dilution in the population of CD4+ T-cells in the culture, which is indicative of cell division. Proliferation will be quantified relative to that in control cultures without DC-TAB, and compared to reference antigens.

Antibody assays include a quantitation of total serum IgG titers against DC-TAB using a standard ELISA set up, and a bioactivity assay to evaluate neutralizing activity of serum antibodies. This activity will be evaluated by examining the inhibitory effects of serum dilutions on the ability of DC-TAB to induce TNF-α release by a macrophage cell line in culture.

All effect parameter assessments will be performed by Delta Crystallon, Leiden, the Netherlands.

# Study medication

## Identity of the investigational products

Investigational product

Product: DC-TAB

Formulation: solution for intravenous injection

Strength: 12.5 mg/ml

Vial volume: 1 ml

Placebo

Product: PBS

Formulation: solution for intravenous injection

## Drug manufacturing

The study medication is manufactured under supervision of the sponsor by Novozymes, Sweden. Manufacturing operations are in accordance with Good Manufacturing Practice.

All medication used in this study will be prepared and labeled according to the rules of Good Manufacturing Practice, ICH-GCP and local regulatory requirements.

Study medication labeling will contain the following items:

- Study number
- Product identification
- Concentration and volume
- Route of administration
- Expiry date
- Batch number
- Sponsor address and telephone number

## Drug storage and stability

The investigational product is to be stored frozen at -20ºC.

Stability studies are currently ongoing. Previous batches indicated that DC-TAB is stable at -20ºC for at least 36 months, and at +4ºC for at least 9 months. DC-TAB can be thawed and re-frozen for storage at -20ºC at least five times.

## Preparation, administration and dosage of treatment

DC-TAB is provided in ready-to-use 1 ml vials containing 12.5 mg/ml DC-TAB. The pharmacy of the unit will prepare the syringes according to the dosing schedule for the group under investigation (i.e. 4, 12.5, 25 and 37.5 mg). PBS will be added until a total volume of 3 ml is obtained (to limit the residual volume of investigational product in the syringe and needle).

Placebo (PBS) will be sourced from a commercial supplier. 3 ml PBS will be prepared in a syringe.

Syringes will be labeled with the subject’s randomization number, subject’s initials, and date and time of preparation of the syringe. After preparation syringes will be stored between +2 and +8ºC in a refrigerator. In order to maintain the blind, the syringes will be covered using non-transparant foil.

Subjects will receive the study medication within three hours after preparation as a bolus injection in the arm. Following injection, the intravenous catheter will be flushed with 2 ml PBS.

## Drug accountability

The sponsor or designate will request the investigator or responsible pharmacist to sign a receipt for the study drugs. All study medication supplied for the study should be kept in a locked secure place with appropriate pharmaceutical precautions.

A “Drug Accountability” record should be maintained by the person responsible for dispensing the trial medication to the subject. This should record which supplies are issued to which subjects and any drugs returned unused. Details of any supplies that are inadvertently damaged should be given on this record, which will be collected by the monitor at the end of the study.

All unused study medication should be kept and added to the drug accountability record. All study medication in these categories will be inventoried by the monitor during and at the conclusion of the study. The monitor will arrange for their secure disposal at the end of the study.

The drugs supplied for this study are only intended for use by subjects in this study. They must not be diverted for use by others.

# Data handling and record keeping

## Data collection

Data collection is the responsibility of the clinical trial staff at the site under the supervision of the Principal Investigator. During the study, the Investigator must maintain complete and accurate documentation for the study.

Conducting a trial and the related use of CRFs should not detract from the routine data recording in the source documents. It should be clearly marked in the medical records of the subject at the trial site that the subject is participating in a particular clinical trial. Source data must be available to document the existence of the subject and substantiate integrity of trial data collected. Source data must include the original documents related to the trial (e.g. ECG traces, laboratory prints), to the medical treatment, and history of subject.

All data obtained in the clinical trial described in this protocol will be recorded on worksheets (direct entry) or forms designed for recording AEs, comments, or medical history, etc. Data not recorded on direct entry worksheets will be transcribed to Case Report Forms (CRFs). All missing data will be explained. CRF items not done will be marked as “ND.” If an item is unknown or not applicable to the specified case, the space in the CRF will be marked “UNK” and “NA”, respectively. Data that has been collected but cannot be retrieved will also be marked “UNK.” All data entries will be made in permanent, black ink.

The CRFs or worksheets for the recording of trial data for each subject will be presented in a booklet or bundle. The actual time and date of assessments must always be entered on the CRFs or worksheets.

Each CRF or worksheet must be filled in neatly with a ballpoint pen only. The final authorization of the CRF or worksheet booklet or bundle is considered to be the "End Of Trial" (EOT) form. The PI signs the EOT form to signify review of the booklet and EOT form and to certify them to be complete and accurate.

At each visit of the monitor, the CRFs should be available for review.

The following information should be included in the source medical records:

- demographic data
- medical history and physical examination details
- adverse events and concurrent treatment(s)
- ECG data
- visit dates and dispensing of study medication
- information on the subject’s treatment
- hematology, biochemistry and urinalysis
- serology, drug screen

The following information may be recorded directly in the CRF and should be considered as source data:

- vital signs
- alcohol breath test

## Data management

Data management will be performed by Kendle.

The completed CRF and worksheets will be submitted to the clinical data management department. The data entry will be carried out using Oracle Clinical software, version 4.5. All data collected will be entered in the database. Two different operators will enter data in duplicate, and control checks between the two databases will be done until 100% concordance is reached.

Queries will be issued, e.g. on missing data, inconsistencies, illegible data, illegal values and unclearly corrected items. Resolution of queries will be implemented in the database.

The following type of errors may be resolved by the assigned data manager:

- Obvious errors in visit dates.
- Incorrect rounding of figures, unless rounding influences other data.
- Spelling errors, if obvious.

Any other errors will be queried by the data manager and resolved by the investigator.

Errors must be corrected by drawing a single thin line with ballpoint pen only, through the incorrect entry and by writing in the new value as close to the original one as possible. Corrections are to be initialled and dated by the person making the correction and, if deemed necessary, the reason for correction must be explained next to the correction made.

## Record keeping

The investigator will maintain a study file, which he/she should use to file the IB, protocol, drug accountability records, correspondence with the IEC/IRB, the sponsor and other study-related documents.

The investigator’s copy of the CRFs, study file, consent forms, drug accountability records and the subject identification list must be kept by the investigator for at least 15 years. These documents should be retained for a longer period, however, if required by the applicable regulatory requirements. It is the responsibility of the sponsor to inform the investigator/institution as to when the documents no longer need to be retained. In addition the investigator must make provision for the subjects’ records to be kept for the same period of time.

The sponsor will archive and retain all documents pertaining to the study for at least 15 years after the last approval.

# Statistical analysis and reporting

## Study parameters

### Safety parameters

- Adverse events
- Local tolerability
- Hematology and biochemistry laboratory parameters
- Urinalysis
- 12-Lead ECG
- Vital signs (blood pressure, pulse and respiration rate)
- Oral body temperature
- Physical examination

### Effect parameters

- Proliferative response by CRYAB-reactive peripheral blood CD4+ T-cells
- Level of CRYAB-reactive serum antibodies
- Neutralizing activity of CRYAB-reactive serum antibodies

### Pharmacokinetic parameters

- AUC_0-24h_
- AUC_0-∞_
- C_max_
- t _1/2_
- t _max_
- Kel

## Evaluability of data

All subjects who received at least one dose of study medication will be included in the safety analysis.

## Statistical analysis

Statistical analysis will be performed by Kendle. A statistical analysis plan will be prepared prior data base lock describing the data analysis in more detail. Deviations from the Statistical Analyses Plan will be justified in the study report. All statistical calculations will be performed using SAS version 9.

A statistical analysis plan will be prepared prior to database lock describing the data analysis in more detail.

### Handling of Missing and Incomplete Data

Handling of missing and incomplete data will be detailed in the statistical analysis plan.

### Safety data

Individual and summary blood pressures, heart rate, respiration rate, body temperature and ECG parameters will be presented using descriptive statistics which include tabular form with mean, median, standard deviation and range (min and max) as appropriate.

Adverse events will be coded by SOC and preferred term according to the current version of the Medical Dictionary for Regulatory Activities (MedDRA). Adverse events will be summarized by number of subjects and percentage of subjects by treatment group, MedDRA SOC and Preferred Term. Serious AEs, drug-related AEs and serious drug-related AEs will also be summarized.

### Pharmacokinetic data

Plasma concentrations will be listed and summarized by nominal time point, and treatment (number of subjects (N), mean, standard deviation, minimum, median maximum, coefficient of variation). Individual and mean plasma concentration vs. time profile plots will be produced for each dose level.

All pharmacokinetic calculations will be done using WinNonlin (version 5.2 or higher). Pharmacokinetic parameters will be listed and summarized by treatment.

Statistical analyses of pharmacokinetic data will include enrolled subjects who receive study treatment and complete the study. The AUC_last_, AUC_inf_ and C_max_ parameters will be analyzed both prior to and after natural log transformation, as applicable. The pharmacokinetic parameter estimates will be evaluated by Analysis of Variance.

Details will be described in the statistical analysis plan.

### Effect data

Continuous variables will be summarized descriptively providing, where applicable, the number of subjects (N), mean, standard deviation (SD), coefficient of variation, median, minimum (min) and maximum (max).

Changes from baseline will be summarized descriptively providing, where applicable, the number of subjects (N), mean, standard deviation (SD), median, minimum (min) and maximum (max).

### Demographic and background variables

Baseline demographic and background variables will be summarized for all subjects. For categorical variables, frequencies and percentages will be provided. For continuous variables, descriptive statistics including the sample size, mean, median, standard deviation and range, will be presented.

### Subject accountability

All subjects enrolled will be included in a summary of subject accountability. The frequency and percentage of subjects enrolled in the study, randomized, discontinued before completing the study, and completing the study will be summarized.

### Study medication compliance

A summary of study medication usage and compliance will be provided for all subjects by treatment group.

## Sample size justification

The number of subjects to be included is based on medical rather than statistical grounds. It is considered that the foreseen number of subjects per dose group will provide sufficient safety information to support the start of a subsequent multiple dose study in healthy volunteers.

## Study report

At the conclusion of the study, after the data are analyzed, the sponsor will prepare an integrated study report in accordance with ICH-E3. A draft copy of the report will be available for review by the PI. The report will be signed by the PI and appropriate persons from the sponsor.

# Adverse events

## Adverse event definition

An adverse event is any untoward medical occurrence in a patient or clinical investigation patient administered a pharmaceutical product and which does not necessarily have a causal relationship with this treatment. An adverse event (AE) can therefore be any unfavourable and unintended sign (including a laboratory finding), symptom or disease temporarily associated with the use of a medicinal product, whether or not related to the medicinal product.

Adverse events may include, but are not limited to:

- Subjective or objective symptoms spontaneously offered by the subject, parent/legal representative or observed by the investigator or medical staff.
- Laboratory abnormalities of clinical significance.

Disease signs, symptoms and/or laboratory abnormalities already existing prior to the use of the product are not considered adverse events after treatment unless they reoccur after the subject has recovered from the pre-existing condition or in the opinion of the investigator they represent a clinically significant exacerbation in intensity or frequency.

## Reporting adverse events

At each evaluation, the investigator will determine whether any adverse events have occurred. If any adverse events have occurred, they will be recorded on the adverse event report pages of the CRF. If known, the diagnosis should be recorded, in preference to the listing of individual signs and symptoms.

Any adverse event experienced by the subject from the time of signing the informed consent until the end of the study will be recorded in the CRF.

The investigator will be asked to assess the severity of the adverse drug/ biologic event using the following categories: mild, moderate and severe. This assessment is subjective and the investigator should use medical judgment to compare the reported adverse event to similar type events observed in clinical practice. Below are listed guidelines for severity assessment:

Mild: Symptom(s) barely noticeable to the subject or does not make the subject uncomfortable. The adverse event does not influence performance or functioning. Prescription drugs are not ordinarily needed for relief of symptom(s).

Moderate: Symptom(s) of a sufficient severity to make the subject uncomfortable. Performance of daily activities is influenced. Treatment of symptom(s) may be needed.

Severe: Symptom(s) of a sufficient severity to cause the subject severe discomfort. Severity may cause cessation of treatment with the study drug. Treatment for symptom(s) may be given.

The investigator will make a judgment regarding whether or not, in his/her opinion, the adverse event was related to study drug. The investigator will also evaluate any changes in laboratory values, make a determination as to whether the change is clinically important, and whether or not the changes were related to study drug. However, even if the investigator feels there is no relationship to the study drug, the adverse event or laboratory abnormality must be recorded in the CRF.

If the relationship between the study drug and the adverse event is defined as “not related”, then the adverse event must definitely be caused by the subject’s clinical state, or the study procedure/conditions.

A “remote/unlikely” relationship is defined as when the temporal association between the adverse event and the drug is such that the drug is not likely to have any reasonable association with the adverse event.

A “possible” relationship is defined as when an adverse event could have been produced by the subject’s clinical state or the therapy.

A “probable” relationship is defined as when the adverse event follows a reasonable temporal sequence from the time of drug administration, abates upon discontinuation of the drug and cannot be reasonably explained by the known characteristics of the subject’s clinical state.

A “definite” relationship is defined as when the adverse event follows a reasonable temporal sequence from the time of drug administration, abates upon discontinuation of the drug and reappears when the drug is introduced.

## Reporting of Pregnancy

A newly diagnosed pregnancy in itself will not be considered an AE unless it is suspected that the investigational product(s) interacted with a contraceptive method or had some association with the occurrence of pregnancy. A congenital anomaly as a result of this pregnancy is a serious adverse event (SAE).

Before study enrollment, women of childbearing potential must be advized of the importance of avoiding pregnancy during their study participation. All women of childbearing potential must be willing to undergo a pregnancy test before the first administration of the investigational product.

The investigator must immediately notify the sponsor of any pregnancy associated with the study exposure, including 30 days for females after the study who will forward Pregnancy Surveillance Form(s) for monitoring the outcome of the pregnancy.

In addition, the investigator must report to the sponsor follow-up information regarding the course of the pregnancy, including perinatal and neonatal outcome. Infants will be followed for a minimum of 6 months.

## Follow-up of adverse events

Adverse events will be monitored from the time the first study treatment is administered to the end of the study. Adverse events will be elicited by direct, non-leading questioning or by spontaneous reports. At each visit, the subject should be asked a non-leading question such as: “Do you feel different in any way since starting the last assessment?” Adverse events already recorded on a previous occasion, and designated as ‘continuing’, should be reviewed at each subsequent assessment. Any adverse event will be followed-up until the event or its sequelae resolve or stabilize at a level acceptable to the investigator and sponsor’s clinical monitor or his/her designated representative.

# Serious adverse events

## Serious adverse event definition

A Serious Adverse Event is any untoward medical occurrence that at any dose:

- Results in death
- Is life-threatening
- Requires subject hospitalization or prolongation of existing hospitalization
- Results in persistent or significant disability/incapacity
- Is a congenital anomaly/birth defect
- Is considered medically important; e.g. requires intervention to prevent permanent impairment or damage (for studies under IND)

Medical and scientific judgment should be exercised in deciding whether expedited reporting is appropriate in other situations, such as important medical events that may not be immediately life-threatening or result in death or hospitalization but may jeopardize the subject or may require intervention to prevent one of the other outcomes listed in the definition above. These should also be considered serious.

A death occurring during the study or which comes to the attention of the investigator within 30 days of study drug administration, whether considered treatment related or not, must be reported. In the event of an SAE the investigator may immediately stop treatment if it is considered in the best interest of the subject. In case a serious adverse event related to the study medication occurs, the subject must be withdrawn, unless doing so would harm the subject in the opinion of the investigator.

### Unexpected adverse drug reaction

“Unexpected adverse drug reaction” is defined as an adverse reaction, the nature or severity of which is not consistent with the Investigator’s Brochure.

### Life-threatening adverse event

Any adverse event that places the subject, in view of the reporter, at immediate risk of death from the reaction as it occurred, i.e. it does not include a reaction that, had it occurred in a more serious form, might have caused death.

### Hospitalization

This is defined as the subject being hospitalized overnight, or the subject’s hospital stay being prolonged for at least an additional night. Pre-planned hospital stays or stays for social reasons will not be considered hospitalization.

### Persistent or significant disability/incapacity

This is defined as any adverse event that resulted in a substantial disruption of a person’s ability to conduct normal life functions.

### Medical occurrence requiring intervention to prevent permanent impairment or damage

This is defined as any adverse event that may jeopardize the subject or subject and may require medical or surgical intervention to prevent death, life threatening, (prolonged) hospitalization, disability or congenital anomaly.

## Reporting serious adverse events and/or unexpected adverse drug reactions

Investigators are obliged to notify, by fax (or telephone), to the sponsor all serious adverse events and unexpected adverse drug reactions IMMEDIATELY (within 24 hours of the investigator becoming aware of the event). The investigator will be requested to supply as much detailed information regarding the event that is available at the time of the initial contact. The investigator is also required to submit follow-up reports to the monitor until the adverse event has resolved or in the case of permanent impairment, until the adverse event stabilizes.

**ANY SERIOUS ADVERSE EVENT WHETHER OR NOT RELATED TO THE STUDY DRUG MUST BE REPORTED IMMEDIATELY TO THE FOLLOWING DEPARTMENT:**

***Global Clinical Safety & Pharmacovigilance Kendle, Munich, Germany***

***Telephone: +49-89-993913-198***

***Fax: +49-89-993913-422***

For all SAEs a Serious Adverse Event Report Form that includes a detailed written description, anonymized copies of relevant subject records, autopsy reports, and other documents should be faxed to the above mentioned fax number, within 48 hours.

The responsible pharmacovigilance officer will immediately evaluate the SAEs for reporting to the appropriate regulatory agencies in consultation with the medical responsible person from the sponsor.

## Suspected Unexpected Serious Adverse Reaction (SUSAR) reporting

Kendle is responsible for the expedited reporting of SUSAR’s to the applicable regulatory authorities and Ethics Committee. Expedited reporting means not later than 15 days after the sponsor has first knowledge of the adverse reactions. For fatal or life threatening cases the term is maximal 7 days for a preliminary report with another 8 days for completion of the report.

# Ethics and protection of study participants

## Basic principles

This study will be conducted in compliance with the protocol, ICH-GCP, the Declaration of Helsinki and all applicable regulatory requirements.

## Independent Ethics Committee/Institutional Review Board approval

Written approval must be gained from the IEC/IRB prior to release of clinical supplies and commencement of the study.

The protocol, the IB, the IMPD, the subject information sheet, the consent form, the investigator’s curriculum vitae and the advertisements (if any) will be submitted by the principal investigator(s) to the Independent Ethics Committee (IEC) or Institutional Review Board (IRB). The IEC/IRB must conform to ICH-GCP.

A copy of the written approval must be provided to the sponsor.

Any amendments to the protocol or subject information and consent form will be submitted to the IEC/IRB of the investigational centre and written approval will be obtained for substantial amendments prior to implementation (see also section 14.1).

## Regulatory requirements

The study will only start in a study center after having received written approval from the relevant Regulatory Authorities.

## Insurance of the subject

The sponsor will cover this study by means of an adequate insurance of the subject which will be in place prior to the start of the study.

## Informed consent

It is the responsibility of the Investigator to obtain written Informed Consent from the subject. Information about the study will be given to the subject both verbally and in writing. The written subject information sheet will explain the objectives of the study, its potential risks and benefits. The investigator must be satisfied that the subject has understood the information provided before written consent is obtained.

It should be made clear that refusal to participate or withdrawal from the trial at any stage is without any prejudice to the subject’s subsequent care. No subject should be obliged to participate in the trial. The subject must be given ample opportunity to enquire about details of the trial. If there is any doubt as to whether the subject has understood the written and verbal information, the subject should not enter the study.

The subject must be made aware that the monitors, auditors, the IEC/IRB and regulatory authorities will be granted direct access to the study subjects source medical records without violating subject confidentiality, and to the extent permitted by applicable regulations.

If the subject agrees to participate in the study he will be asked to sign and date a consent form which will be kept by the Investigator. The subject information leaflet and a copy of the signed informed consent will be provided to the subject.

The signed consent forms will be retained by the investigator and made available (for review only) to the study monitor and auditor on request.

# Study administrative procedures

## Protocol amendments

Any changes to the study, which arise after approval of the protocol, must be documented as protocol amendments. Protocol amendments affecting the safety of the subject, the scope of the study and/or the scientific quality (i.e. significant amendments) should be submitted to regulatory authorities and to the IEC/IRB for approval. The changes will become effective only after approval by the sponsor, the responsible investigator, IEC/IRB and regulatory authorities. All other amendments (i.e. non-substantial amendments) will be submitted to the IEC/IRB and regulatory authorities for notification.

## Study monitoring

The sponsor of this study is responsible according to ICH-GCP guidelines for assuring proper study conduct with regard to protocol adherence and validity of the data recorded in the CRFs.

The sponsor has therefore assigned a study monitor to this study. His/her duties are to assist the investigator in the maintenance of complete, legible, well organized, and easily retrievable data. In addition, the monitor will ensure that the investigator understands all applicable regulations concerning the clinical evaluation of an investigational drug, as laid down in ICH GCP guidelines.

The investigator agrees to allow the monitor direct access to the study drug dispensing and storage area and to all clinical data of the study subjects for the above purposes and agrees to assist the monitor in these activities. The investigator accepts that the monitor will visit the center at regular intervals to review and verify the data collected. The monitor will regard all information that is supplied to him or her as strictly confidential.

## Subject confidentiality

Each participating site will maintain appropriate medical and research records for this trial, in compliance with ICH E6 GCP and local law requirements for the protection of confidentiality of subjects.

Clinical research personnel assigned by the sponsor will require direct access to the subject’s source data for data verification. The confidentiality of all the subjects’ identities will be maintained. Source data are all information, original records of clinical findings, observations, or other activities in a clinical trial necessary for the reconstruction and evaluation of the trial. Examples of these original documents and data records include, but are not limited to, hospital records, clinical and office charts, laboratory notes, memoranda, subjects’ diaries or evaluation checklists, pharmacy dispensing records, recorded data from automated instruments, copies or transcriptions certified after verification as being accurate and complete, microfiches, photographic negatives, microfilm or magnetic media, x-rays, and subject files and records kept at the pharmacy, at the laboratories, and medico-technical departments involved in the clinical trial.

Only subjects’ date of birth and study number will be used on CRFs and in all study correspondence. No material bearing a subject’s name will be kept on file by the sponsor or clinical research personnel assigned by the sponsor.

All information disclosed to the investigator by the sponsor or persons assigned by the sponsor shall be treated by the investigator as strictly confidential. The Investigator shall only use such information for the purpose of conducting the clinical trial described in this protocol, and the investigator agrees not to disclose such information to any third party except those of his colleagues and employees who are assisting in the conduct of the study and who are bound by the obligations of confidentiality.

## Use of information and publications

Information concerning the study drug, patent applications, processes, unpublished scientific data, the Investigator's Brochure and other pertinent information is confidential and remains the property of the sponsor. Details should be disclosed only to the persons involved in the approval or conduct of the study. The investigator may use this information for the purpose of the study only. It is understood by the investigator that the sponsor will use the information obtained during the clinical study in connection with the development of the drug and therefore may disclose it as required to other clinical investigators or to regulatory agencies. In order to allow for the use of the information derived from this clinical study, the investigator understands that he has an obligation to provide the sponsor with all data obtained during the study.

The study may be considered for publication or presentation at (scientific) symposia and congresses.

The investigator will be entitled to publish or disclose the results only after written approval of the manuscript by the sponsor. The sponsor will be allowed to review all transcripts, texts of presentations and abstracts related to the study at least three months prior to the intended submission for publication or any other disclosure. This is necessary to prevent premature disclosure of trade secrets or patent-protected information and is in no way intended to restrict publication of facts or opinions formulated by the investigator.

The sponsor will inform the investigator of any objection or question arising within 30 days of receipt of the proposed publication. After written approval is obtained, the manuscript is free for publication.

## Quality assurance

The sponsor may conduct (or have conducted) periodical inspections of the study by reviewing the data obtained as well as the procedural aspects. This may include on-site inspections and source data checks. Direct access to source documents is required for the purpose of these periodical inspections. Any such access will be confidential and the identity of the subject will not appear on any copies made of the records.

## Regulatory inspections

Regulatory authorities may also wish to have direct access to the medical records of subjects participating in the study for the purpose of audit or inspection. Any such access will be confidential and the identity of the subject will not appear on any copies made of the records. The inspection may occur during the study, or at any time following the study.

# Study documentation and supplies

The sponsor will provide the investigator with the following documents:

- Study protocol
- Investigator's Brochure
- IMPD
- Study medication with all necessary documentation
- Study contract

In order to begin the study, the responsible investigator is required to provide the following documentation to the sponsor:

- Signed confidentiality agreement
- Signed investigator's agreement
- Signed contract
- Subject information leaflets and informed consent forms
- CRFs
- Adverse event report forms
- Investigator’s study file
- Independent Ethics Committee approval, stating clearly the sponsor's name, study number and investigational drug, including a list with names and qualifications of all members of the Independent Ethics Committee and a list of documents reviewed.
- Recent versions of signed and dated curricula vitae of all investigators and sub investigators
- Signature sheet, documenting signatures, initials and trial related duties of all study site personnel
- Laboratory normal ranges, signed and dated by the responsible laboratory employee
- Medical/laboratory/technical procedures/tests methods of assay, certifications or accreditation’s or established quality control or other validations, where applicable.

At the end of the study, the sponsor is responsible for the collection of:

- All sponsor’s left-over study documentation
- Left-over study medication

# Reference list

Bajramovic JJ, Lassmann H, and van Noort JM (1997) Expression of alpha B-crystallin during lesional development in multiple sclerosis. *J. Neuroimmunol*. **78,** 143-151

Bajramovic JJ, Van der Goes A, Koevoets C, Van Sechel AC, Newcombe J, Cuzner ML, and van Noort JM (2000) Presentation to T-cells of alpha B-crystallin in MS lesions, an early event following inflammatory demyelination. *J. Immunol.* **164,** 4359-4366.

Bhat NR, and Sharma KK (1999) Microglial activation by the small heat shock protein, alpha-crystallin. *NeuroReport* **10**, 2869-2873.

Chabas D, Baranzini SE, Mitchell D, Bernard CC, Ritling SR, Denhardt DT, Sobel RA, Lock C, Karpuj M, Pedotti R, Heller R, Oksenberg JR and Steinman L (2001) The influence of the proinflammatory cytokine, osteopontin, on autoimmune demyelinating disease. *Science* **294**, 1731-1735

Frohman EM, Racke MK, and Raine CS (2006) Multiple sclerosis – the plaque and its pathogenesis. *New Engl J Med* **354**, 942-955.

Masilamoni JG, Vignesh S, Kirubagaran R, Jesudason EP, and Jayakumar R (2005a) The neuroprotective efficacy of alpha-crystallin againsty acute inflammation in mice. *Brain Res Bull* **67**, 235-241.

Masilamoni JG, Jesudason EP, Barathi SN, and Jayakumar R (2005b) The protective effect of alpha-crystallin against acute inflammation in mice. *Biochim Biophys Acta* **1740**, 411-420.

Masilamoni JG, Jesudason EP, Baben B, Jabaraj CE, Dhandayuthapani S, and Jayakumar R. (2006) Molecular chaperone alpha-crystallin prevents detrimental effects of neuroinflammation. *Biochim. Biophys. Acta* **1762**, 284-293.

McFarland HF, and Martin R (2007) Multiple sclerosis: a complicated picture of autoimmunity. *Nat Immunol* **8,** 913-919.

Ousman SS, Tomooka BH, van Noort JM, Wawrousek EF, O’Conner K, Hafler DA, Sobel RA, Robinson WH and Steinman L (2007) Protective and therapeutic role for αB-crystallin in autoimmune demyelination. *Nature*, **448:** 474-479

Sinclair C, Mirakhur M, Kirk J, Farrell M, and McQuaid S (2005) Upregulation of osteopontin and alpha B-crystallin in the normal-appearing white matter of multiple sclerosis: an immunohistochemical study utilizing tissue microarrays. *Neuropathol Appl Neurobiol* **31**, 292-303.

Tajouri L, Mellick AS, Ashton KJ, Tannenberg AEG, Nagra RM, Tourtelotte WW, and Griffiths LR (2003) Quantitative and qualitative changes in gene expression patterns characterize the activity of plaques in multiple sclerosis. *Mol Brain Res* **119**, 170-183.

Van Noort JM, Van Sechel AC, Bajramovic JJ, El Ouagmiri M, Polman CH, Lassmann H and Ravid R (1995) The small heat-shock protein alpha B-crystallin as candidate autoantigen in multiple sclerosis. *Nature* **375**, 798-801.

Van Noort JM, Verbeek R, Polman CH, Meilof J and Amor S (2006) Autoantibodies against alpha B-crystallin, a candidate autoantigen in multiple sclerosis, are part of a normal human immune repertoire. *Multiple sclerosis* **12,** 287-293.

Verbeek R, Van der Mark K, Wawrousek EF, Plomp AC and van Noort JM (2007) Tolerization of an established alpha B-crystallin-reactive T-cell response by intravenous antigen*. Immunology* 121: 416-426.
