# Supplementary material for: Therapeutic Intervention in Multiple Sclerosis with Alpha B-Crystallin: A Randomized Controlled Phase IIa Trial
Source: PLoS One. 2015 Nov 23;10(11):e0143366. doi: 10.1371/journal.pone.0143366 (PMC4657879; doi:10.1371/journal.pone.0143366)
Supplement: S2 Protocol — A Phase IIa, randomized, double-blind, placebo-controlled, exploratory, dose-ranging study to evaluate the safety, effectiveness and pharmacokinetics of three courses of DC-TAB treatment in patients with multiple sclerosis. (DOCX) [file pone.0143366.s003.docx]

A Phase IIa, **randomized, double-blind, placebo-controlled, exploratory, dose-ranging** study to evaluate the safety, effectiveness and pharmacokinetics of three courses of DC-TAB treatment in patients with multiple sclerosis

| **Study no.** | DC-002 |
| --- | --- |
| **EudraCT no.** | 2011-004475-36 |
| **Investigational product** | DC-TAB |
| **Indication** | Multiple sclerosis |
| **Development phase** | IIa |
| **Date of protocol** | 20 April 2012 |
| **Version of protocol**  **Precedes version** | Version 1.1 (including amendment 1)  Version 1/14 December 2011 |
| **Sponsor** | Delta Crystallon BV  p/a Zernikedreef 9  2333 CK Leiden  The Netherlands  Tel +31 88 866 9696  Fax +31 71 518 1927 |
| This study will be performed in compliance with Good Clinical Practices (ICH-GCP), including the archiving of essential documents. | |
| **This document is property of Delta Crystallon BV and contains confidential information. It may not be forwarded to third parties without explicit written prior consent from Delta Crystallon BV. Reproductions, either in part or in whole, may not be published or copied in any manner, without the explicit written consent of Delta Crystallon BV.** | |

# Study synopsis

| **Title of Study**  A Phase IIa, randomized, double-blind, placebo-controlled, exploratory, dose-ranging study to evaluate the safety, effectiveness and pharmacokinetics of three courses of DC-TAB treatment in patients with multiple sclerosis | |
| --- | --- |
| **Number of sites and countries**  5 sites/Bulgaria | |
| **Study period**  Start in Q2 2012 – Q4 2013 | **Phase of development**  IIa |
| **Objectives**  *Primary Objective*  To evaluate the safety and tolerability of three single doses of DC-TAB administered intravenously with 2-month intervals in patients with multiple sclerosis.  *Secondary objectives*   1. To evaluate the antigen-specific T-cell tolerance inducting effect of three single doses of DC-TAB administered intravenously with 2-month intervals in patients with multiple sclerosis. 2. To evaluate the clinical effects of three single doses of DC-TAB with 2-month intervals administered intravenously in patients with multiple sclerosis. 3. To evaluate the pharmacokinetics of DC-TAB in patients with multiple sclerosis. 4. To evaluate the effect of DC-TAB on levels of serum antibodies against CRYAB/DC-TAB | |
| **Methodology**  This study is a randomized, double-blind, placebo-controlled, exploratory, dose-ranging Phase IIa study to evaluate the safety, tolerability, T-cell tolerance inducing effect, clinical effects and pharmacokinetics of human alpha B-crystallin (CRYAB), the active ingredient of DC-TAB.  At entry patients will be randomized to one of the treatments, placebo, 7.5 mg DC-TAB, 12.5 mg DC-TAB or 17.5 mg DC-TAB in a 1:1:1:1 fashion. Patients will receive a single intravenous bolus injection which will be repeated twice with two month intervals during the 6-month monitoring period. Following a single intravenous bolus administration of DC-TAB, a state of T-cell tolerance is expected to be established within days.  The study consists of two parts, a treatment period of 24 weeks and a follow up period of an additional 24 weeks. Patients will return to the hospital weekly during the first month, and monthly thereafter.  The primary analysis will be performed on the treatment period, i.e. after all patients have completed 24 weeks; an additional analysis will be performed once all patients have completed the study. Patients and site study personnel will remain blinded throughout the study.  After 12 and 24 patients have completed 4 and after 24 patients have completed 12 weeks of follow-up a partially blinded safety review will be conducted by an independent DSMB to verify safety of the intervention in MS patients. | |
| **Number of patients planned**  32 patients are planned to be randomized (8 per treatment arm) | |
| **Diagnosis and main criteria for inclusion or exclusion**  Main inclusion criteria:   - Clinically definite, relapsing multiple sclerosis, according to the McDonald criteria, and abnormal MRI scan consistent with MS - Neurologically stable - At least one clinical relapse over the previous year, or two relapses over the past two years, or one or more gadolinium-enhancing MRI lesion(s) at the time of screening. - An EDSS score ≤ 5.5 - Age 18-55 years   Main exclusion criteria:   - Primary progressive multiple sclerosis - Systemic corticosteroid treatment for at least 3 consecutive days less than 30 days before screening - Plasmapheresis, or intravenous gammaglobulins less than 2 months before screening - Treatment with natalizumab less than one year before screening - Previous immunosuppressive treatment (e.g. cyclophosphamide or mitoxantrone) - Previous treatment with any leukocyte-targeting monoclonal antibody (e.g. rituximab, alemtuzumab, daclizumab) - Previous treatment with oral immune-modulatory agents (cladribine, fingolimod, laquinimod, fumarate)   Note: other current disease-modifying agents such as interferon-beta or glatiramer acetate are expected to not interfere with the intervention and are therefore allowed. | |
| **Test product, dose and mode of administration**  The investigational product, DC-TAB, is a sterile solution of highly purified recombinant human alpha B crystallin (CRYAB) at 12.5 mg/mL in phosphate-buffered saline, pH 7.2.  The intervention includes a single intravenous bolus injection repeated twice at 2-month intervals. Three different dose levels of DC-TAB will be evaluated:  Low dose: single dose of 7.5 mg  Intermediate dose: single dose of 12.5 mg  High dose: single dose of 17.5 mg  Patients will receive three courses of a single bolus injection of DC-TAB in this study. | |
| **Duration of treatment**  Single dose, repeated every 2 months. | |
| **Reference therapy, dose and mode of administration, batch number**  Phosphate-buffered saline (PBS) will be used as a placebo/reference.  It will be administered intravenously in the same volume and in the same manner as DC-TAB. | |
| **Criteria for evaluation** | |
| **Effect parameters**   - - Proliferative CD45RO+ (memory) and CD4+ (helper) T-cell response to DC-TAB   - New or enlarging gadolinium-enhancing lesions on T1-weighted MRI   - Clinical relapse   - EDSS   - MSIS-29   **Effect endpoints**   - T-cell responses (CD4+ and CD45RO+) to DC-TAB throughout the 48-week study - Cumulative number of new or enlarging gadolinium-enhancing MRI lesions between weeks 4 and 24 and between weeks 4 and 48 after treatment, relative to baseline - The number of clinical relapses between week 0 and week 24, and week 0 and week 48 - Change in EDSS score between week 0 and week 24, and week 0 and week 48 - Change in MSIS-29 score between week 0 and week 24, and week 0 and week 48 - Level of CRYAB/DC-TAB-reactive serum antibodies | |
| **Safety parameters**   - Number and frequency of adverse events (including local tolerability) - ECG parameters - Clinical chemistry and hematology parameters - Urinalysis - Vital signs and body temperature - MRI signs and symptoms | |
| **Pharmacokinetic parameters**   - AUC_0-24h_ - AUC_0-∞_ - C_max_ - t _1/2_ - t _max_ - Kel | |
| **Statistical methods**  **Statistical analysis methods**  The primary analysis will be done on the treatment period, i.e. once all patients completed the 24 week timepoint. An additional analysis will be performed at week 48.  Safety population  The safety population will comprise of all patients who received at least one dose of treatment.  Full analysis population (FAS)  The full analysis set (FAS) consists of all patients who received at least one dose of treatment and in whom at least one effect assessment is available beyond baseline.  Sample size  Since this is the first study in patients with MS the sample size is based on medical and practical grounds rather than statistical grounds.  Safety parameters  No formal statistics will be employed   - Adverse events (AEs) will be coded and presented using MedDRA. The overall incidence, the incidence of related as well as the incidence of serious adverse events (SAEs) will be summarized per dose and by body system in tables. - Local tolerability will be presented by overall incidence by treatment group per symptom. - Vital signs (supine blood pressure and heart rate) and body temperature: Descriptive statistics (n, mean, standard deviation, median, minimum, and maximum) will be provided for blood pressure, heart rate and body temperature and for changes from baseline. - Hematology, biochemistry and urinalysis parameters: Descriptive statistics (n, mean, standard deviation, median, minimum, and maximum) will be provided on values at baseline and all post-dose assessments. Descriptive statistics will be calculated on the change from baseline values. Out-of-normal-range values will be indicated for each parameter. The change in out-of-normal-range values after administration and at follow up versus baseline will be summarized per group by means of shift-tables. - ECG: Descriptive statistics (frequency, mean, standard deviation, median, minimum, and maximum) on ECG parameters will be provided at baseline and at all post-baseline assessments. Changes from baseline values will be calculated and descriptive statistics will be computed. Shift tables will also be provided to indicate changes in ECG parameters over time.   PK parameters  Descriptive statistics (n, mean, standard deviation, median, minimum, and maximum, plus Geometric mean and CV for C_max_ and AUC and the derived parameters) will be provided for the PK parameters.  Evaluations of dose-proportionality and effects of multiple dosing will be performed.  Effect parameters  Descriptive statistics (n, mean, standard deviation, median, minimum, and maximum) at each visit will be provided for all effect parameters and as well as change from baseline (n, mean, standard deviation, median, minimum, and maximum).  A Student’s T-test will be performed to analyze the changes in the absolute percentage of proliferated T-cells and of the cell division index over time.  Details on the analysis methods to be used for other effect parameters will be specified in the statistical analysis plan. | |

# Sponsor’s signatures

**A Phase IIa, randomized, double-blind, placebo-controlled, exploratory, dose ranging study to evaluate the safety, effectiveness and pharmacokinetics of three courses DC-TAB treatment in patients with multiple sclerosis**

On behalf of the sponsor Delta Crystallon BV.

p/a Zernikedreef 9

2333 CK Leiden

The Netherlands

By my signature below I agree to the terms of this study protocol

_____________________________ ____________________

E.H.G. Venneker, M.D. Date

Afforce Healthcare BV.

Author & Sponsor Medical Responsible Person

_____________________________ ____________________

J.M. van Noort, Ph.D. Date

Delta Crystallon BV

Chief Scientific Officer

# Principal Investigator signature

**A Phase IIa, randomized, double-blind, placebo-controlled, exploratory, dose-ranging study to evaluate the safety, effectiveness and pharmacokinetics of three courses of DC-TAB treatment in patients with multiple sclerosis**

By my signature below I agree to conduct this clinical trial in accordance with the protocol, ICH-GCP, the Declaration of Helsinki, government regulations and national laws, including those applying to institutional/ethics review and informed consent.

______________________________ ____________________

Name investigator: Date

This document contains confidential information, which should not be copied, referred to, released or published without prior written approval from Delta Crystallon BV. Investigators are cautioned that the information in this protocol may be subject to change and revision.

# Table of Contents

[Study synopsis 2](#_Toc310967605)

[Sponsor’s signatures 5](#_Toc310967606)

[Principal Investigator signature 6](#_Toc310967607)

[Table of Contents 7](#_Toc310967608)

[List of abbreviations and definition of terms 11](#_Toc310967609)

[1 Introduction 13](#_Toc310967610)

[1.1 Background 13](#_Toc310967611)

[1.2 The role of alpha B-crystallin (CRYAB) in MS 14](#_Toc310967612)

[1.3 Investigational product 15](#_Toc310967613)

[1.4 Non-clinical data 16](#_Toc310967614)

[1.5 Clinical data 16](#_Toc310967615)

[1.6 Study rationale and risk/benefits 17](#_Toc310967616)

[1.6.1 Study rationale 17](#_Toc310967617)

[1.6.2 Risks 18](#_Toc310967618)

[1.6.3 Benefits 19](#_Toc310967619)

[2 Investigators and study administrative structure 20](#_Toc310967620)

[3 Study objectives 21](#_Toc310967621)

[3.1 Primary Objective 21](#_Toc310967622)

[3.2 Secondary objectives 21](#_Toc310967623)

[4 Investigational plan 22](#_Toc310967624)

[4.1 Overall study design and plan – description 22](#_Toc310967625)

[4.2 Study flow chart 22](#_Toc310967626)

[4.3 Discussion of study design, including choice of control groups 22](#_Toc310967627)

[4.4 Study population 23](#_Toc310967628)

[4.4.1 Inclusion criteria 23](#_Toc310967629)

[4.4.2 Exclusion criteria 23](#_Toc310967630)

[4.4.3 Excluded prior and concomitant treatments 24](#_Toc310967631)

[4.4.4 Withdrawal of patients 25](#_Toc310967632)

[4.4.5 Pausing and premature termination of the study 25](#_Toc310967633)

[5 Treatments 27](#_Toc310967634)

[5.1 Treatments administered 27](#_Toc310967635)

[5.2 Selection of doses in the study 27](#_Toc310967636)

[5.3 Methods of assigning patients to treatment groups 27](#_Toc310967637)

[5.4 Blinding procedures 27](#_Toc310967638)

[5.5 Treatment compliance 28](#_Toc310967639)

[6 Conduct of the study 29](#_Toc310967640)

[6.1 Screening and selection of patients 29](#_Toc310967641)

[6.2 Treatment period (week 0-24) 29](#_Toc310967642)

[6.3 Follow-up period (week 24-week 48) 30](#_Toc310967643)

[6.4 Early Termination 30](#_Toc310967644)

[6.5 Schedule of assessments 31](#_Toc310967645)

[6.6 Compliance with the protocol 33](#_Toc310967646)

[7 Methodology of assessments 34](#_Toc310967647)

[7.1 Baseline variables 34](#_Toc310967648)

[7.1.1 Demographics 34](#_Toc310967649)

[7.1.2 Medical history 34](#_Toc310967650)

[7.1.3 Previous Medication 34](#_Toc310967651)

[7.1.4 McDonald criteria 34](#_Toc310967652)

[7.2 Safety variables 35](#_Toc310967653)

[7.2.1 Safety laboratory Parameters 35](#_Toc310967654)

[*7.2.2* Urine pregnancy tests 35](#_Toc310967655)

[7.2.3 Physical Examination 35](#_Toc310967656)

[7.2.4 Vital Signs 36](#_Toc310967657)

[7.2.5 Body temperature 36](#_Toc310967658)

[7.2.6 12-Lead Electrocardiogram 36](#_Toc310967659)

[7.2.7 Adverse Events including local and systemic reactions 36](#_Toc310967660)

[7.2.8 Concomitant medication/antidotes 36](#_Toc310967661)

[7.3 Pharmacokinetics 36](#_Toc310967662)

[7.4 Effect variables 37](#_Toc310967663)

[7.4.1 T-cell response 37](#_Toc310967664)

[7.4.2 Antibody assessment 37](#_Toc310967665)

[7.4.3 MRI 38](#_Toc310967666)

[7.4.4 Clinical relapse 38](#_Toc310967667)

[7.4.5 EDSS 38](#_Toc310967668)

[7.4.6 MSIS-29 38](#_Toc310967669)

[8 Study medication 39](#_Toc310967670)

[8.1 Identity of the investigational products 39](#_Toc310967671)

[8.2 Drug manufacturing 39](#_Toc310967672)

[8.3 Drug storage and stability 39](#_Toc310967673)

[8.4 Preparation, administration and dosage of treatment 39](#_Toc310967674)

[8.5 Drug accountability 40](#_Toc310967675)

[9 Data handling and record keeping 41](#_Toc310967676)

[9.1 Data collection 41](#_Toc310967677)

[9.2 Data management 42](#_Toc310967678)

[9.3 Record keeping 42](#_Toc310967679)

[10 Statistical analysis and reporting 43](#_Toc310967680)

[10.1 Study parameters 43](#_Toc310967681)

[10.1.1 Safety parameters 43](#_Toc310967682)

[10.1.2 Effect parameters and endpoints 43](#_Toc310967683)

[10.1.3 Pharmacokinetic parameters 43](#_Toc310967684)

[10.2 Evaluability of data 43](#_Toc310967685)

[10.3 Statistical analysis 44](#_Toc310967686)

[10.3.1 Handling of Missing and Incomplete Data 44](#_Toc310967687)

[10.3.2 Safety data 44](#_Toc310967688)

[10.3.3 Pharmacokinetic data 44](#_Toc310967689)

[10.3.4 Effect data 44](#_Toc310967690)

[10.3.5 Demographic and background variables 45](#_Toc310967691)

[10.3.6 Patient accountability 45](#_Toc310967692)

[10.3.7 Study medication compliance 45](#_Toc310967693)

[10.4 Blinded Safety Review Analysis 45](#_Toc310967694)

[10.5 Sample size justification 45](#_Toc310967695)

[10.6 Study report 45](#_Toc310967696)

[11 Adverse events 46](#_Toc310967697)

[11.1 Adverse event definition 46](#_Toc310967698)

[11.2 Reporting adverse events 46](#_Toc310967699)

[11.3 Reporting of Pregnancy 47](#_Toc310967700)

[11.4 Follow-up of adverse events 47](#_Toc310967701)

[12 Serious adverse events 48](#_Toc310967702)

[12.1 Serious adverse event definition 48](#_Toc310967703)

[12.1.1 Unexpected adverse drug reaction 48](#_Toc310967704)

[12.1.2 Life-threatening adverse event 48](#_Toc310967705)

[12.1.3 Hospitalization 48](#_Toc310967706)

[12.1.4 Persistent or significant disability/incapacity 49](#_Toc310967707)

[12.1.5 Medical occurrence requiring intervention to prevent permanent impairment or damage 49](#_Toc310967708)

[12.2 Reporting serious adverse events and/or unexpected adverse drug reactions 49](#_Toc310967709)

[12.3 Suspected Unexpected Serious Adverse Reaction (SUSAR) reporting 49](#_Toc310967710)

[13 Ethics and protection of study participants 50](#_Toc310967711)

[13.1 Basic principles 50](#_Toc310967712)

[13.2 Independent Ethics Committee/Institutional Review Board approval 50](#_Toc310967713)

[13.3 Regulatory requirements 50](#_Toc310967714)

[13.4 Insurance of the patient 50](#_Toc310967715)

[13.5 Informed consent 50](#_Toc310967716)

[14 Study administrative procedures 52](#_Toc310967717)

[14.1 Protocol amendments 52](#_Toc310967718)

[14.2 Study monitoring 52](#_Toc310967719)

[14.3 Patient confidentiality 52](#_Toc310967720)

[14.4 Use of information and publications 53](#_Toc310967721)

[14.5 Quality assurance 53](#_Toc310967722)

[14.6 Regulatory inspections 53](#_Toc310967723)

[15 Study documentation and supplies 54](#_Toc310967724)

[16 Reference list 55](#_Toc310967725)

# List of abbreviations and definition of terms

Abbreviations

| AE | Adverse Event/Adverse Experience |
| --- | --- |
| AUC | Area Under the Curve |
| BLLQ | Below the Lower Limit of Quantification |
| BMI | Body Mass Index |
| BUN | Blood Urea Nitrogen |
| CA | Competent Authorities |
| CFSE | Carboxyfluorescein Succinimidyl Ester |
| CI | Confidence Interval |
| CIOMS | Council for International Organizations of Medical Sciences |
| C_max_ | Maximum Concentration |
| CRF | Case Report Form |
| CRO | Contract Research Organization |
| CRYAB | Alpha B-crystallin (used to refer to endogenous occurring alpha B-crystallin) |
| CSF | Cerebrospinal Fluid |
| CV | Coefficient of Variation |
| DBP | Diastolic Blood Pressure |
| DC-TAB | Delta Crystallon Therapeutic Alpha B-crystalline (used to refer to the IMP) |
| EDSS | Expanded Disability Status Scale |
| eGFR | Estimated Glomerular Filtration Rate |
| EOT | End Of Trial |
| FAS | Full Analysis Set |
| FDA | Food and Drug Administration |
| GCP | Good Clinical Practice |
| GMP | Good Manufacturing Practice |
| GLP | Good Laboratory Practice |
| Hb | Haemoglobin |
| HCT | Haematocrit |
| HED | Human Equivalent Dose |
| HIV | Human Immunodeficiency Virus |
| IB | Investigator’s Brochure |
| ICF | Informed Consent Form |
| ICH | International Conference on Harmonization |
| IEC | Independent or Institutional Ethics Committee |
| Kel | Elimination Rate Constant |
| LLQ | Lower Limit of Quantification |
| MDRD | Modification of Diet in Renal Disease |
| MedDRA © | Medical Dictionary for Regulatory Activities |
| MS | Multiple Sclerosis |
| MSIS-29 | Multiple Sclerosis Impact Scale |
| MTD | Maximum Tolerated Dose |
| n | Number (typically refers to patients) |
| NOAEL | No Observed Adverse Effect Level |
| OTC | Over-the-Counter |
| PI | Principal Investigator |
| PK | Pharmacokinetics |
| QA | Quality Assurance |
| QC | Quality Control |
| SAE | Serious Adverse Event/Serious Adverse Experience |
| SBP | Systolic Blood Pressure |
| SOP | Standard Operating Procedure |
| t_1/2_ | Elimination half-life |
| t_max_ | Time to Reach Maximum Plasma Concentration |
| VEP | Visually Evoked Response |
| WHO | World Health Organization |
|  |  |

# Introduction

## Background

Multiple Sclerosis (MS) is a chronic inflammatory disease of the human central nervous system (CNS) [1]. MS affects around 2 million people worldwide, predominantly in Europe and North-America where prevalence rates are around 1 in 1,000. Women are affected 2-4 times more often than men. Clinical manifestations of MS usually appear for the first time around the age of 30, and they develop in largely unpredictable and variable patterns of accumulating clinical deficits. Especially at the early stages, MS often progresses in episodes or relative worsening followed by episodes of spontaneous partial recovery. This clinical manifestation of MS is called ‘relapsing MS’. The cause of MS is recurrent focal inflammatory demyelination at varying sites throughout the CNS, most often in white matter regions. These foci of inflammation, referred to as MS lesions, lead to destruction of myelin and axons. This causes impaired nerve signal conduction, and subsequent neurological symptoms including paralysis, disturbed vision, fatigue, cognitive defects and pain.

The genetic factor in the development of MS largely hinges on the major histocompatibility complex which encodes so-called MHC proteins, pivotal immune-regulatory proteins, underscoring the notion that the immune system plays a crucial role in disease [2]. As with most chronic disorders, environmental factors are also relevant. These include exposure to sunlight, intake of vitamin D, smoking, and prior infection with Epstein-Barr virus (EBV) [3, 4]. In the absence of any direct causative link with a bacterial or viral infection, however, MS is generally regarded as autoimmune, driven by an adaptive T-cell reaction against an endogenous myelin-associated antigen in the CNS. This notion is supported by the effects of disease-modifying agents.

**Current therapeutic approaches in MS**

Currently approved treatment options in MS include two versions of interferon-beta 1a (Avonex and Rebif), two versions of interferon-beta 1b (Betaseron and Extavia), the immune-modulatory substance glatiramer acetate (Copaxone), the cytostatic drug mitoxantrone (Novantrone), the anti-adhesion molecule monoclonal antibody Natalizumab (Tysabri), and oral fingolimod (Gilenya) as registered drugs to modify the course of relapsing MS. For most of these drugs, their exact mode of action, and the biological range of their action, remains to be fully established. Without exception, however, current treatment options aim at interference with general immune functions (and sometimes CNS functions). While these functions may well be involved in the development of MS lesions, they are relevant to many other processes in the human body as well. This particularly applies to functions affected by cytostatic drugs (such as mitoxantrone), enzyme or receptor inhibitors (such as fingolimod), as well as by monoclonal antibodies (Natalizumab; and others under investigation such as Rituximab, Alemtuzumab and Campath). These drugs target key populations of immune cells, or a fundamental property of these cells, such as their ability to migrate. As has already been underscored by the potentially lethal side effects of Natalizumab, and the recent withdrawal of cladribine because of safety concerns, it may be expected that the therapeutic potential of non-selective immune-modulatory drugs will be offset to at least some extent by side effects, short term or long term. Especially the long-term effects of recently approved treatment options remain to be established.

This consideration highlights the importance of exploring more sophisticated strategies for modifying the course of MS, such as targeting only immune responses that are relevant to the disease process. In this context, antigen-specific tolerization represents an attractive alternative that may not only be more effective, but also present a more favourable side effect profile and less burden to patients.

## The role of alpha B-crystallin (CRYAB) in MS

A first crucial feature of CRYAB is its repeated accumulation in the CNS of MS patients. From time to time, CRYAB accumulates at 10- to 20-fold higher levels than normal in small groups of clustered oligodendrocytes, and in the myelin sheaths that are produced by these cells [5-9]. The trigger for this focal accumulation is currently unclear, although it is almost certainly endogenous in nature, and recurrent. CRYAB is a member of the family of stress-inducible proteins, also known as heat shock proteins. Its accumulation in oligodendrocytes must therefore reflect the recurrent emergence of some form of local stress, such as neuronal/axonal stress or degeneration, hypoxia, or accumulation of stress-inducing metabolites. CRYAB is an endogenous neuroprotectant and anti-inflammatory protein, acting both inside and outside cells in the CNS [9, 10]. Inside cells, it counteracts induction of apoptosis (programmed cell death), and it protects intracellular proteins from the potentially detrimental effects of oxidative stress or other insults. Outside cells, it induces immune-suppressive activities of macrophages including microglia, the brain’s macrophages. Also in other tissues, soluble extracellular CRYAB exerts anti-inflammatory therapeutic effects as evidenced in a variety of animal models [10-15]. The most likely mode of action in these cases is CRYAB-mediated alternative activation of local macrophages [9]. In itself, therefore, the accumulation of CRYAB is likely meant to protect the CNS from a variety of different types of stress or insults, and to mitigate inflammation. Focal CRYAB accumulation also occurs at so-called pre-active MS lesions, which are considered to reflect a first reversible stage of an MS lesion. At the site of a pre-active MS lesion, a full-blown inflammatory demyelinating MS lesion may well develop later on, but the pre-active lesion may also resolve [9]. Accumulation of CRYAB in pre-active MS lesions occurs without any involvement of the peripheral immune system, since peripheral immune cells are recruited into the site only at a later stage. This clarifies that CRYAB accumulation in the CNS of MS patients is not simply the consequence of an active MS lesion, but actually precedes the development of such lesions, and is triggered by an endogenous signal.

The second crucial feature of CRYAB is the fact that it is a major target antigen for circulating autoreactive memory T cells and serum antibodies, which are found in all adult humans [5, 9, 16]. This feature is unique to humans, since other mammals including rodents and dogs do not possess a natural state of immune reactivity to CRYAB. The mechanism driving the establishment of the CRYAB-reactive repertoire in humans is not yet fully clarified, but it likely involves infection with rather common viruses such as Epstein-Barr virus (EBV) earlier in life. At least in cell culture, EBV induces CRYAB production in infected B cells, and its presentation via MHC class II molecules to helper T cells [17]. CRYAB-directed autoimmune reactivity could thus be established at the time of the first EBV infection in humans. It is apparently maintained for life, consistent with the life-long persistent also of EBV. In support of this notion, epidemiological studies have shown EBV infection to essentially be a prerequisite for the development of MS. In itself, the permanent state of peripheral autoreactivity to CRYAB -found in both MS patients and healthy subjects alike- does not cause any disease. The protein is not routinely available for immune recognition, since it is expressed at meaningful levels only inside cells in normal healthy tissues and organs, shielded from immune recognition. This is supported by animal model studies which have shown that the experimental introduction of immune reactivity to CRYAB does not induce any disease in normal mice [18]. The only meaningful extracellular deposit of CRYAB currently known is that in CNS myelin. Since helper T cells are generally reactive only to extracellular antigens, this explains how accumulated myelin-associated CRYAB poses a risk in the CNS, while the same protein is expressed safely inside a variety of other cell types at the same time.

Key to the treatment rationale proposed here is the notion that MS lesions are caused by the response of CRYAB-reactive T cells to the very high local levels of accumulated CRYAB in oligodendrocytes and myelin. Such T cells are actively recruited into the CNS along with other leukocytes, at least in part due to activation of microglia by CRYAB itself [9]. Upon such activation, microglia secrete cytokines that activate the blood-brain barrier, and chemokines that promote migration of leukocytes. Thus, CRYAB accumulation itself appears to contribute to non-specific recruitment of T cells into the CNS, including CRYAB-reactive T cells. In response to an encounter with their target antigen, these CRYAB human T cells secrete significant amounts of interferon-γ, which turns local microglia and macrophages into destructive inflammatory cells. Somewhat paradoxically therefore, the intended neuroprotectant CRYAB in this way may become the inadvertent trigger for a destructive inflammatory process. This notion predicts that elimination of CRYAB-reactive T cells from the circulation should eliminate the fuel for this destructive process, while not interfering with the natural protective role of CRYAB.

## Investigational product

Based on the above considerations, Delta Crystallon aims to selectively suppress the activity of T cells in the circulation in MS patients that are reactive to CRYAB. While this does not change CRYAB accumulation inside the CNS (which is considered beneficial in its own right), it does eliminate the other crucial factor which is believed to contribute to the development of MS lesions.

Following up on animal model data on intravenous tolerance induction with CRYAB in mice [19], Phase I data on intravenous tolerance induction with DC-TAB (containing CRYAB as the active ingredient) in healthy volunteers indicate that in healthy humans, the reactivity of CRYAB-reactive T cells can be rapidly and effectively suppressed by a single intravenous administration of soluble CRYAB. Upon monitoring antigen-specific proliferative responses in peripheral blood T cells, it was found that CRYAB-induced T-cell suppression became apparent within days, and lasted for at least 28 days. After 6-8 months, T-cell reactivity was found to be partially restored in some subjects. The observed effects are consistent with CRYAB-induced activation of antigen-specific, inducible regulatory T cells (iTreg) in the periphery, which suppress the existing memory T-cell response to CRYAB itself, but much less so, if at all, to other antigens. While the dynamics of iTreg induction and longevity in humans are still incompletely known, the currently available data suggest that they have a limited lifespan *in vivo* [20]. These data, consistent with Phase I data on the reversibility of the tolerizing effect on T cells, suggest that maintenance of a long-term state of specific T-cell suppression requires repeated administration, for example with 2-month intervals. When applied to MS patients, Delta Crystallon expects the continued suppression of CRYAB-directed T-cell responses to suppress the development of inflammatory MS lesions and, thus, expects it to halt progression of MS. The intervention is not expected to lead to any meaningful functional repair of previously suffered damage. While CRYAB as a soluble protein has been shown to exert anti-inflammatory effects as a chaperokine both in cell culture and in different animal models [9-15], these effects are considered likely irrelevant to the present intervention, or at best only supportive. As has become apparent in cell culture models, natural anti-CRYAB antibodies that are present in the serum of adult humans will likely neutralize the anti-inflammatory chaperokine activity of CRYAB when it is administered intravenously.

## Non-clinical data

The NOAEL observed in rats following 28 days dosing was set at 17.5 mg/kg, the highest dose tested in toxicology studies. In all animals, a significant increase was observed in serum protein, serum globulin, and in spleen size and weight, consistent with the development of an antibody response against DC-TAB.

A study was conducted in beagle dogs who received DC-TAB in doses up to 5 mg/kg. After approximately 8-10 consecutive days of intravenous DC-TAB administration, treatment-related clinical symptoms started to develop immediately after administration in all treatment groups except for the male dogs receiving 0.5 mg/kg which all remained symptom-free. Clinical symptoms included pallor of otherwise well-perfused tissues such as the tongue and membranes in eyes and mouth, often preceded by brief reddening. More severe clinical signs included lethargy, spasms, abnormal posture and gait, and abnormal breathing. In a few cases, mild secretory symptoms or pallor already developed after 5-6 days. All the above clinical symptoms reversed within minutes after DC-TAB administration. Only a weak correlation was observed between the severity of clinical signs and the DC-TAB dose administered. In line with the findings in the above range-finding and maximum tolerated dose (MTD) studies, females tended to develop slightly more severe signs than males. In a rather abrupt reversal, all of these clinical signs stopped appearing after 12-14 days of treatment in almost all cases.

All pre-clinical data in dogs, the more sensitive species, indicate that there is a crucial difference between DC-TAB administration in naïve animals as compared to animals that had received DC-TAB before. Upon repeated administration, and with a minimum time interval of five days between a first and consecutive dose(s), significant clinical symptoms emerge immediately upon administration of such a consecutive dose, even a low dose. In naïve animals on the other hand, up to five consecutive doses of 12 mg/kg DC-TAB did not trigger any adverse effects.

The data do not allow formal definition of a NOAEL in female dogs following 15 days administration. In male dogs, the NOAEL is 0.5 mg/kg following 15 days administration. In naïve female and male dogs, the NOAEL up to five consecutive doses is 12 mg/kg.

Cardiovascular, respiratory and neurobehavioral safety pharmacology studies did not reveal any toxicologically significant effect of DC-TAB administered up to 17.5 mg/kg on any of these body systems.

For more details on the pre-clinical data please refer to the IB.

## Clinical data

A phase I, placebo-controlled, double-blind clinical study on the safety, tolerability, pharmacokinetics and T-cell tolerizing effect of DC-TAB in healthy volunteers has been finalized. In this study, the effects have been examined of single i.v. dosing (using 4, 12.5, 25 and 37.5 mg doses) in a first part of the study, and three consecutive daily i.v. doses of DC-TAB (using 10, 25 and 37.5 mg doses) in a second.

Single dose, bolus administration of DC-TAB up to 37.5 mg was well tolerated and safe. In addition, daily dosing of up to 37.5 mg for three consecutive days was well tolerated and safe in healthy volunteers. Following multiple dosing of high concentrations DC-TAB, mild to moderate injection site reactions were observed. The incidence of these reactions was lower when the concentration of DC-TAB in the bolus injections was lowered.

No changes were detected in serum antibody levels against CRYAB over a 28-day period following any of the treatment regimens.

Pharmacokinetic parameters indicate that serum levels of DC-TAB decrease with a half-life of around 60-75 min at all doses tested.

Immunological data indicate a statistically significant reduction in both CD4+ and CD8+ memory (CD45RO^+^) T-cell responses against CRYAB following a single dose level of 12.5 mg, becoming apparent already within 8 days after i.v. administration of DC-TAB. This effect was maintained for at least 28 days. In half of the subjects, the T-cell responses to CRYAB were suppressed to levels considered to reflect non-responsiveness. Antigen-specific T-cell responsiveness (partially) recovers 6-8 months after treatment. These findings are consistent with the notion that i.v. administration of DC-TAB induces antigen-specific inducible regulatory FoxP3+ T cells (iTregs), which functionally suppress the response by other T cells. Consistent with what is known on the dynamics of such a response, the suppressive effect was found to be partially lost after 6-8 months.

## Study rationale and risk/benefits

### Study rationale

This study is designed to evaluate the safety and pharmacokinetics of three different dose levels of intravenous DC-TAB. DC-TAB is given as a single dose and repeated twice with a 2-month interval. In addition, the effects of the treatment on the development of MRI lesions, on T-cell tolerance, and on levels of CRYAB/DC-TAB-reactive serum antibodies will be explored.

DC-TAB aims to selectively suppress the activity of potentially pathogenic T cells reactive to CRYAB in the circulation of MS patients. Based on Phase I data in healthy subjects, as discussed above, it is expected that suppression of peripheral T-cell reactivity to CRYAB can be achieved by a single intravenous administration of DC-TAB, repeated with 2 month intervals. Induction of inducible, antigen-specific regulatory FoxP3^+^ T cells is the most likely mechanism involved. Once CRYAB-reactive T-cell responses are suppressed, it is expected that new inflammatory demyelinating MS lesions will no longer develop for a certain period. Due to the limited telomer length of inducible regulatory T cells in humans, however, their life span is likely limited to a few months only. For this reason, re-emergence of a CRYAB-specific T-cell response after treatment is likely to graduallybecome manifest again after 2-3 months. It is an important goal of the study to clarify, the dynamics of such a recovery of the T-cell response in MS patients.

The current protocol for tolerance induction in a Phase II study is based on the Phase I data. Although there are no *a priori* reasons, nor any experimental data to suggest that tolerance induction in MS patients is regulated any different from that in healthy subjects, the dose level required for optimal tolerance induction, and the persistence of the tolerant state, does require verification in MS patients prior to embarking on a larger study in MS patients. Also the safety of the intervention, notably including repeated administration with 2-month intervals, needs to be established in MS patients.

### Risks

Below, some points of consideration relevant to the risk profile of DC-TAB are briefly discussed. Current Phase I data indicate that single as well as multiple i.v. dosing for three consecutive days of DC-TAB up to 37.5 mg are safe and well tolerated in healthy volunteers. Data obtained so far indicate that levels of peripheral T-cell reactivity and serum antibodies against CRYAB/DC-TAB are similar in healthy volunteers and MS patients.

**Temporary boost of T-cell responses by intravenous DC-TAB**

Temporarily boosting pro-inflammatory (not regulatory) T-cell responses to DC-TAB rather than suppressing them would represent a safety risk, since it would perhaps allow pathogenic mechanisms to develop, similar to those mediating experimental autoimmune encephalomyelitis in laboratory animals. While it is conceivable that intravenous DC-TAB could induce a temporary increase in T-cell responses to CRYAB before inducing a final state of tolerance, there are no indications that this happens in humans upon a single i.v. administration of DC-TAB at the intended doses. At the functional dose of DC-TAB, the T-cell response to CRYAB as monitored after 8 as well as 28 days in healthy human subjects was suppressed rather than increased.

**Antibody formation as the result of intravenous DC-TAB**

The role of antibodies in MS, if any, remains to be clarified. While it is conceivable that intravenous DC-TAB could lead to an increase in anti-CRYAB/DC-TAB antibodies, Phase I data have revealed that this does not happen in healthy subjects at the intended dose. It is considered unlikely that this would be any different in MS patients.

**Chaperokine activity of intravenous DC-TAB**

CRYAB exerts anti-inflammatory and neuroprotective effects as a chaperokine in different animal models of (neuro)inflammation [10-15]. The mechanism behind this effect likely involves alternative activation of macrophages and macrophage-like cells by DC-TAB, via engagement of a combination of scavenger receptors and Toll-like receptors. *In vitro* studies have demonstrated that natural human serum antibodies to DC-TAB inhibit this activity by blocking CRYAB-receptor interactions, even at high serum dilution. When given to humans, therefore, intravenous DC-TAB will become largely neutralized in its chaperokine activity. If neutralization would be incomplete, the chaperokine activity of DC-TAB would be expected to result in an anti-inflammatory innate immune response [9], and to temporarily inhibit development of inflammatory damage, thus assisting in the goal of suppressing disease.

**Reversibility of the tolerized state**

The intervention with DC-TAB is intended to induce tolerance but such a state will most likely not last indefinitely. As the result of natural regeneration of T-cell repertoires, and the likely decline in the numbers and/or activity of antigen-specific regulatory FoxP3+ T cells following their induction, normal pro-inflammatory T-cell reactivity likely returns over a period of several months. Phase I data indicate that partial reversal of the tolerizing effect is apparent after 6-8 months. No information is available on development of the tolerant state between 28 days and 6-8 months. Upon reversal of tolerance, patients can be treated again to maintain a sufficiently persistent state of tolerance. Phase I data clarifying the safety and tolerability of consecutive daily dosing over a 3-day period do not raise *a priori* concerns over the safety of repeated administration with 2-month intervals, instead of daily intervals. The effects, however, of these extended time intervals between different doses remains to be established.

### Benefits

The intervention is expected to suppress T-cell reactivity to CRYAB, and as a consequence, halt the formation of new inflammatory demyelinating MS lesions. As evidenced by animal model data, as well as by preliminary Phase I data on human tetanus toxoid responses, the intervention is antigen-specific, or at least largely antigen specific. For this reason, DC-TAB is not expected to impact in any way on peripheral immune reactivity against any other antigen. Relative to current disease-modifying drugs in MS, which all involve general immune modulation, treatment with DC-TAB, when successful, will thus offer an improved level of selectivity, and is not expected to suffer from side effects due to any general immune-modulatory activity.

# Investigators and study administrative structure

Sponsor’s contact Dr J.M. van Noort, Ph.D.

Chief Scientific Officer

Delta Crystallon BV

Zernikedreef 9

2333 CK Leiden

The Netherlands

Tel: +31 (0)88 866 9696

Fax: + 31 (0)71 518 1901

Mobile: +31 (0)6 5394 6231

Medical responsible person/ E.H.G. Venneker, M.D.

project leader Senior Partner

Afforce Healthcare BV

Veraartlaan 8

2288 GM Rijswijk

The Netherlands

Tel: +31 (0)70 710 1431

Fax: +31 (0)70 710 1437

Mobile: +31 (0)6 5155 9672

CRO for monitoring, PSI CRO AG

datamanagement/biostatistics Baarerstrasse 113a

and pharmacovigilance 6300 Zug

Switzerland

Laboratory for pharmacokinetics ALPHALYSE A/S

Unsbjergvej 4

DK-5220 Odense SØ

Denmark

Tel: +45 6310 6500

Laboratory for effect markers Central Reference Laboratory for Immunology

National Center for Infectious and Parasitic Diseases

26 Yanko Sakazov Blvd.

Sofia 1504

Bulgaria

Laboratory for antibody assays Delta Crystallon BV

Zernikedreef 9

2333 CK Leiden

The Netherlands

Tel: +31 (0)71 518 1541

Fax: + 31 (0)71 518 1901

Laboratory for MRI analysis VU Medical Centre

F. Barkhof, C. Polman

Dept of radiology and dept of neurology

De Boelelaan 1118

1081 HZ Amsterdam

The Netherlands

# Study objectives

## Primary Objective

To evaluate the safety and tolerability of three single doses of DC-TAB administered intravenously with 2-months intervals in patients with multiple sclerosis.

## Secondary objectives

1. To evaluate the antigen-specific T-cell tolerance inducting effect of three single doses of DC-TAB administered intravenously with 2-months intervals in patients with multiple sclerosis.
2. To evaluate the clinical effects of three single doses of DC-TAB with 2-months intervals administered intravenously in patients with multiple sclerosis.
3. To evaluate the pharmacokinetics of DC-TAB in patients with multiple sclerosis.
4. To evaluate the effects of three single doses of DC-TAB with 2-month intervals administered intravenously to patients with multiple sclerosis on serum levels of CRYAB/DC-TAB-reactive antibodies.

# Investigational plan

## Overall study design and plan – description

This study is a randomized, double-blind, placebo-controlled, exploratory, dose-ranging Phase IIa study to evaluate the safety, tolerability, T-cell tolerance inducing effect, clinical effects, pharmacokinetics, and effects on serum antibodies of human alpha B-crystallin (CRYAB), the active ingredient of DC-TAB.

Patients who fulfill all eligibility criteria will be randomized to one of the treatments, placebo, 7.5 mg DC-TAB, 12.5 mg DC-TAB or 17.5 mg DC-TAB in a 1:1:1:1 fashion. Patients will receive a single i.v. bolus injection treatment which will be repeated twice with 2-month intervals during the 6-month monitoring period. By a single intravenous bolus administration of DC-TAB, a state of T-cell tolerance is expected to be established within days. Each month, T-cell responses to DC-TAB will be assessed (the results of which will be known two weeks after blood sampling). The interval period between treatments may be adjusted in case the duration of the tolerizing effect in patients is different from what is currently expected.

The study consists of two parts, a treatment period of 24 weeks and a follow up period of an additional 24 weeks. Patients will return to the hospital weekly during the first month, and monthly thereafter.

The primary analysis will be performed on the treatment period, i.e. after all patients have completed 24 weeks; an additional analysis will be performed once all patients have completed the study. Patients and site study personnel will remain blinded throughout the study.

After 12 and 24 patients have completed 4 and 12 weeks of follow-up, a partially blinded safety review will be conducted by the DMC to verify safety and adequate tolerization by the intervention in MS patients.

## Study flow chart

Figure 4‑1 Study flow chart

|  |  |  | | | |  |  |
| --- | --- | --- | --- | --- | --- | --- | --- |
|  | | DC-TAB 7.5 mg | | | |  | |
| Screening | | DC-TAB 12.5 mg | | | | Follow-up | |
|  |  | DC-TAB 17.5 mg | | | |  |  |
|  |  | Placebo | | | |  |  |
|  |  |  |  |  |  |  |  |
|  | Rx | Rx | Rx |  |  |  |  |

## Discussion of study design, including choice of control groups

A double-blind, parallel design has been chosen to minimize the chance of bias.

Inclusion of a placebo control group will allow for a minimum of bias, and a correction for possible placebo effects. As patient’s clinical status is frequently checked, and since the use of certain other frequently used disease-modifying MS treatments like interferon-beta and glatiramer acetate is allowed during the course of the study, the use of a placebo control group is considered ethical.

As this study is the first study in patients, the number of patients per treatment group for this study is not based on statistical considerations, but based on medical/scientific and practical grounds.

A DSMB will be established to further secure patient safety as this is the first-in-patient study conducted with DC-TAB.

## Study population

Thirty-two (32) male and female patients with relapsing multiple sclerosis between 18 and 55 years of age.

### Inclusion criteria

1. Clinically definite relapsing multiple sclerosis, according to the McDonald criteria
2. Abnormal MRI consistent with MS
3. Neurologically stable for at least one month
4. At least one clinical relapse over the previous year, or two relapses over the past two years, or one or more gadolinium-enhancing MRI lesion(s) at the time of screening.
5. An EDSS score ≤ 5.5
6. Age 18-55 years
7. Body weight less than 130 kg
8. Use of adequate and stable contraception for 3 months prior to study initiation, during the course of the study and 30 days thereafter. Sexually active males must use a condom. Sexually active females must use double-barrier contraception or hormonal contraceptive (oral, transdermal, vaginal ring, implants), or must have undergone clinically documented total hysterectomy and/or oophorectomy, surgical sterilization, or be postmenopausal defined by amenorrhea for at least 12 months and confirmed with a FSH ≥ 40 mIU/ml.
9. If patients claim abstinence as their method of contraception, they must be willing to agree to use condoms if they become sexually active from 14 days prior to the first dose of the study drug through 90 days beyond the conclusion of the study.
10. Being informed of the nature and aims of the study, and having given written consent to participate in this study in accordance with local laws and requirements
11. Being willing to comply with the protocol, and understand the information given, and the text of the consent form

### Exclusion criteria

1. Primary progressive multiple sclerosis
2. Use of systemic corticosteroid treatment for more than 3 days within 30 days prior to screening
3. Plasmapheresis, or intravenous gammaglobulins less than 2 months before screening
4. Treatment with natalizumab less than one year before screening
5. Previous immunosuppressive treatment (e.g. cyclophosphamide or mitoxantrone)
6. Previous treatment with any leukocyte-targeting monoclonal antibody (e.g. rituximab, alemtuzumab, daclizumab)
7. Previous treatment with oral immune-modulatory agents (cladribine, fingolimod, laquinimod, fumarate)
8. Pregnant women, women planning to become pregnant and breastfeeding women
9. A history of or currently active clinically significant cardiac (including clinically significant ECG abnormalities in the opinion of the PI), pulmonary, gastrointestinal, hepatic, renal, pancreatic, or neurological disease
10. ALT, AST and/or gamma-GT above 3 times the upper limit of normal
11. Serum creatinine above 1.5 times the upper limit of normal or an eGFR < 60 ml/min/1.73 m^2^
12. Hemoglobin < 7.0 mmol/l for females and < 8 mmol/l for males; leucocytes > 20*10^9^/l or < 3.5*10^9^/l; platelets < 125*10^9^/l
13. SBP > 160 mmHg and/or DBP > 100 mmHg
14. Acute respiratory or other active infections
15. Fever (body temperature > 38.0 °C on day 1
16. Blood donation or significant blood loss within 90 days of first study medication dosing
17. Plasma donation within 7 days of first study medication dosing
18. Recipients of blood or blood products in the last 6 months
19. Participation in another clinical study within 90 days of the start of this trial or planning participation in another clinical trial during this study or in the 4 weeks after last visit
20. Taking anti-coagulation or anti-platelet medication with the exception of NSAID’s.
21. History of drug addiction (positive drug screen) or excessive use of alcohol (weekly intake more than 28 units of alcohol), or psychological or other emotional problems that are likely to invalidate informed consent, or limit the ability of the patient to comply with the protocol requirements
22. Vaccination with any vaccine within 4 weeks prior to dosing of the study medication
23. History of serious adverse reactions or hypersensitivity to any medicinal product
24. History of a malignancy other than skin cell basalioma 5 years prior to screening
25. Any physical condition that would, in the opinion of the investigator, place the patient at an unacceptable health risk or risk of injury or render the patient unable to meet the requirements of the protocol

### Excluded prior and concomitant treatments

The following medication is prohibited throughout the study:

1. High-dose systemic corticosteroid treatment unless given for treatment of relapses
2. Plasmapheresis, or intravenous gammaglobulins
3. Treatment with natalizumab
4. Immunosuppressive treatment (e.g. cyclophosphamide or mitoxantrone)
5. Treatment with any leukocyte-targeting monoclonal antibody (e.g. rituximab, alemtuzumab, daclizumab)
6. Treatment with oral immune-modulatory agents (cladribine, fingolimod, laquinimod, fumarate)

Treatment with other disease-modifying agents such as interferon-beta and glatiramer acetate should be avoided as much as possible, but are allowed if the investigator considers it necessary for these agents to be given.

Should any treatment / medication, prescription and/or OTC, other than the investigational products be necessary for treatment of adverse events, the disease or as prophylaxis, the investigator must note the use of concomitant medication in the source documentation and the CRF. This record should include the drug name (trade name), the total daily dose, route of administration, the start and stop date of administration, and the indication for use (which should match the AE text for which the concomitant medication was administered).

### Withdrawal of patients

A treatment withdrawal is a patient who stops treatment prematurely (for any reason).

If, during the course of the study, there is deterioration in the patient’s well being or the investigator feels that it is in the patient’s best interest to be stop study treatment, the patient treatment can be discontinued. In case a serious adverse event related to the study medication occurs, the study treatment must be stopped permanently for this patient, unless doing so would harm the patient in the opinion of the investigator.

The patient has the right to stop study treatment or completely withdraw from the study at any time and for any reason.

All patients discontinuing study treatment should have a final visit including at least all safety assessments (see also section 6.3). Reasons for patient withdrawal will be documented in the CRF.

Patients withdrawn or stopping treatment after first administration of study medication will not be replaced. Patients withdrawn prior to first administration of study medication will be replaced.

### Pausing and premature termination of the study

In case of safety or tolerability concerns, the sponsor may decide to temporarily pause recruitment of patients in the trial and/or study drug administration. When the trial is temporarily paused, the Competent Authorities (CA) and Ethics Committee will be notified immediately (but at least within 15 days from when the trial is temporarily paused). The notification will be made as a substantial amendment using the Notification of Amendment form and clearly explaining what has been halted (e.g. stopping recruitment and/or interrupting treatment of patients already included) and the reasons for the temporary pause.

Recruitment and study drug administration would resume only if review of the adverse events that caused the pause resulted in a recommendation from the review committee to permit continuation of the study. To restart a trial that has been temporarily halted, the sponsor will make the request as a substantial amendment using the Notification of Amendment form and by providing evidence that it is safe to restart the trial.

If the clinical observations in the study suggest that it may be unwise to continue, the study may be prematurely terminated by the sponsor. If the sponsor decides not to recommence a temporarily halted trial, the CA and Ethics Committees will be notified within 15 days of his decision, using the End of Trial Declaration form and including a brief explanation of the reasons for ending the trial. Additionally, the trial may be terminated by the sponsor at any time for administrative reasons.

Delta Crystallon BV. may stop the study at a specific center for any of the following reasons:

- The center cannot include an adequate number of patients
- Serious and/or persistent non-compliance with the protocol
- Careless or premeditated false documentation in the CRF
- Inadequate co-operation with Delta Crystallon BV or its representatives
- Non-compliance with GCP and / or regulatory requirements
- The investigator requests discontinuation

Delta Crystallon BV has the right to close this study at any time, although this should occur only after mutual consultation. Should the study be closed prematurely, all study materials (for example completed, partially completed and blank CRFs and all unused investigational products) must be returned to Delta Crystallon BV.

Reasons that may require termination of the study include, but are not limited to:

- Enrolment of patients is unsatisfactory
- Quality of data is unsatisfactory
- Data recording is inaccurate and / or incomplete on a chronic basis
- The incidence and / or severity of AEs indicate a potential health hazard caused by treatment with the investigational products
- Completed accrual and follow-up of patients

# Treatments

## Treatments administered

The pharmacy or an unblinded nurse not involved in clinical conduct of the study will prepare all study medication for all patients according to the treatment randomization list.

Patients will receive a single dose of study medication as intravenous bolus injection administered in the arm every two months, for a total of three times during the study. The required amount of study medication will be administered over 30 seconds to the patient via an intravenous catheter.

## Selection of doses in the study

Based on Phase I data on the T-cell tolerizing effect of a single intravenous administration of DC-TAB, the effective dose in MS patients is expected to be approximately 0.2 mg/kg, or a fixed dose of approximately 12.5 mg per injection.

The NOAEL observed in rats following 28 days treatment was set at 17.5 mg/kg, which is equivalent to an HED of 3.2 mg/kg.

The NOAEL in female dogs could not be established following 15 days of treatment, whereas the NOAEL established for male dogs was 0.5 mg/kg following 15 consecutive dose administrations. The NOAEL for up to 5 days treatment was set at 12 mg/kg for both female and male dogs.

In healthy volunteers, single doses as well as three consecutive daily intravenous doses up to 37.5 mg were well tolerated and safe. Most commonly noted adverse drug reactions were local irritability and thrombophlebitis at the injection site.

In healthy volunteers, a single dose of 12.5 mg induced a statistically significant suppression of CD4+ and CD45RO+ T-cell responses to DC-TAB, in half the subjects to levels considered to reflect non-responsiveness. This immunological effect is expected to provide a clinical effect in MS patients. Suppression of T-cells was established within 8 days and maintained for 28 days, but had partially reversed after 6-8 months. In addition to the selection of 12.5 mg, one dose level above 12.5 mg (i.e. 17.5 mg) and one dose level below 12.5 mg (i.e. 7.5 mg) was selected for this study to further obtain information on the optimal dose. Given the currently information on the half-life of inducible regulatory T cells, the tolerizing effect is expected to be maintained for approximately 2-3 months after dosing. Therefore, dosing is repeated twice with 2-month intervals.

## Methods of assigning patients to treatment groups

Apart from his/her patient screening number, each patient whose eligibility has been confirmed will be allocated a sequential randomization number at site level. Patients will be assigned to a treatment code prepared by the CRO.

## Blinding procedures

Sealed treatment randomization codes should be stored in the Investigator’s File or any other secure place at the investigator’s site.

Unblinding of the codes should only be done after closing the database, or in case of emergency. Premature unblinding should always be documented and explained on the code envelope.

At regular intervals and after completion of the study, the code envelopes will be checked by the monitor for intactness.

The syringe will be prepared by the pharmacist or an unblinded nurse who is not involved in the clinical conduct of the study. In order to maintain blinding, syringes will be covered in non-transparant foil to avoid identification of the compound based on a slight difference in colour between the active IMP and placebo. The labels in the syringes will contain the randomization number, the patients’ initials, and the date and time of preparation of the syringe.

## Treatment compliance

Administration of study medication and any deviation thereof will be recorded in the CRF.

# Conduct of the study

## Screening and selection of patients

The patient will receive verbal and written information concerning the study. The patient should be given adequate time to read the information leaflet and an opportunity to ask the investigator/delegate any questions.

Following the provision of patient information, written consent will be obtained from the patient prior to any study-specific procedure. The patient is not to enter the study if he/she has not understood the written and verbal information provided, and/or has not personally signed and dated the consent form. A copy of the patient information leaflet and the signed informed consent will be provided to the patient and a copy will be retained by the investigator.

Patients who fulfill the selection criteria will be enrolled into the study and enter a screening period to fully assess their suitability for treatment (randomization). The patients will be allocated screening number at enrolment. During this period, patients will be screened according to the inclusion and exclusion criteria and the schedule of assessments (see sections 4.4.1, 4.4.2 and 6.5).

If the assessments at screening do not reveal any condition inconsistent with the inclusion, or consistent with the exclusion criteria, the patient qualifies for treatment. Subsequently the patient will be assigned a randomization number in addition to the patient’s screening number*,* which will ensure unambiguous identification throughout the study.

Patient randomization numbers, in addition to the screening numbers assigned by the center, will be recorded on a patient log, which will be kept on file at the study-site.

## Treatment period (week 0-24)

Patients will be hospitalized the evening or the morning before study drug administration and will be discharged from the hospital 24 hours after dosing.

During hospitalization, patients will undergo a series of assessments (see schedule of assessments, section 6.5). The last available values measured prior to first study drug administration will be considered baseline measurements against which measurements during the treatment period will be compared.

Patients are to refrain from exercise other than the normal daily activities for 24 hours after injection of study medication.

On dosing days study drug administration will take place in the morning upon completion of all necessary pre-dose assessments. The following assessments will be performed: vital signs, ECG, safety biochemistry and haematology, urinalysis and biomarkers. In addition, blood sampling for PK will be performed on the first dosing day. Twenty-four hours after the study drug administration, AEs will be assessed and the patient will be discharged if in good general health. Hereafter the patients will leave the unit.

Patients will return to the hospital for ambulatory visits or hospitalization for the treatment administration visits according to the schedule of assessments, section 6.5.

## Follow-up period (week 24-week 48)

During the follow-up period, patients will return to the hospital for ambulatory visits twice to undergo the assessments according to the schedule of assessments, section 6.5.

The last available values measured prior to first study drug administration will be considered baseline measurements against which measurements during the follow-up period will be compared.

## Early Termination

In case of any early termination from the study treatment, the patient will be asked to continue scheduled evaluations, complete an end-of-study evaluation, and be given appropriate care under medical supervision until the symptoms of any adverse event resolve, or the patient’s condition becomes stable. In case of complete withdrawal from the study, all reasonable efforts should be undertaken to obtain the assessments as specified for the week 24 visit in the schedule of assessments.

## Schedule of assessments

Table 6‑1 Schedule of assessments

| **Assessment** | **Week -4 to -1 (screening)** | **Day 0** | **Week 1**  **± 1 d** | **Week 2**  **±1 d** | **Week 4**  **± 3 d** | **Week 8**  **± 3 d** | **Week 12**  **± 3 d** | **Week 16**  **± 3 d** | **Week 20**  **± 3 d** | **Week 24 or EoT**  **± 3 d** | **Week 36**  **± 3 d** | **Week 48**  **± 3 d** |
| --- | --- | --- | --- | --- | --- | --- | --- | --- | --- | --- | --- | --- |
| Written consent | x |  |  |  |  |  |  |  |  |  |  |  |
| Inclusion/Exclusion criteria | x |  |  |  |  |  |  |  |  |  |  |  |
| Demographic data | x |  |  |  |  |  |  |  |  |  |  |  |
| Medical History | x |  |  |  |  |  |  |  |  |  |  |  |
| Previous medication | x |  |  |  |  |  |  |  |  |  |  |  |
| Study drug administration^4^ |  | x |  |  |  | x |  | x |  |  |  |  |
| MRI | x |  |  | x | x | x^3^ | x | x^3^ | x | x | x | x |
| EDSS | x | x |  |  |  | x |  | x |  | x | x | x |
| MSIS-29 |  | x |  |  |  | x |  | x |  | x | x | x |
| Neurological evaluation/clinical relapse | x | x | x | x | x | x | x | x | x | x | x | x |
| Blood sample for effect parameters (T-cell response | x | x |  |  | x | x | x | x | x | x | x | x |
| Anti-CRYAB/DC-TAB antibodies | x | x |  |  | x | x | x | x | x | x | x | x |
| Safety Laboratory Tests (Haematology, Biochemistry, Urinalysis) ^6^ | x | x | x |  | x | x |  | x |  | x | x | x |
| Urine Pregnancy Test | x |  |  |  |  | x |  | x |  | x |  |  |
| PK sampling | x^8^ | X^1^ |  |  |  |  |  |  |  |  |  |  |
| Physical examination | x | x | x | x^2^ | x^2^ | x | x^2^ | x | x^2^ | x | x^2^ | x |
| Vital Signs ^7^ | x | x |  |  |  | x |  | x |  | x |  | x |
| Body temperature^7^ | x | x |  |  |  | x |  | x |  |  |  |  |
| 12-Lead ECG | x | x |  |  |  | x |  | x |  | x |  | x |
| Adverse events |  | X^5^ | x | x | x | x^5^ | x | x^5^ | x | x | x | x |
| Concomitant medication |  | x | x | x | x | x | x | x | x | x | x | x |

^1^ Samples (relative to study drug administration) at pre-dose, t=10 minutes, t=20 minutes, t=30 minutes, t=1 hour, t=2 hours, t=4 hours, t=8 hours, t=16 hours, t=24 hours;

^2^ Brief physical examination (symptom guided)

^3^ MRI can be made on dosing days after study drug administration

^4^ On study drug administration visits need patient hospitalization for 24h after dosing.

^5^ Repeated adverse event review is needed 24 hours after each dosing

^6^ See the full list of the laboratory parameters in section 7.2.1.

^7^ On dosing days blood pressure, pulse rate and temperature will be measured before and 1 hour after dosing; respiration rate only pre-dose.

All assessments are to be done prior to study drug administration unless otherwise stated.

^8^ Ten patients only; one sample collected to further investigate the stability of blood samples

## Compliance with the protocol

Any deviation from the protocol must be explained. Any patient who is entered into the study but is found not to meet the protocol entry criteria will be considered for withdrawal as a protocol violator after consultation with the sponsor.

# Methodology of assessments

## Baseline variables

### Demographics

At screening, the following demographic data will be collected: gender, date of birth, race, body weight, height and smoking habit.

### Medical history

Complete medical history will be reviewed at screening. This will include evaluation for past and present cardiovascular, respiratory, gastrointestinal, renal, hepatic, neurological, endocrine, lymphatic, hematological, immunological, dermatological, psychiatric, genitourinary, and surgical history and rhinoconjunctivitis history and any other diseases or disorders.

### Previous Medication

The investigator should record the use of all medications used within 3 months prior to entry in the study, both prescribed and over-the-counter, on the CRF. This includes drugs used on a chronic and as-needed basis. The following will be recorded on the CRF: Drug name (trade name), total daily dose, route, start and end date and time, whether ongoing and indication.

All previous medication will be coded using the World Health Organization’s Drug Dictionary (WHO-DD).

### McDonald criteria

Diagnosis at baseline is established using the Revised McDonald criteria (Polman et al. 2011).

| **Clinical Presentation** | **Additional Data Needed** |
| --- | --- |
| - 2 or more attacks (relapses) - 2 or more objective clinical lesions | None; clinical evidence will suffice  (additional evidence desirable but must be consistent with MS) |
| - 2 or more attacks - 1 objective clinical lesion | Dissemination in space, demonstrated by:   - MRI - or a positive CSF and 2 or more MRI lesions consistent with MS - or further clinical attack involving different site |
| - 1 attack - 2 or more objective clinical lesions | Dissemination in time, demonstrated by:   - MRI - or second clinical attack |
| - 1 attack - 1 objective clinical lesion   (monosymptomatic presentation) | Dissemination in space by demonstrated by:   - MRI - or positive CSF and 2 or more MRI lesions consistent with MS   ***and***  Dissemination in time demonstrated by:   - MRI - or second clinical attack |
| Insidious neurological progression  suggestive of MS  (primary progressive MS) | Positive CSF  ***and***  Dissemination in space demonstrated by:   - MRI evidence of 9 or more T2 brain lesions - or 2 or more spinal cord lesions - or 4-8 brain and 1 spinal cord lesion - or positive VEP with 4-8 MRI lesions - or positive VEP with <4 brain lesions plus 1 spinal cord lesion   ***and***  Dissemination in time demonstrated by:   - MRI - or continued progression for 1 year |

## Safety variables

The safety and tolerability of the investigational products will be evaluated by monitoring the patient’s AE profile and local tolerability throughout the study, the assessment of routine safety laboratory tests, physical examination, vital signs and 12-lead ECG.

### Safety laboratory Parameters

Blood (approximately 8 ml of blood per visit) and urine specimens for safety laboratory testing will be taken according to the schedule of assessments (see section 6.5). The laboratory tests will be coordinated / performed by the local laboratory.

Hematology will include hemoglobin (Hb), hematocrit (Hct), red blood cell count (RBC), platelet count, white blood cell count (WBC) and absolute differential count (neutrophils, basophils, eosinophils, monocytes, and lymphocytes).

Biochemistry will include alanine aminotransferase (ALT), aspartate aminotransferase (AST), alkaline phosphatase (ALP), total bilirubin, gamma glutamyl transferase (GGT), lactate dehydrogenase (LDH), total protein, albumin, urea, creatinine, sodium, potassium and chloride. In addition, eGRF will be calculated using the MDRD formula (GFR = 186.3 x SerumCr^-1.154^ * age^-0.203^ * 1.212 (if patient is black) * 0.742 (if female); an MDRD calculator can be found on MDcalc.com).

Urinalysis (semi-quantitative analysis per dipstick) will include protein, glucose, nitrite, pH, ketones, and blood. If protein, nitrite or blood is abnormal in the urine sample, microscopy will be done.

### Urine pregnancy tests

Urine pregnancy tests will be performed on all females according to the schedule of assessments (see section 6.5). Pregnancy tests will be performed at the study centre. In the event that the patient is withdrawn, a urine pregnancy test will be performed at the last visit.

### Physical Examination

Physical examinations will be performed at all visits. The examinations will include the following: general appearance, HEENT (head, ears, eyes, nose and throat), skin, cardiovascular system, respiratory system, gastrointestinal system, nervous system and other. At the visits of week 2, 4, 12, 20 and 36 a brief, symptom guided physical examination will be performed, if applicable.

### Vital Signs

Blood pressure (systolic (SBP) and diastolic (DBP)), pulse rate and respiration rate after 10 minutes supine rest will be recorded on each dosing days and at weeks 36 and 48. On dosing days, evaluations will be done pre-dose and, in addition, blood pressure and pulse rate will be assessed at 1 hour after dosing. Automatic or manual devices may be used. All devices must have been calibrated in the previous 12 months. Calibration certificates must be available on request.

### Body temperature

Body temperature will be measured at each dosing visit. On dosing days, body temperature will be measured pre-dose and at 1 hour after dosing.

### 12-Lead Electrocardiogram

12-lead ECG recordings will be obtained according to the schedule of assessments prior to dosing, if applicable (see section 6.5). The following ECG parameters will be recorded: heart rate, RR interval, PR interval, QRS complex interval and QT interval. In addition, QTc will be calculated using the Bazett and Fridericia formula by the Data Management department of PSI. A clinical assessment of “Normal”, “Abnormal, not clinically significant” or “Abnormal, clinically significant” will be made.

### Adverse Events including local and systemic reactions

The investigator is responsible for recording all AEs, including any local and systemic reactions, observed during the study. Persisting reactions should be evaluated by the investigator and the investigational product could be discontinued if indicated. See section 11.2 for detailed information on AE collection.

### Concomitant medication/antidotes

The investigator should record the use of all concomitant medications, both prescribed and over-the-counter, on the CRF. This includes drugs used on a chronic and as-needed basis. The following will be recorded on the CRF: Drug name (trade name), total daily dose, route, start and end date and time, whether ongoing and indication.

All concomitant medication will be coded using the World Health Organization’s Drug Dictionary (WHO-DD).

## Pharmacokinetics

4 mL blood samples will be taken for assessment of serum concentrations levels of DC-TAB at pre-dose, t=10 minutes, t=20 minutes, t=30 minutes, t=1 hour, t=2 hours, t=4 hours, t=8 hours, t=16 hours and t=24 hours relative to dosing on day 1. Within 2 hours after blood sampling, blood will be centrifuged at 2,000 g for 20 minutes at 4°C. In case a cooled centrifuge is not available, the rotor heads or the centrifuge may be cooled to around 4°C prior to centrifuging. Immediately after centrifugation, serum will be stored in two labelled polypropylene tubes at ≤ -20°C for serum concentration analysis. All serum concentration analyses will be performed according to GLP after the study has been finalized.

DC-TAB in serum samples, stored frozen at -20°C, has been examined for structural stability by informal non-GLP studies, using detectability by a monoclonal antibody in a sandwich ELISA as parameters for integrity. DC-TAB was found to be fully stable under such conditions for a period of at least 6 months.

In ten patients a sample will also be collected at baseline to further investigate the stability of the samples.

## Effect variables

### T-cell response

Two 10 mL blood samples will be taken for assessment of T-cell responses (CD4+ and CD45RO+) to DC-TAB and to the unrelated control antigen tetanus toxoid. Peripheral blood samples are to be collected in heparin tubes to prevent blood clotting, and kept at room temperature, also during transport, until they are processed. Samples should be shipped to the regional laboratory by courier, ensuring delivery preferably within 4 h after sampling, but no later than 6 h after sampling.

T-cell responsiveness assays will be initiated on the day of sample collection. Peripheral blood mononuclear cells (PBMC) will be isolated from blood samples within 8 h after sampling, and immediately subjected to an assay for antigen-specific proliferation. The cell culture assay to determine antigen-specific responses of T cells to either DC-TAB itself, or to tetanus toxoid as a control antigen Peripheral mononuclear cells will be isolated from blood samples, labeled with the fluorescent dye carboxyfluorescein succinimidyl ester (CFSE), and placed in culture with antigen at doses known to stimulate maximum proliferative responses. After 9 days, cells will be harvested, additionally stained with fluorescently labeled antibodies to either CD4 (a marker for helper T cells) or CD45RO (a marker for memory T cells of both the CD4 and CD8 lineage), and subjected to flowcytometry. During flowcytometry, cells will be scored for levels of the original CFSE label, and levels of the additional CD4 or CD45RO marker. Since the CFSE label in a mother cell is equally distributed over its resulting daughter cells, proliferated cells are typified by reduced levels of CFSE, especially after several rounds of cell division. Flowcytometry thus allows quantitation of the fraction of either CD4 or CD45RO cells which have proliferated in culture in response to antigen. All data points will be collected at least in triplicate, with each data point being collected using the combined cells of six individual culture wells.

T-cell responses will be assessed by the Central Reference Laboratory for Immunology, at the National Center for Infectious and Parasitic diseases, Sofia, Bulgaria.

### Antibody assessment

To determine antibody levels to CRYAB/DC-TAB, two 1-mL plasma samples will be collected in the course of processing the blood samples which are already drawn for measuring T-cell responses. When peripheral blood mononuclear cells are isolated from the blood samples for the T-cell assay, as described above, two 1-mL plasma samples will become available during the process. These are collected and stored frozen at -20°C. Samples will be shipped for analysis to Delta Crystallon batch-wise every 6 months.

Antibody assays include a quantitation of total serum IgG titers against CRYAB/DC-TAB using a standard ELISA set up, and a bioactivity assay to evaluate neutralizing activity of serum antibodies. This activity will be evaluated by examining the inhibitory effects of serum dilutions on the ability of DC-TAB to induce activation of a specific reporter cell line in culture.

All antibody assessments will be performed by Delta Crystallon BV, Leiden, the Netherlands.

### MRI

Gadolinium brain MRI will be performed at the visits indicated in the schedule of assessment (section 6.5).

A detailed MRI scanning protocol including but not limited to field strength, slice thickness and gap, pixel size, brain coverage, core brain sequences, gadolinium dose and infusion speed and time between gadolinium dosing and post-dose imaging will be prepared prior to start of the study.

All MRI readings will be performed centrally by the VU Medical Center, Amsterdam, The Netherlands.

### Clinical relapse

A confirmed clinical relapse is defined as the occurrence of new symptoms or worsening of previously stable or improving symptoms and signs not associated with fever, lasting more than 24 hours and accompanied by an increase of at least half a point in the EDSS score or 1 point in the score for at least one of the functional systems (excluding the bowel and bladder and mental systems).

Neurologic deterioration that is classified by the treating physician as a relapse but that does not fulfil these criteria will be documented as an unconfirmed clinical relapse.

Clinical relapses may be treated with systemic corticosteroids if deemed in the best interest of the patient. Dose and duration of treatment is at the discretion of the investigator. In case the clinical relapse coincides with the next study drug administration, the investigator will consult with the sponsor’s responsible medical person to determine if and when the next dose may be given.

### EDSS

The Kurtzke Expanded Disability Status Scale (EDSS) is used to quantify disability in multiple sclerosis.

The following functional systems will be assessed and scored according to the EDSS scale (refer to Appendix one for the overall assessment of the EDSS):

- Pyramidal (ability to walk)
- Cerebellar (coordination)
- Brain stem (speech and swallowing)
- Sensory (touch and pain)
- Bowel and bladder functions
- Visual
- Mental
- Other (includes any other neurological findings due to MS)

### MSIS-29

The patient will be asked to fill out the MSIS-29 version 2 questionnaire at the visits indicated in the schedule of assessments (section 6.5) to assess the impact of multiple sclerosis on daily life.

# Study medication

## Identity of the investigational products

Investigational product

Product: DC-TAB

Formulation: solution for intravenous injection

Strength: 12.5 mg/ml

Vial volume: 1 ml

Placebo

Product: PBS

Formulation: solution for intravenous injection

## Drug manufacturing

The study medication is manufactured under supervision of the sponsor by Novozymes, Sweden. Manufacturing operations are in accordance with Good Manufacturing Practice.

All medication used in this study will be prepared and labeled according to the rules of Good Manufacturing Practice, ICH-GCP and local regulatory requirements.

Study medication labeling contains the following items:

- Study number
- Product identification
- Concentration and volume
- Route of administration
- Expiry date
- Batch number
- Sponsor address and telephone number

## Drug storage and stability

The investigational product is to be stored frozen at -20ºC.

Stability studies are currently ongoing. Previous batches indicated that DC-TAB is stable at -20º ± 5º C for at least 66 months, and at 25 ± 2ºC for at least 24 months. DC-TAB can be thawed and re-frozen for storage at -20ºC at least five times.

## Preparation, administration and dosage of treatment

DC-TAB is provided in ready-to-use 1 ml vials containing 12.5 mg/ml DC-TAB. The pharmacy of the unit of an unblinded nurse not involved in the clinical conduct of the study will prepare the syringes according to the treatment assigned to each individual patient (i.e. 7.5, 12.5 and 17.5 mg). PBS will be added until a total volume of 10 ml is obtained (to limit the residual volume of investigational product in the syringe and needle).

Placebo (PBS) will be sourced from a commercial supplier. 10 ml PBS will be prepared in a syringe. In order to maintain the blind, all study drug syringes will be covered using non-transparent foil.

Syringes will be labeled with the patient’s randomization number, patient’s initials, and date and time of preparation of the syringe. After preparation syringes will be stored between +2 and +8ºC in a refrigerator. In order to maintain the blind, the syringes will be covered using non-transparant foil.

Patients will receive the study medication within three hours after preparation as a bolus injection in the arm. Following injection, the intravenous catheter will be flushed with 10 ml PBS.

## Drug accountability

The sponsor or designate will request the investigator or responsible pharmacist to sign a receipt for the study drugs. All study medication supplied for the study should be kept in a locked secure place with appropriate pharmaceutical precautions.

A “Drug Accountability” record should be maintained by the person responsible for dispensing the trial medication to the patient. This should record which supplies are issued to which patients and any drugs returned unused. Details of any supplies that are inadvertently damaged should be given on this record, which will be collected by the monitor at the end of the study.

All unused study medication should be kept and added to the drug accountability record. All study medication in these categories will be inventoried by an unblinded monitor not involved with other monitoring tasks during and at the conclusion of the study. The monitor will arrange for their secure disposal at the end of the study.

The drugs supplied for this study are only intended for use by patients in this study. They must not be diverted for use by others.

# Data handling and record keeping

## Data collection

Data collection is the responsibility of the clinical trial staff at the site under the supervision of the Principal Investigator. During the study, the Investigator must maintain complete and accurate documentation for the study.

Conducting a trial and the related use of CRFs should not detract from the routine data recording in the source documents. It should be clearly marked in the medical records of the patient at the trial site that the patient is participating in a particular clinical trial. Source data must be available to document the existence of the patient and substantiate integrity of trial data collected. Source data must include the original documents related to the trial (e.g. ECG traces, laboratory prints), to the medical treatment and to the history of the patient.

All data obtained in the clinical trial described in this protocol will be transcribed to Case Report Forms (CRFs). All missing data will be explained. CRF items not done will be marked as “ND.” If an item is unknown or not applicable to the specified case, the space in the CRF will be marked “UNK” and “NA”, respectively. Data that has been collected but cannot be retrieved will also be marked “UNK.” All data entries will be made in permanent, black ink.

The CRFs for the recording of trial data for each patient will be presented in a booklet or bundle.

Each CRF must be filled in neatly with a ballpoint pen only. The final authorization of the CRF is considered to be the "End Of Trial" (EOT) form. The PI signs the EOT form to signify review of the booklet and EOT form and to certify them to be complete and accurate.

At each visit of the monitor, the CRFs should be available for review.

The following information should be included in the source medical records:

- demographic data
- medical history
- full physical examination details
- adverse events and concurrent treatment(s)
- ECG traces
- visit dates and dispensing of study medication
- information on the patient’s treatment
- hematology, biochemistry and urinalysis results
- MSIS-29 questionnaire
- EDSS scale

The following information may be recorded directly in the CRF and should be considered as source data:

- vital signs
- body temperature
- brief physical examination conduct
- recording of dates and times of blood sample drawings

## Data management

Data management will be performed by PSI Ltd., Russia.

The completed CRF will be submitted to the clinical data management department of PSI. The data entry will be carried out using ClinTrial^TM^, version 4.7. All data collected will be entered in the database. Two different operators will enter data in duplicate, and control checks between the two databases will be done until 100% concordance is reached.

Queries will be issued, e.g. on missing data, inconsistencies, illegible data, illegal values and unclearly corrected items. Resolution of queries will be implemented in the database. Certain (obvious) errors may be resolved by the assigned data manager in accordance with PSI SOP’s. All other errors will be queried by the data manager and resolved by the investigator.

Errors in the CRF must be corrected by drawing a single thin line with ballpoint pen only, through the incorrect entry and by writing in the new value as close to the original one as possible. Corrections are to be initialed and dated by the person making the correction and, if deemed necessary, the reason for correction must be explained next to the correction made.

## Record keeping

The investigator will maintain a study file, which he/she should use to file the IB, protocol, drug accountability records, correspondence with the IEC/IRB, the sponsor and other study-related documents.

The investigator’s copy of the CRFs, study file, consent forms, drug accountability records and the patient identification list must be kept by the investigator for at least 15 years. These documents should be retained for a longer period, however, if required by the applicable regulatory requirements. It is the responsibility of the sponsor to inform the investigator/institution as to when the documents no longer need to be retained. In addition the investigator must make provision for the patients’ records to be kept for the same period of time.

The sponsor will archive and retain all documents pertaining to the study for at least 15 years after the last approval.

# Statistical analysis and reporting

## Study parameters

### Safety parameters

- Number and frequency of adverse events including local irritability complaints
- ECG parameters
- Clinical chemistry and hematology parameters
- Urinalysis
- Vital signs and body temperature
- MRI signs and symptoms

### Effect parameters and endpoints

Effect parameters

- - Proliferative CD45RO+ (memory) and CD4+ (helper) T-cell response to DC-TAB
  - New or enlarging gadolinium-enhancing lesions on T1-weighted MRI
  - Clinical relapse
  - EDSS
  - MSIS-29
  - Levels of serum antibodies against CRYAB/DC-TAB

Exploratory effect endpoints

- Change in T-cell responses (CD4+ and CD45RO+) to DC-TAB throughout the study
- Cumulative number of new or enlarging gadolinium-enhancing MRI lesions between week 4 and 24, and week 4 and 48 first dose, relative to baseline
- The number of clinical relapses between week 0 and week 24, and week 0 and week 48
- Change in EDSS score between week 0 and week 24, and week 0 and week 48
- Change in MSIS-29 score between week 0 and week 24, and week 0 and week 48
- Change in levels of CRYAB/DC-TAB-reactive serum antibodies
- Change in neutralizing activity of CRYAB-reactive serum antibodies

### Pharmacokinetic parameters

- AUC_0-24h_
- AUC_0-∞_
- C_max_
- t _1/2_
- t _max_
- Kel

## Evaluability of data

All patients who received at least one dose of study medication will be included in the safety analysis.

## Statistical analysis

Statistical analysis will be performed by PSI. A statistical analysis plan will be prepared prior data base lock describing the data analysis in more detail. Deviations from the Statistical Analyses Plan will be justified in the study report. All statistical calculations will be performed using SAS version 9.

A statistical analysis plan will be prepared prior to database lock describing the data analysis in more detail.

### Handling of Missing and Incomplete Data

Handling of missing and incomplete data will be detailed in the statistical analysis plan.

### Safety data

Individual and summary blood pressures, heart rate, respiration rate, body temperature and ECG parameters will be presented using descriptive statistics which include tabular form with mean, median, standard deviation and range (min and max) as appropriate.

Adverse events will be coded by SOC and preferred term according to the current version of the Medical Dictionary for Regulatory Activities (MedDRA). Adverse events will be summarized by number of patients and percentage of patients by treatment group, MedDRA SOC and Preferred Term. Serious AEs, drug-related AEs and serious drug-related AEs will also be summarized.

### Pharmacokinetic data

Plasma concentrations will be listed and summarized by nominal time point, and treatment (number of patients (N), mean, standard deviation, minimum, median maximum, coefficient of variation). Individual and mean plasma concentration vs. time profile plots will be produced for each dose level.

All pharmacokinetic calculations will be done using WinNonlin (version 5.2 or higher). Pharmacokinetic parameters will be listed and summarized by treatment.

Statistical analyses of pharmacokinetic data will include enrolled patients who receive study treatment and complete the study. The AUC_last_, AUC_inf_ and C_max_ parameters will be analyzed both prior to and after natural log transformation, as applicable. The pharmacokinetic parameter estimates will be evaluated by Analysis of Variance.

Details will be described in the statistical analysis plan.

### Effect data

Continuous variables will be summarized descriptively providing, where applicable, the number of patients (N), mean, standard deviation (SD), coefficient of variation, median, minimum (min) and maximum (max).

Changes from baseline will be summarized descriptively providing, where applicable, the number of patients (N), mean, standard deviation (SD), median, minimum (min) and maximum (max).

A Student’s T-test will be performed to analyze the changes in the absolute percentage of proliferated T-cells as well as the cell division index over time.

Details on the statistical analysis methods used will be further detailed in the statistical analysis plan.

### Demographic and background variables

Baseline demographic and background variables will be summarized for all patients. For categorical variables, frequencies and percentages will be provided. For continuous variables, descriptive statistics including the sample size, mean, median, standard deviation and range, will be presented.

### Patient accountability

All patients enrolled will be included in a summary of patient accountability. The frequency and percentage of patients enrolled in the study, randomized, discontinued before completing the study, and completing the study will be summarized.

### Study medication compliance

A summary of study medication usage and compliance will be provided for all patients by treatment group.

## Blinded Safety Review Analysis

Three blinded safety review analyses will be performed, after 12 patients have completed the visits at week 4 and 12 and after 24 patients have completed the visit at12 weeks.

An independent DSMB will be constituted to evaluate the blinded safety data. Data of patients with serious adverse events may be unblinded at the request of the DSMB. If the safety evaluation by the DSMB reveals any investigational product-related serious adverse events, the corresponding dose regimen may be discontinued.

## Sample size justification

The number of patients to be included is based on medical rather than statistical grounds. It is considered that the foreseen number of patients per dose group will provide sufficient safety information and information on the effect of DC-TAB to start a larger Phase 2b study in this patient population.

## Study report

At the conclusion of the study, after the data are analyzed, the sponsor will prepare an integrated study report in accordance with ICH-E3. A draft copy of the report will be available for review by the PI. The report will be signed by the PI and appropriate persons from the sponsor.

# Adverse events

## Adverse event definition

An adverse event is any untoward medical occurrence in a patient or clinical investigation patient administered a pharmaceutical product and which does not necessarily have a causal relationship with this treatment. An adverse event (AE) can therefore be any unfavourable and unintended sign (including a laboratory finding), symptom or disease temporarily associated with the use of a medicinal product, whether or not related to the medicinal product.

Adverse events may include, but are not limited to:

- Subjective or objective symptoms spontaneously offered by the patient, parent/legal representative or observed by the investigator or medical staff.
- Laboratory abnormalities of clinical significance.

Disease signs, symptoms and/or laboratory abnormalities already existing prior to the use of the product are not considered adverse events after treatment unless they reoccur after the patient has recovered from the pre-existing condition or in the opinion of the investigator they represent a clinically significant exacerbation in intensity or frequency.

## Reporting adverse events

At each evaluation, the investigator will determine whether any adverse events have occurred. If any adverse events have occurred, they will be recorded on the adverse event report pages of the CRF. If known, the diagnosis should be recorded, in preference to the listing of individual signs and symptoms.

Any adverse event experienced by the patient from the time of signing the informed consent until the end of the study will be recorded in the CRF.

The investigator will be asked to assess the severity of the adverse drug/ biologic event using the following categories: mild, moderate and severe. This assessment is subjective and the investigator should use medical judgment to compare the reported adverse event to similar type events observed in clinical practice. Below are listed guidelines for severity assessment:

Mild: Symptom(s) barely noticeable to the patient or does not make the patient uncomfortable. The adverse event does not influence performance or functioning. Prescription drugs are not ordinarily needed for relief of symptom(s).

Moderate: Symptom(s) of a sufficient severity to make the patient uncomfortable. Performance of daily activities is influenced. Treatment of symptom(s) may be needed.

Severe: Symptom(s) of a sufficient severity to cause the patient severe discomfort. Severity may cause cessation of treatment with the study drug. Treatment for symptom(s) may be given.

The investigator will make a judgment regarding whether or not, in his/her opinion, the adverse event was related to study drug. The investigator will also evaluate any changes in laboratory values, make a determination as to whether the change is clinically important, and whether or not the changes were related to study drug. However, even if the investigator feels there is no relationship to the study drug, the adverse event or laboratory abnormality must be recorded in the CRF.

If the relationship between the study drug and the adverse event is defined as “not related”, then the adverse event must definitely be caused by the patient’s clinical state, or the study procedure/conditions.

A “remote/unlikely” relationship is defined as when the temporal association between the adverse event and the drug is such that the drug is not likely to have any reasonable association with the adverse event.

A “possible” relationship is defined as when an adverse event could have been produced by the patient’s clinical state or the therapy.

A “probable” relationship is defined as when the adverse event follows a reasonable temporal sequence from the time of drug administration, abates upon discontinuation of the drug and cannot be reasonably explained by the known characteristics of the patient’s clinical state.

A “definite” relationship is defined as when the adverse event follows a reasonable temporal sequence from the time of drug administration, abates upon discontinuation of the drug and reappears when the drug is introduced.

## Reporting of Pregnancy

A newly diagnosed pregnancy in itself will not be considered an AE unless it is suspected that the investigational product(s) interacted with a contraceptive method or had some association with the occurrence of pregnancy. A congenital anomaly as a result of this pregnancy is a serious adverse event (SAE).

Before study enrollment, women of childbearing potential must be advized of the importance of avoiding pregnancy during their study participation. All women of childbearing potential must be willing to undergo a pregnancy test before the first administration of the investigational product.

The investigator must immediately notify the sponsor of any pregnancy associated with the study exposure, including 30 days for females after the study who will forward Pregnancy Surveillance Form(s) for monitoring the outcome of the pregnancy.

In addition, the investigator must report to the sponsor follow-up information regarding the course of the pregnancy, including perinatal and neonatal outcome. Infants will be followed for a minimum of 6 months.

## Follow-up of adverse events

Adverse events will be monitored from the time of ICF signature. Treament emergent adverse events will be monitored from the time the first study treatment is administered to the end of the study. Adverse events will be elicited by direct, non-leading questioning or by spontaneous reports. At each visit, the patient should be asked a non-leading question such as: “Do you feel different in any way since starting the last assessment?” Adverse events already recorded on a previous occasion, and designated as ‘continuing’, should be reviewed at each subsequent assessment. Any adverse event will be followed-up until the event or its sequelae resolve or stabilize at a level acceptable to the investigator and sponsor’s clinical monitor or his/her designated representative.

# Serious adverse events

## Serious adverse event definition

A Serious Adverse Event is any untoward medical occurrence that at any dose:

- Results in death
- Is life-threatening
- Requires patient hospitalization or prolongation of existing hospitalization
- Results in persistent or significant disability/incapacity
- Is a congenital anomaly/birth defect
- Is considered medically important; e.g. requires intervention to prevent permanent impairment or damage (for studies under IND)

Medical and scientific judgment should be exercised in deciding whether expedited reporting is appropriate in other situations, such as important medical events that may not be immediately life-threatening or result in death or hospitalization but may jeopardize the patient or may require intervention to prevent one of the other outcomes listed in the definition above. These should also be considered serious.

A death occurring during the study or which comes to the attention of the investigator within 30 days of study drug administration, whether considered treatment related or not, must be reported. In the event of an SAE the investigator may immediately stop treatment if it is considered in the best interest of the patient. In case a serious adverse event related to the study medication occurs, the patient must be discontinued from treatment, unless doing so would harm the patient in the opinion of the investigator.

### Unexpected adverse drug reaction

“Unexpected adverse drug reaction” is defined as an adverse reaction, the nature or severity of which is not consistent with the Investigator’s Brochure.

### Life-threatening adverse event

Any adverse event that places the patient, in view of the reporter, at immediate risk of death from the reaction as it occurred, i.e. it does not include a reaction that, had it occurred in a more serious form, might have caused death.

### Hospitalization

This is defined as the patient being hospitalized overnight, or the patient’s hospital stay being prolonged for at least an additional night. Pre-planned hospital stays or stays for social reasons will not be considered hospitalization.

### Persistent or significant disability/incapacity

This is defined as any adverse event that resulted in a substantial disruption of a person’s ability to conduct normal life functions.

### Medical occurrence requiring intervention to prevent permanent impairment or damage

This is defined as any adverse event that may jeopardize the patient and may require medical or surgical intervention to prevent death, life threatening, (prolonged) hospitalization, disability or congenital anomaly.

## Reporting serious adverse events and/or unexpected adverse drug reactions

Investigators are obliged to notify, by fax (or telephone), to the sponsor all serious adverse events and unexpected adverse drug reactions IMMEDIATELY (within 24 hours of the investigator becoming aware of the event). The investigator will be requested to supply as much detailed information regarding the event that is available at the time of the initial contact. The investigator is also required to submit follow-up reports to the monitor until the adverse event has resolved or in the case of permanent impairment, until the adverse event stabilizes.

**ANY SERIOUS ADVERSE EVENT WHETHER OR NOT RELATED TO THE STUDY DRUG MUST BE REPORTED IMMEDIATELY TO THE FOLLOWING DEPARTMENT:**

***Global Clinical Safety & Pharmacovigilance PSI***

***PSI medical officer: Gyorgy Andor, MD***

***Telephone: +36 (1)555 6755 x6417***

***Fax (toll free numbers for Bulgaria): 0800 180 09***

***00800 800 007 23***

***Email: safetydesk@psi-cro.com***

For all SAEs a Serious Adverse Event Report Form that includes a detailed written description, anonymized copies of relevant patient records, autopsy reports, and other documents should be faxed to the above mentioned fax number, within 48 hours.

The responsible pharmacovigilance officer will immediately evaluate the SAEs for reporting to the appropriate regulatory agencies in consultation with the medical responsible person from the sponsor.

## Suspected Unexpected Serious Adverse Reaction (SUSAR) reporting

PSI is responsible for the expedited reporting of SUSAR’s to the applicable regulatory authorities and Ethics Committee. Expedited reporting means not later than 15 days after the sponsor has first knowledge of the adverse reactions. For fatal or life threatening cases the term is maximal 7 days for a preliminary report with another 8 days for completion of the report.

# Ethics and protection of study participants

## Basic principles

This study will be conducted in compliance with the protocol, ICH-GCP, the Declaration of Helsinki and all applicable regulatory requirements.

## Independent Ethics Committee/Institutional Review Board approval

Written approval must be gained from the IEC/IRB prior to release of clinical supplies and commencement of the study.

The protocol, the IB, the IMPD, the patient information sheet, the consent form, the investigator’s curriculum vitae and the advertisements (if any) will be submitted by the principal investigator(s) to the Independent Ethics Committee (IEC) or Institutional Review Board (IRB). The IEC/IRB must conform to ICH-GCP.

A copy of the written approval must be provided to the sponsor.

Any amendments to the protocol or patient information and consent form will be submitted to the IEC/IRB of the investigational center and written approval will be obtained for substantial amendments prior to implementation (see also section 14.1).

## Regulatory requirements

The study will only start in a study center after having received written approval from the relevant Regulatory Authorities.

## Insurance of the patient

The sponsor will cover this study by means of an adequate insurance of the patient which will be in place prior to the start of the study.

## Informed consent

It is the responsibility of the Investigator to obtain written Informed Consent from the patient. Information about the study will be given to the patient both verbally and in writing. The written patient information sheet will explain the objectives of the study, its potential risks and benefits. The investigator must be satisfied that the patient has understood the information provided before written consent is obtained.

It should be made clear that refusal to participate or withdrawal from the trial at any stage is without any prejudice to the patient’s subsequent care. No patient should be obliged to participate in the trial. The patient must be given ample opportunity to enquire about details of the trial. If there is any doubt as to whether the patient has understood the written and verbal information, the patient should not enter the study.

The patient must be made aware that the monitors, auditors, the IEC/IRB and regulatory authorities will be granted direct access to the study patients source medical records without violating patient confidentiality, and to the extent permitted by applicable regulations.

If the patient agrees to participate in the study he will be asked to sign and date a consent form which will be kept by the Investigator. The patient information leaflet and a copy of the signed informed consent will be provided to the patient.

The signed consent forms will be retained by the investigator and made available (for review only) to the study monitor and auditor on request.

# Study administrative procedures

## Protocol amendments

Any changes to the study, which arise after approval of the protocol, must be documented as protocol amendments. Protocol amendments affecting the safety of the patient, the scope of the study and/or the scientific quality (i.e. significant amendments) should be submitted to regulatory authorities and to the IEC/IRB for approval. The changes will become effective only after approval by the sponsor, the responsible investigator, IEC/IRB and regulatory authorities. All other amendments (i.e. non-substantial amendments) will be submitted to the IEC/IRB and regulatory authorities for notification.

## Study monitoring

The sponsor of this study is responsible according to ICH-GCP guidelines for assuring proper study conduct with regard to protocol adherence and validity of the data recorded in the CRFs.

The sponsor has therefore assigned a study monitor to this study. His/her duties are to assist the investigator in the maintenance of complete, legible, well organized, and easily retrievable data. In addition, the monitor will ensure that the investigator understands all applicable regulations concerning the clinical evaluation of an investigational drug, as laid down in ICH GCP guidelines.

The investigator agrees to allow the monitor direct access to the study drug dispensing and storage area and to all clinical data of the study patients for the above purposes and agrees to assist the monitor in these activities. The investigator accepts that the monitor will visit the center at regular intervals to review and verify the data collected. The monitor will regard all information that is supplied to him or her as strictly confidential.

## Patient confidentiality

Each participating site will maintain appropriate medical and research records for this trial, in compliance with ICH E6 GCP and local law requirements for the protection of confidentiality of patients.

Clinical research personnel assigned by the sponsor will require direct access to the patient’s source data for data verification. The confidentiality of all the patients’ identities will be maintained. Source data are all information, original records of clinical findings, observations, or other activities in a clinical trial necessary for the reconstruction and evaluation of the trial. Examples of these original documents and data records include, but are not limited to, hospital records, clinical and office charts, laboratory notes, memoranda, patients’ diaries or evaluation checklists, pharmacy dispensing records, recorded data from automated instruments, copies or transcriptions certified after verification as being accurate and complete, microfiches, photographic negatives, microfilm or magnetic media, x-rays, and patient files and records kept at the pharmacy, at the laboratories, and medico-technical departments involved in the clinical trial.

Only patients’ date of birth and study number will be used on CRFs and in all study correspondence. No material bearing a patient’s name will be kept on file by the sponsor or clinical research personnel assigned by the sponsor.

All information disclosed to the investigator by the sponsor or persons assigned by the sponsor shall be treated by the investigator as strictly confidential. The Investigator shall only use such information for the purpose of conducting the clinical trial described in this protocol, and the investigator agrees not to disclose such information to any third party except those of his colleagues and employees who are assisting in the conduct of the study and who are bound by the obligations of confidentiality.

## Use of information and publications

Information concerning the study drug, patent applications, processes, unpublished scientific data, the Investigator's Brochure and other pertinent information is confidential and remains the property of the sponsor. Details should be disclosed only to the persons involved in the approval or conduct of the study. The investigator may use this information for the purpose of the study only. It is understood by the investigator that the sponsor will use the information obtained during the clinical study in connection with the development of the drug and therefore may disclose it as required to other clinical investigators or to regulatory agencies. In order to allow for the use of the information derived from this clinical study, the investigator understands that he has an obligation to provide the sponsor with all data obtained during the study.

The study may be considered for publication or presentation at (scientific) symposia and congresses.

The investigator will be entitled to publish or disclose the results only after written approval of the manuscript by the sponsor. The sponsor will be allowed to review all transcripts, texts of presentations and abstracts related to the study at least three months prior to the intended submission for publication or any other disclosure. This is necessary to prevent premature disclosure of trade secrets or patent-protected information and is in no way intended to restrict publication of facts or opinions formulated by the investigator.

The sponsor will inform the investigator of any objection or question arising within 30 days of receipt of the proposed publication. After written approval is obtained, the manuscript is free for publication.

## Quality assurance

The sponsor may conduct (or have conducted) periodical inspections of the study by reviewing the data obtained as well as the procedural aspects. This may include on-site inspections and source data checks. Direct access to source documents is required for the purpose of these periodical inspections. Any such access will be confidential and the identity of the patient will not appear on any copies made of the records.

## Regulatory inspections

Regulatory authorities may also wish to have direct access to the medical records of patients participating in the study for the purpose of audit or inspection. Any such access will be confidential and the identity of the patient will not appear on any copies made of the records. The inspection may occur during the study, or at any time following the study.

# Study documentation and supplies

The sponsor will provide the investigator with the following documents:

- Study protocol
- Investigator's Brochure
- Study medication with all necessary documentation
- Study contract
- CRFs
- Adverse event report forms
- Patient information leaflets and informed consent forms
- Investigator’s study file
- MRI protocol

In order to begin the study, the responsible investigator is required to provide the following documentation to the sponsor:

- Signed confidentiality agreement
- Signed investigator's agreement
- Signed hospital contract
- Independent Ethics Committee approval, stating clearly the sponsor's name, study number and investigational drug, including a list with names and qualifications of all members of the Independent Ethics Committee and a list of documents reviewed.
- Recent versions of signed and dated curricula vitae of all investigators and sub investigators
- Signature sheet, documenting signatures, initials and trial related duties of all study site personnel
- Laboratory normal ranges, signed and dated by the responsible laboratory employee
- Medical/laboratory/technical procedures/tests methods of assay, certifications or accreditation’s or established quality control or other validations, where applicable.

At the end of the study, the sponsor is responsible for the collection of:

- All sponsor’s left-over study documentation
- Left-over study medication

# Reference list

1. Frohman EM, Racke MK, and Raine CS (2006) Multiple sclerosis – the plaque and its pathogenesis. *New Engl J Med* **354**, 942-955.
2. International Multiple Sclerosis Genetics Consortium (2007) Risk alleles for multiple sclerosis identified by a genomewide study. *New Engl J Med* **357,** 851-862.
3. Zaadstra BM, Chorus AMJ, Van Buuren S, Kalsbeek H, and van Noort JM (2008). Selective association of multiple sclerosis with infectious mononucleosis. *Mult Scler* **14,** 307-313.
4. De Lorenze GN, Munger KL, Lenette ET, Orentreich N, Vogelman JH and Ascherio A (2006) Epstein-Barr virus and multiple sclerosis: evidence of association from a prospective study with long-term follow-up. *Arch. Neurol.* **63,** 839-844.
5. Van Noort JM, Van Sechel AC, Bajramovic JJ, El Ouagmiri M, Polman CH, Lassmann H and Ravid R (1995) The small heat-shock protein alpha B-crystallin as candidate autoantigen in multiple sclerosis. *Nature* **375**, 798-801.
6. Sinclair C, Mirakhur M, Kirk J, Farrell M, and McQuaid S (2005) Upregulation of osteopontin and alpha B-crystallin in the normal-appearing white matter of multiple sclerosis: an immunohistochemical study utilising tissue microarrays. *Neuropathol Appl Neurobiol* **31**, 292-303.
7. Chabas D, Baranzini SE, Mitchell D, Bernard CC, Ritling SR, Denhardt DT, Sobel RA, Lock C, Karpuj M, Pedotti R, Heller R, Oksenberg JR and Steinman L (2001) The influence of the proinflammatory cytokine, osteopontin, on autoimmune demyelinating disease. *Science* **294**, 1731-1735
8. Tajouri L, Mellick AS, Ashton KJ, Tannenberg AEG, Nagra RM, Tourtelotte WW, and Griffiths LR (2003) Quantitative and qualitative changes in gene expression patterns characterise the activity of plaques in multiple sclerosis. *Mol Brain Res* 119, 170-183.
9. van Noort JM, Bsibsi M, Gerritsen WH, van der Valk P, Bajramovic JJ, Steinman L, Amor S. (2010) Alpha B-crystallin is a target for adaptive immune responses and a trigger of innate responses in preactive multiple sclerosis lesions. *J Neuropathol Exp Neurol* 69, 694-703.
10. Ousman SS, Tomooka BH, van Noort JM, Wawrousek EF, O’Conner K, Hafler DA, Sobel RA, Robinson WH and Steinman L (2007) Protective and therapeutic role for αB-crystallin in autoimmune demyelination. *Nature*, **448:** 474-479
11. Masilamoni JG, Jesudason EP, Baben B, Jabaraj CE, Dhandayuthapani S, and Jayakumar R. (2006) Molecular chaperone alpha-crystallin prevents detrimental effects of neuroinflammation. *Biochim. Biophys. Acta* **1762**, 284-293.
12. Masilamoni JG, Vignesh S, Kirubagaran R, Jesudason EP, and Jayakumar R (2005) The neuroprotective efficacy of alpha-crystallin against acute inflammation in mice. *Brain Res Bull* **67**, 235-241.
13. Masilamoni JG, Jesudason EP, Barathi SN, and Jayakumar R (2005) The protective effect of alpha-crystallin against acute inflammation in mice. *Biochim Biophys Acta* **1740**, 411-420.
14. Pangratz-Fuehrer S, Kaur K, Ousman SS, Steinman L, Liao YJ (2011) Functional rescue of experimental ischemic optic neuropathy with αB-crystallin. *Eye* **25**, 809-817.
15. Velotta JB, Kimura N, Chang SH, Chung J, Itoh S, Rothbard J, Yang PC, Steinman L, Robbins RC, Fischbein MP (2011) αB-Crystallin improves murine cardiac function and attenuates apoptosis in human endothelial cells exposed to ischemia-reperfusion. *Ann Thorac Surg* **91**, 1907-1913.
16. Van Noort JM, Verbeek R, Polman CH, Meilof J and Amor S (2006) Autoantibodies against alpha B-crystallin, a candidate autoantigen in multiple sclerosis, are part of a normal human immune repertoire. *Mult Scler* **12,** 287-293.
17. Van Sechel AC, Bajramovic JJ, Van Stipdonk MJB, Persoon-Deen C, Geutskens S, and van Noort JM (1999) Epstein-Barr virus-induced expression and HLA-DR-restricted presentation by human B cells of alpha B-crystallin, a candidate autoantigen in multiple sclerosis. *J. Immunol.* **162,**129-135.
18. Verbeek R, van Dongen H, Wawrousek EF, Amor S and van Noort JM (2007) Induction of EAE by T cells specific for alpha B-crystallin depends on prior viral infection in the CNS. *Int. Immunol*. **19:** 277-285.
19. Verbeek R, Van der Mark K, Wawrousek EF, Plomp AC and van Noort JM (2007) Tolerisation of an established alpha B-crystallin-reactive T-cell response by intravenous antigen*. Immunology* **121**: 416-426.
20. Akbar AN, Vukmanovic-Stejic M, Taams LS, Macallan DC (2007) The dynamic co-evolution of memory and regulatory CD4+ T cells in the periphery. *Nat Rev Immunol*. **7**:231-7.

**Appendix 1 EDSS scale**

**The Expanded Disability Status Scale (EDSS) – overall scoring**

0.0: Normal neurological exam.

1.0: No disability, but minimal signs in one functional system (FS) are present.

1.5: No disability, but minimal signs in more than one FS are present.

2.0: Minimal disability in one FS is present.

2.5: There is mild disability in one FS or minimal disability in two FS.

3.0: There is moderate disability in one FS or mild disability in three or four FS. However, the person is still fully ambulatory.

3.5: The person is fully ambulatory, but has moderate disability in one FS and mild disability in one or two FS; or moderate disability in two FS; or mild disability in five FS.

4.0: The person is fully ambulatory without aid, and is up and about most of the day (12 hours) despite relatively severe disability. He or she is able to walk 500 meters without aid or rest.

4.5: The person is fully ambulatory without aid, and is up and about much of day. He or she is able to work a full day, but may otherwise have some limitations of full activity or require minimal assistance. This is considered relatively severe disability. Able to walk 300 meters without aid.

5.0: The person is able to walk 200 meters without aid or rest. Disability impairs full daily activities, such as working a full day without special provisions.

5.5: The person is able to walk 100 meters without aid or rest. Disability precludes full daily activities.

6.0: The person needs intermittent or unilateral constant assistance (cane, crutch or brace) to walk 100 meters with or without resting.

6.5: The person needs constant bilateral support (cane, crutch or braces) to walk 20 meters without resting.

7.0: The person is unable to walk beyond five meters even with aid, and is essentially restricted to a wheelchair. However, he or she wheels self and transfers alone, and is active in wheelchair about 12 hours a day.

7.5: The person is unable to take more than a few steps and is restricted to wheelchair, and may need aid to transfer. He or she wheels self, but may require a motorized chair for a full day's activities.

8.0: The person is essentially restricted to bed, a chair or a wheelchair, but may be out of bed much of day. He or she retains self care functions and has generally effective use of arms.

8.5: The person is essentially restricted to bed much of day, but has some effective use of arms and retains some self care functions.

9.0: The person is confined to bed, but still able to communicate and eat.

9.5: The person is totally helpless and bedridden and is unable to communicate effectively or eat and swallow.

10.0: Death due to MS.
